# Prioritization of causal genes for coronary artery disease based on cumulative evidence from experimental and *in silico* studies

**DOI:** 10.1038/s41598-020-67001-w

**Published:** 2020-06-26

**Authors:** Alexandra S. Shadrina, Tatiana I. Shashkova, Anna A. Torgasheva, Sodbo Z. Sharapov, Lucija Klarić, Eugene D. Pakhomov, Dmitry G. Alexeev, James F. Wilson, Yakov A. Tsepilov, Peter K. Joshi, Yurii S. Aulchenko

**Affiliations:** 1 Laboratory of Theoretical and Applied Functional Genomics, Novosibirsk State University, Novosibirsk, 630090 Russia; 2 Laboratory of Recombination and Segregation Analysis, Institute of Cytology and Genetics, Novosibirsk, 630090 Russia; 3 Department of Biological and Medical Physics, Moscow Institute of Physics and Technology, Moscow, 117303 Russia; 4 Research and Training Center on Bioinformatics, A.A. Kharkevich Institute for Information Transmission Problems, Moscow, 127051 Russia; 5 Genos Glycoscience Research Laboratory, Zagreb, Croatia; 6 MRC Human Genetics Unit, Institute of Genetics and Molecular Medicine, University of Edinburgh, Western General Hospital, Edinburgh, EH4 2XU Scotland UK; 7 Usher Institute, University of Edinburgh, Edinburgh, EH8 9AG Scotland UK; 8 PolyOmica, ‘s-Hertogenbosch, 5237 PA The Netherlands

**Keywords:** Coronary artery disease and stable angina, Disease genetics

## Abstract

Genome-wide association studies have led to a significant progress in identification of genomic loci affecting coronary artery disease (CAD) risk. However, revealing the causal genes responsible for the observed associations is challenging. In the present study, we aimed to prioritize CAD-relevant genes based on cumulative evidence from the published studies and our own study of colocalization between eQTLs and loci associated with CAD using SMR/HEIDI approach. Prior knowledge of candidate genes was extracted from both experimental and *in silico* studies, employing different prioritization algorithms. Our review systematized information for a total of 51 CAD-associated loci. We pinpointed 37 genes in 36 loci. For 27 genes we infer they are causal for CAD, and for 10 further genes we judge them most likely causal. Colocalization analysis showed that for 18 out of these loci, association with CAD can be explained by changes in gene expression in one or more CAD-relevant tissues. Furthermore, for 8 out of 36 loci, existing evidence suggested additional CAD-associated genes. For the remaining 15 loci, we concluded that evidence for gene prioritization remains inconsistent, insufficient, or absent. Our results provide deeper insights into the genetic etiology of CAD and demonstrate knowledge gaps where further research is warranted.

## Introduction

Coronary artery disease (CAD) is the most prevalent cardiovascular disease, the major cause of mortality and morbidity in both developed and developing countries^1^. This pathology is the manifestation of atherosclerosis in the coronary arteries. CAD can lead to a variety of complications, including chest pain, myocardial infarction (MI), arrhythmias and heart failure^2^. The etiology of CAD is multifactorial and involves a genetic predisposition as well as dietary and other lifestyle risk factors^3^. The genetic component to CAD has long been recognized. The Framingham Study demonstrated that positive family history is a strong risk factor for incident CAD^4–6^. According to Swedish and Danish twin studies, the narrow-sense heritability of fatal CAD is about 40–60%^7,8^. Today, it is widely accepted that much of the genetic component arises from the effect of many common alleles associated with modest increases in CAD risk^3,9^. Genome-wide association studies demonstrated that the common variation accounts for 40–50% of heritability of MI/CAD^10,11^.

Genetic studies of CAD started from family-based linkage studies discovering monogenic drivers of CAD and small candidate-gene studies which often provided controversial results. Development of high-throughput genotyping technologies and new statistical methods opened the era of genome-wide association studies (GWAS)^12,13^. MI was among the very first traits studied with use of genome-wide association strategy already in 2002^14^. Currently, more than 160 loci have been identified robustly associated with this condition^9,15^. The progress in this field has been fostered by establishing large international consortia, such as the Coronary ARtery DIsease Genome-wide Replication and Meta-analysis (CARDIoGRAM) Consortium, the Coronary Artery Disease (C4D) Genetics Consortium, and the Myocardial Infarction Genetics (MIGen) Consortium, as well as emergence of large biobanks containing genetic and clinical information, such as UK Biobank, and the development of haplotype reference panels for genotype imputation. In parallel, whole-exome and whole-genome sequencing studies revealed a set of CAD- and MI-promoting low-frequency variants^16–20^.

While we see major advances in unraveling genetic architecture of CAD, challenges remain in the annotation of causal genes at identified loci^9^. The largest proportion (90%) of SNP-based heritability of MI/CAD is explained by variants located in gene non-coding and intergenic regions, and only 10% resides within the gene coding regions^11^. Furthermore, many CAD-associated loci contain several genes. Thus, elucidating the gene responsible for the revealed association can be an arduous task. Filling the knowledge gaps on CAD-relevant genes is important for understanding biological mechanisms underlying this disease and translating GWAS results into novel treatment strategies.

Post-GWAS research, which aims at transition from GWAS signals to biological understanding, in particular identification of specific genes and pathways, involves both experimental and *in silico* studies^21^. The latter are less expensive and enable to narrow down the spectrum of candidate genes for subsequent experimental validation. A range of computational tools and approaches for *in silico* gene prioritization are currently available, including those based on data on the co-regulation of gene expression and reconstituted gene sets (DEPICT)^22^, potential relationships between the genes based on published scientific literature (GRAIL)^23^, functional annotation data from the Mouse Genome Database^24^, and others. An important tool for interpreting GWAS findings is the expression quantitative trait loci (eQTL) analysis^25^. Linking eQTL data with GWAS results can explain some of the associations by the presence of regulatory polymorphisms that influence the disease through altering gene expression in certain tissues. However, variants causative for the disease and changes in gene expression can simply be in linkage disequilibrium with each other, so identification of a joint SNP is on its own insufficient. This issue can be addressed using colocalization methods^26–29^. A method recently proposed by Zhu *et al*.^27^ involves summary data-based Mendelian randomization (SMR) analysis, which provides evidence for pleiotropy or causation with respect to the analyzed traits (e.g., disease and gene expression level), and heterogeneity in dependent instruments (HEIDI) test, which distinguishes pleiotropy/causation from linkage disequilibrium (LD).

In the present study, we pursued two objectives. First, we applied SMR/HEIDI approach to prioritize the genes at loci identified by two large genome-wide association meta-analyses^30,31^. Second, we performed an extensive literature search to find the genes within these loci linked to CAD in experimental studies or prioritized based on bioinformatics strategies. Our aim was to summarize and systematize this information and determine 1) the genes that can be considered causal/the most likely causal for CAD and 2) the loci for which CAD-associated genes remain unclear.

## Methods

### Selection of CAD-associated loci

We selected 51 loci robustly associated with CAD for which performing SMR/HEIDI analysis in our study was feasible. An algorithm we used to select the 51 loci is depicted in Supplementary Fig. S1. Loci were selected from two large mixed-ancestry genome-wide association meta-analyses: the study by Nikpay *et al*.^30^ (60,801 CAD cases and 123,504 controls) and the study by Howson *et al*.^31^ (88,192 CAD cases and 162,544 controls). The meta-analysis by Howson *et al*.^31^ included the CARDIoGRAMplusC4D study (63,746 CAD cases and 130,681 controls), and the meta-analysis by Nikpay *et al*.^30^ contained a subset of CARDIoGRAMplusC4D study participants (34,997 CAD cases and 49,512 controls). Thus, the samples analyzed in Nikpay *et al*.^30^ and Howson *et al*.^31^ studies contained 84,509 shared individuals. The study by Howson *et al*.^31^ was based on the CardioMetabochip^32^ lacking complete genomic coverage. The meta-analysis by Nikpay *et al*.^30^ comprised subjects genotyped with genome-wide SNP arrays and involved 1000 Genomes-based imputation. Howson *et al*. study^31^ was therefore nearly 1.4 times larger in size, while Nikpay *et al*. study^30^ had much higher SNP coverage (9.4 million imputed variants in Nikpay *et al*. study^30^ vs. 79,070 SNPs available for the meta-analysis in Howson *et al*. study^31^). In total, we extracted 61 loci from Howson *et al*. study^31^ and 35 loci from Nikpay *et al*. study^30^ associated with CAD at a statistical significance threshold of *P* < 5.0e-08.

SMR/HEIDI tests depend on the LD structure of the reference sample, so deriving summary statistics from mixed-ancestry cohorts is not appropriate. In our study, we focused on European ancestry individuals. We required the selected CAD-associated loci to reach at least suggestive level of statistical significance in the European ancestry datasets (that meant that at least one SNP in the region within ±250 kb around the lead SNP derived from the mixed-ancestry meta-analyses had to be associated with CAD at *P* < 5.0e-07 in Europeans, Supplementary Fig. S1). To check the loci selected from Howson *et al*. study^31^, we used summary statistics from Howson *et al*. meta-analysis that involved European-ancestry studies (N = 221,568). Since Nikpay *et al*.^30^ did not report GWAS results for European cohorts, for loci collected from that meta-analysis we used summary statistics from the previously published CARDIoGRAM study (Schunkert *et al*.^33^, 22,233 CAD cases and 64,762 controls of European descent; nearly 2.3 million imputed genotypes). Applying this criterion limited the number of selected loci to 50 in Howson *et al*. study^31^ and to 17 in Nikpay *et al*. study^30^, respectively (Supplementary Table S1a,b).

Finally, we matched the loci derived from both datasets. The loci were considered similar if the distance between the lead SNPs associated with CAD in Europeans was less than 250 kb (see Supplementary Fig. S1). All 17 loci selected from Nikpay *et al*.^30^/CARDIoGRAM^33^ studies partially overlapped with those derived from Howson *et al*. study^31^, and 16 of them were considered similar. Partially overlapping loci represented by lead SNPs rs3103349 (derived from Howson *et al*. study^31^) and rs10455872 (derived from CARDIoGRAM study^33^) did not meet our similarity criterion since the distance between SNPs rs3103349 and rs10455872 was 269 kb. Both loci were therefore included in the analysis. Thus, we selected a total of 51 loci (±250 kb from lead SNPs associated with CAD in the European datasets, Supplementary Fig. S1). The list of these loci is given in Supplementary Table S1c.

Summary statistics for CAD were obtained from the following resources: (1) the CARDIoGRAMplusC4D Consortium website (http://www.cardiogramplusc4d.org/; for data from Nikpay *et al*.^30^ and the CARDIoGRAM^33^ studies); (2) the PhenoScanner database (http://www.phenoscanner.medschl.cam.ac.uk; for data from Howson *et al*. study^31^; now these data are available in the GRASP repository^34^, https://grasp.nhlbi.nih.gov/FullResults.aspx). Data were downloaded in September 2017.

### SMR/HEIDI analysis

SMR/HEIDI approach^27^ was used to prioritize the genes within CAD-associated loci based on eQTL data. SMR/HEIDI compares patterns of SNP-trait associations in the loci between two GWAS (in our case, GWAS for CAD and GWAS for gene expression). The analysis includes several steps of SNP filtration. To pass the filtering, SNP must have the following properties: (1) being located in the studied locus; (2) present in both GWAS for CAD and in the analysis of expression quantitative trait loci (cis-eQTL results); (3) having MAF ≥ 0.03 in both datasets; (4) having squared Z-test value ≥ 10 in CAD GWAS. Those SNPs that meet criteria (1), (2), (3), (4) and have the lowest *P*-value for the association with CAD are used as instrumental variable to investigate relationships between the studied traits (hereinafter we define them as “top SNPs”).

SMR/HEIDI reveals the genes whose expression level may be affected by the same causal SNP that is associated with the studied condition. However, it is not able to identify this causal SNP. It can be either the top SNP or any other polymorphism in strong LD with this top SNP. Due to incomplete overlap between SNPs studied in different works, the top SNP does not necessarily represent a lead SNP within the locus that is associated with CAD or gene expression level at the highest level of statistical significance.

SMR/HEIDI tests were performed for a total of 51 loci (±250 kb from lead SNPs associated with CAD in the European datasets, Supplementary Fig. S1). GWAS summary statistics for CAD were derived from Howson *et al*. European-ancestry meta-analysis^31^ (N = 221,568). Summary statistics for eQTLs were obtained from three resources: GTEx version 7 database^35^ (https://gtexportal.org), CEDAR project^29^ (http://cedar-web.giga.ulg.ac.be/), and Westra Blood eQTL study^36^ (http://cnsgenomics.com/software/smr/#eQTLsummarydata). In total, we used data for 12 tissues and cell types: coronary and tibial artery, aorta, liver, and skeletal muscle (from the GTEx), whole blood (from the GTEx and Westra Blood eQTL), and circulating CD4 + T lymphocytes, CD8 + T lymphocytes, CD19 + B lymphocytes, CD14 + monocytes, CD15 + granulocytes, and platelets (from CEDAR). We selected coronary and tibial artery, aorta, liver, and skeletal muscle tissue for the analysis because these tissues were suggested as genetically causal for CAD^37^. Whole blood and peripheral blood mononuclear cells/platelets were selected since atherosclerotic plaques are in direct contact with blood flow. Mononuclear cells infiltrate the plaques, mediate inflammatory response and participate in atherosclerosis development and progression and also trigger the thrombotic complications^38,39^. Platelets adhere to the damaged arteries and form mural thrombi^40^. These cells contribute to atherosclerotic inflammation by releasing a number of immune-related molecules and facilitating inflammatory cells recruitment^41^.

We considered that expression of a certain gene may be influenced by the same functional polymorphism as that altering the CAD risk in case SMR test FDR was <0.05 and the *P*-value in the HEIDI test was ≥0.001. The tests were performed only if the number of SNPs eligible for the analysis was ≥3. Maximal number of SNPs in the analysis was twenty. Other technical details of our SMR/HEIDI implementation are given in Supplementary Methods.

SMR/HEIDI analysis was performed using the GWAS-MAP platform^42^. GWAS-MAP platform integrates an embedded software for SMR/HEIDI analysis^27^, theta metric-based approach proposed by Momozawa *et al*.^29^, LD Score regression^43^, and two-sample Mendelian randomization analysis (MR-Base package^44^). It also integrates a database containing GWAS summary statistics for eQTLs from GTEx^35^, CEDAR^29^, and Westra Blood eQTL study^36^ and summary-level GWAS results for 123 metabolomics traits, 2,453 complex traits from the UK Biobank^45^, and 10 traits related to coronary artery disease and associated conditions. Further details on the platform are given in Supplementary Methods.

### Extraction of data on CAD-related genes from the previous studies

Our pipeline of extracting data on the genes potentially related to CAD from the previous studies is provided in Supplementary Fig. S2.

We performed a literature search in Pubmed, Google Scholar, and the Online Mendelian Inheritance in Man database (OMIM, https://www.omim.org/) in order to find the genes for which evidence from “wet” (*in vivo*, *in vitro*) experimental studies suggests their role in CAD. Only those genes were checked that are located in the 51 studied loci (±250 kb from lead SNPs listed in Supplementary Table S1c) according to the NCBI Gene database (https://www.ncbi.nlm.nih.gov/gene). For each gene that we considered to be potentially functionally related to CAD, we made a brief literature review.

We also extracted data on the prioritized genes from four previously published *in silico* studies^46–49^. A brief summary of approaches used in that studies is provided in Supplementary Methods. Three studies (by Brænne *et al*.^46^, Lempiäinen *et al*.^47^, and van der Harst *et al*.^48^) prioritized potentially causal CAD-associated SNPs and linked them with the candidate genes. In two of these studies^46,47^, the genes were scored (higher score assigned to the gene corresponded to stronger evidence for its implication in CAD based on the prioritization pipeline used), and potentially causal SNP-gene annotations with the scores were listed in Supplementary data provided with these articles. In the study by van der Harst *et al*.^48^, no scores were assigned to the genes, but the genes were specifically highlighted for which converging evidence of a potential functional SNP-gene mechanism was observed. We used the following protocol to extract data from the studies^46–48^: first, we obtained information on chromosome positions of each prioritized SNP from the NCBI SNP database (https://www.ncbi.nlm.nih.gov/snp/); second, we checked whether these SNPs are located in the 51 studied loci, and if yes, we attributed the gene prioritized with these SNPs to those loci that contained these prioritized SNPs.

The fourth *in silico* study by Svishcheva *et al*.^49^ applied methods of gene-based association analysis using two large datasets (the UK Biobank data and Myocardial Infarction Genetics and CARDIoGRAM Exome meta-analysis). We checked whether CAD-associated genes revealed in that study are located in 51 loci selected for our analysis. If yes, we attributed them to the corresponding loci.

In order to systematize information from all studies, we made a table containing data on 51 loci, including rs-identifier and chromosomal position of the lead SNP, the gene nearest to the lead SNP (according to the NCBI SNP database), the genes revealed in SMR/HEIDI analysis, the genes prioritized in previous studies (with the scores where applicable) and candidate genes found in literature resources. Based on cumulative evidence from multiple sources, we concluded, which genes can be considered causal/the most likely causal for CAD, for which loci the role of additional genes can be proposed, and for which loci no conclusion can be made, or evidence is absent.

It should be noted that in our study, we did not investigate whether the analyzed loci contain single or multiple association signals. Thus, when choosing SNPs from the studies by Brænne *et al*.^46^, Lempiäinen *et al*.^47^, and van der Harst *et al*.^48^, we did not make any restrictions on the LD between SNP prioritized in that works and the lead GWAS SNPs. Similarly, we did not limit the top SNPs in the SMR/HEIDI analysis (the instrumental variable used for investigating relationships between CAD and gene expression) only to those which are in LD with the lead SNPs. However, we analyzed LD between all SNPs linked to genes at each locus using LDlink online tool (https://analysistools.nci.nih.gov/LDlink/; data were obtained for European-ancestry populations) or PLINK 1.9 software (https://www.cog-genomics.org/plink2/, based on 1000 Genomes phase 3 version 5 data for European ancestry individuals with MAF ≥ 0.03 filtration). Cases where multiple signals were strongly suspected were discussed separately.

## Results

### SMR/HEIDI analysis

We found 83 probes (related to 73 protein-coding genes, 2 pseudogenes, 7 noncoding RNAs, and one uncharacterized probe *HS.443185*; listed in Table 1), whose expression levels in CAD-relevant tissues and cells (coronary and tibial artery, aorta, liver, skeletal muscle^37^, blood, circulating lymphocytes, monocytes, granulocytes, and platelets) are associated with the same causal variants that account for the association between 32 out of 51 studied loci and CAD with FDR_SMR_ < 0.05 and *P*_HEIDI_ ≥ 0.001. Full results of SMR/HEIDI analysis are presented in Supplementary Table S2. As far as we are aware, 29 of these genes – *PSMA5* (locus #2), *DDX59-AS1* (locus #5), *USP39* and *GNLY* (locus #10), *FAM117B* (locus #12), *NME9* and *ESYT3* (locus #13), *RP1-257A7.4* and *RP1-257A7.5* (locus #18), *RP1-283K11.3* and *RP3-323P13.2* (locus #20), *IFIT1* and *IFIT5* (locus #32), *TMEM180* and *ARL3* (locus #33), *MAP3K11*, *CTSW*, and *FIBP* (locus #34), *RP11-563P16.1* (locus #35), *RP3-462E2.3* (locus #36), *ERP29* (locus #37), *OASL* and *COQ5* (locus #38), *MORF4L1* (locus #43), *PKD1L3*, *DHX38*, and *DHODH* (locus #45), *C19ORF52* (locus #49), and *EDEM2* (locus #50) – have never been previously proposed as candidate genes for CAD.

**Table 1:** Genes in 51 CAD-associated loci (±250 kb around the lead SNP) proposed to be causal according to different lines of evidence.

	Lead SNP^¥^	Chr: position^*^	Nearest^†^ known gene	Genes prioritized by SMR/HEIDI^‡^	Candidate genes from literature^**^	Genes prioritized previously based on bioinformatics approaches^46–48^ and found in gene-based association analysis^49^	Conclusion
1	rs17114036	1: 56 962 821	*PLPP3 (PAP2B, PPAP2B)*	—	▪ ***PLPP3 (PAP2B, PPAP2B)***	▪ **Lempiäinen** ***et al****.*^47^**, score range 2-54** *PLPP3 (PAP2B)* (total score = 10) ▪ **Svishcheva** ***et al****.*^49^ *PLPP3 (PAP2B)* (two datasets)	*PLPP3 (PAP2B)* is the causal gene.
2	rs602633	1: 109 821 511	*PSRC1*	▪ *PSRC1* ← ▪ *CELSR2* ← ▪ *PSMA5* ←	▪ ***SORT1*** ▪ *PSRC1* ▪ *CELSR2*	**Brænne** ***et al****.*^46^**, score range 1-11** ▪ *CELSR2* (total score = 5) **←** ▪ *SORT1* (total score = 4) ← ▪ *PSRC1* (total score = 4) ← ▪ *MYBPHL* (total score = 2) **Lempiäinen** ***et al****.*^47^**, score range 2-54** ▪ *CELSR2* (total score = 10) ← **van der Harst** ***et al****.*^48^ ▪ *SORT1*^¶^ **←** ▪ *CELSR2* **←** ▪ *PSRC1* **←** ▪ *SARS* **←** ▪ *ATXN7L2* **←** **Svishcheva** ***et al****.*^49^ ▪ *CELSR2* (two datasets)	*SORT1* is the causal gene. *PSRC1* and *CELSR2* might also be involved.
3	rs4129267	1: 154 426 264	*IL6R*	▪ *IL6R* **←**, **← ←**	▪ ***IL6R***	**Brænne** ***et al****.*^46^**, score range 1-11** ▪ *IL6R* (total score = 5) **←** ▪ *UBAP2L* (total score = 2) **←** ▪ *ATP8B2* (total score = 2) **←** ▪ *CHTOP* (total score = 1) **←** **Lempiäinen** ***et al****.*^47^**, score range 2-54** ▪ *IL6R* (total score = 10) **←**, **← ←**	*IL6R* is the causal gene.
4	rs10919065	1: 169 093 557	*ATP1B1*	▪ *ATP1B1* **← ←** ▪ *NME7* **←**, **← ←**	▪ *ATP1B1*	**Brænne** ***et al****.*^46^**, score range 1-11** ▪ *ATP1B1* (total score = 4) **←** ▪ *NME7* (total score = 2) **←** ▪ *CCDC181* (total score = 1) **←**	*ATP1B1* is the most likely causal gene.
5	rs6700559	1: 200 646 073	*DDX59-AS1*	▪ *DDX59-AS1 (RP11-92G12.3)* **←** ▪ *DDX59* **←** ▪ *CAMSAP2 (CAMSAP1L1)* **←**	—	**Brænne** ***et al****.*^46^**, score range 1-11** ▪ *KIF14* (total score = 4) **←** ▪ *CAMSAP2* (total score = 2) **←** ▪ *DDX59* (total score = 2) **←** **van der Harst** ***et al****.*^48^ ▪ *CAMSAP2*^¶^ **←** ▪ *DDX59* **←**	Evidence is inconsistent.
6	rs2820315	1: 201 872 264	*LMOD1*	▪ *IPO9* **←** ▪ *LMOD1* **←**	▪ *LMOD1*	**Brænne** ***et al****.*^46^**, score range 1-11** ▪ *IPO9* (total score = 4) **←** ▪ *LMOD1* (total score = 2) **←** ▪ *SHISA4* (total score = 1) **←** **Lempiäinen** ***et al****.*^47^**, score range 2-54** ▪ *IPO9* (total score = 10) **←**	Evidence is inconsistent. *LMOD1* and *IPO9* can be involved.
7	rs16986953	2: 19 942 473	*LINC00954*	—	—	—	No evidence.
8	rs515135	2: 21 286 057	*APOB*	—	▪ ***APOB***	**Lempiäinen** ***et al****.*^47^**, score range 2-54** ▪ *APOB* (total score = 32) **Svishcheva** ***et al****.*^49^ ▪ *APOB* (one dataset)	*APOB* is the causal gene.
9	rs6544713	2: 44 073 881	*ABCG8*	—	▪ ***ABCG8*** ▪ ***ABCG5***	**Lempiäinen** ***et al****.*^47^**, score range 2-54** ▪ *ABCG8* (total score = 34) **Svishcheva** ***et al****.*^49^ ▪ *ABCG8* (two datasets)	*ABCG8/ABCG5* are the causal genes.
10	rs1561198	2: 85 809 989	*VAMP8*	▪ *GGCX* **←** ▪ *VAMP5* **←** ▪ *VAMP8* **←** ▪ *USP39* **←** ▪ *GNLY* **←**	▪ *GGCX* ▪ *VAMP8*	**Brænne** ***et al****.*^46^**, score range 1-11** ▪ *GGCX* (total score = 5) **←** ▪ *VAMP5* (total score = 5) **←** ▪ *VAMP8* (total score = 5) **←** **Lempiäinen** ***et al****.*^47^**, score range 2-54** ▪ *VAMP8* (total score = 42) **←** **Svishcheva** ***et al****.*^49^ ▪ *MAT2A* (one dataset) ▪ *GGCX* (one dataset) ▪ *VAMP5* (one dataset)	Evidence is inconsistent.
11	rs2252641	2: 145 801 461	*TEX41*	—	▪ *ZEB2*	**Lempiäinen** ***et al****.*^47^**, score range 2-54** ▪ *TEX41* (total score = 2)	Evidence is inconsistent.
12	rs2351524	2: 203 880 992	*NBEAL1*	▪ *ICA1L* **←** ▪ *CARF* **←** ▪ *NBEAL1* **←** ▪ *FAM117B* **←**	▪ *WDR12*	**Brænne** ***et al****.*^46^**, score range 1-11** ▪ *NBEAL1* (total score = 4) **←** ▪ *WDR12* (total score = 4) **←** ▪ *CARF* (total score = 3) **←** ▪ *ALS2CR8* (total score = 2) **←** ▪ *ICA1L* (total score = 1) **←** **Lempiäinen** ***et al****.*^47^**, score range 2-54** ▪ *ICA1L* (total score = 10) **←** **Svishcheva** ***et al****.*^49^ ▪ *NBEAL1* (two datasets) ▪ *WDR12* (two datasets)	Evidence is inconsistent.
13	rs2306374	3: 138 119 952	*MRAS*	▪ *MRAS* **←** ▪ *NME9* **←** ▪ *ESYT3* **←**	▪ *MRAS*	**Brænne** ***et al****.*^46^**, score range 1-11** ▪ *MRAS* (total score = 5) **←** ▪ *CEP70* (total score = 2) **←** **Lempiäinen** ***et al****.*^47^**, score range 2-54** ▪ *MRAS* (total score = 34) **←** **Svishcheva** ***et al****.*^49^ ▪ *MRAS* (one dataset)	*MRAS* is the causal gene.
14	rs1429141	4: 148 288 067	*MIR548G*	—	▪ *EDNRA (ETA)*	**Lempiäinen** ***et al****.*^47^**, score range 2-54** ▪ *EDNRA (ETA)* (total score = 34) **Svishcheva** ***et al****.*^49^ ▪ *EDNRA (ETA)* (one dataset)	*EDNRA* is the causal gene.
15	rs7692387	4: 156 635 309	*GUCY1A1*	▪ *GUCY1A3* **←**	▪ ***GUCY1A3***	**Lempiäinen** ***et al****.*^47^**, score range 2-54** ▪ *GUCY1A3* (total score = 42) **←**	*GUCY1A3* is the causal gene.
16	rs273909	5: 131 667 353	*SLC22A4* *MIR3936HG*	—	—	**Lempiäinen** ***et al****.*^47^**, score range 2-54** ▪ *SLC22A5* (total score = 10)	Insufficient evidence.
17	rs246600	5: 142 516 897	*ARHGAP26*	—	—	**van der Harst** ***et al****.*^48^ ▪ *HMHB1*	Insufficient evidence.
18	rs7751826	6: 12 900 977	*PHACTR1*	▪ *RP1-257A7.5* **←** ▪ *RP1-257A7.4* **←** ▪ *PHACTR1* **←**	▪ ***PHACTR1***	**Lempiäinen** ***et al****.*^47^**, score range 2-54** ▪ *PHACTR1* (total score = 2) **←, ← ←** **van der Harst** ***et al****.*^48^ ▪ *EDN1*^¶^ **← ←** ▪ *TBC1D7* **← ←** ▪ *PHACTR1* **← ←** ▪ *GFOD1* **← ←** **Svishcheva** ***et al****.*^49^ ▪ *PHACTR1* (two datasets)	*PHACTR1* is the causal gene.
19	rs10947789	6: 39 174 922	*KCNK5*	—	—	**Lempiäinen** ***et al****.*^47^**, score range 2-54** ▪ *KCNK5* (total score = 2)	Insufficient evidence.
20	rs2327429	6: 134 209 837	*TARID*	▪ *TCF21* **←** ▪ *RP3-323P13.2* **←** ▪ *RP1-283K11.3* **←**	▪ ***TCF21***	**Lempiäinen** ***et al****.*^47^**, score range 2-54** ▪ *TCF21* (total score = 10) **←**	*TCF21* is the causal gene. *RP3-323P13.2* might also be involved.
21	rs3103349^#^	6: 160 740 721	*SLC22A3*	▪ *LPA*	▪ ***LPA (APOA)***	**Brænne** ***et al****.*^46^**, score range 1-11** ▪ *SLC22A3* (total score = 5) **← ←** ▪ *AL591069.5* (total score = 1) **← ←** **Lempiäinen** ***et al****.*^47^**, score range 2-54** ▪ *LPA* (total score = 54) ▪ *LPAL2 pseudogene* (total score = 10) **← ←** ▪ *IGF2R* (total score = 2) ▪ *SLC22A2* (total score = 2) ▪ *SLC22A3* (total score = 2) **Svishcheva** ***et al****.*^49^ ▪ *LPA* (two datasets) ▪ *SLC22A1* (two datasets) ▪ *SLC22A2* (two datasets) ▪ *SLC22A3* (two datasets) ▪ *IGF2R* (one dataset)	*LPA* is the causal gene. *SLC22A3, SLC22A2, SLC22A1* might also be involved.
22	rs10455872^#^	6: 161 010 118	*LPA*	▪ *LPA* **← ←**	▪ ***LPA (APOA)***	**Brænne** ***et al****.*^46^**, score range 1-11** ▪ *PLG* (total score = 6) **←** ▪ *SLC22A3* (total score = 5) **← ←** ▪ *LPAL2 pseudogene* (total score = 4) **←** ▪ *AL591069.5* (total score = 1) **← ←** **Lempiäinen** ***et al****.*^47^**, score range 2-54** ▪ *LPA* (total score = 54) ▪ *PLG* (total score = 46) **←** ▪ *LPAL2 pseudogene* (total score = 10) **← ←** ▪ *SLC22A3* (total score = 2) **Svishcheva** ***et al****.*^49^ ▪ *SLC22A3* (two datasets) ▪ *LPA* (two datasets) ▪ *PLG* (two datasets)	*LPA* is the causal gene. *PLG* and *SLC22A3* might also be involved.
23	rs11556924	7: 129 663 496	*ZC3HC1(NIPA)*	▪ *KLHDC10* **←**	▪ *KLHDC10* ▪ *ZC3HC1 (NIPA)*	**Brænne** ***et al****.*^46^**, score range 1-11** ▪ *ZC3HC1 (NIPA)* (total score = 4) **←** **Lempiäinen** ***et al****.*^47^**, score range 2-54** ▪ *ZC3HC1 (NIPA)* (total score = 22) **←** **van der Harst** ***et al****.*^48^ ▪ *ZC3HC1*^¶^ **←** ▪ *NRF1* **←** ▪ *KLF14* **←** **Svishcheva** ***et al****.*^49^ ▪ *ZC3HC1* (one dataset)	*ZC3HC1 (NIPA)* is the most likely causal gene. *KLHDC10* might also be involved.
24	rs10237377	7: 139 757 136	*PARP12*	—	▪ *TBXAS1*	**Brænne** ***et al****.*^46^**, score range 1-11** ▪ *TBXAS1* (total score = 5) **Lempiäinen** ***et al****.*^47^**, score range 2-54** ▪ *PARP12* (total score = 10) **←** ▪ *TBXAS1* (total score = 10) **←**	*TBXAS1* is the most likely causal gene.
25	rs11204085	8: 19 940 796	*SLC18A1*	▪ *LPL*	▪ ***LPL***	**Brænne** ***et al****.*^46^**, score range 1-11** ▪ *LPL* (total score = 8) **←** **Lempiäinen** ***et al****.*^47^**, score range 2-54** ▪ *LPL* (total score = 42) **←** **Svishcheva** ***et al****.*^49^ ▪ *LPL* (one dataset)	*LPL* is the causal gene.
26	rs2954032	8: 126 493 392	*TRIB1*	—	▪ ***TRIB1***	—	*TRIB1* is the causal gene.
27	rs3218020	9: 21 997 872	*CDKN2B-AS1 (ANRIL)*	—	▪ ***CDKN2B-AS1 (ANRIL)***	**Brænne** ***et al****.*^46^**, score range 1-11** ▪ *CDKN2B* (total score = 8) **←, ← ←** ▪ *CDKN2A* (total score = 5) **←** **Lempiäinen** ***et al****.*^47^**, score range 2-54** ▪ *CDKN2B* (total score = 9) **←** ▪ *CDKN2B-AS1* (total score = 2) **← ←** **van der Harst** ***et al****.*^48^ ▪ *CDKN2B*^¶^ **← ← ←** ▪ *MTAP***← ← ←** **Svishcheva** ***et al****.*^49^ ▪ *CDKN2B* (two datasets) ▪ *CDKN2A* (two datasets) ▪ *MTAP* (one dataset)	*CDKN2B-AS1* is the causal gene, which regulates *CDKN2B* and *CDKN2A* expression.
28	rs579459	9: 136 154 168	*ABO*	▪ *SURF1* **←** ▪ *ABO* **←**	▪ *ABO* ▪ *ADAMTS13*	**Lempiäinen** ***et al****.*^47^**, score range 2-54** ▪ *DDX31* (total score = 8) **←** ▪ *SURF1* (total score = 8) **←** ▪ *SURF6* (total score = 8) **←** **Svishcheva** ***et al****.*^49^ ▪ *ABO* (one dataset) ▪ *ADAMTS13* (one dataset)	Evidence is inconsistent.
29	rs2505083	10: 30 335 122	*JCAD* *(KIAA1462)*	▪ *JCAD (KIAA1462)* **←**	▪ *JCAD (KIAA1462)*	**Brænne** ***et al****.*^46^**, score range 1-11** ▪ *JCAD (KIAA1462)* (total score = 6) **←** **Lempiäinen** ***et al****.*^47^**, score range 2-54** ▪ *JCAD (KIAA1462)* (total score = 6) **←** **Svishcheva** ***et al****.*^49^ ▪ *JCAD (KIAA1462)* (one dataset)	*JCAD* is the causal gene.
30	rs10793513	10: 44 494 546	*LINC00841*	—	—	—	No evidence.
31	rs523297	10: 44 756 557	*CXCL12*	—	▪ ***CXCL12***	—	*CXCL12* is the causal gene.
32	rs2246833	10: 91 005 854	*LIPA*	▪ *LIPA* **←** ▪ *IFIT1* **←** ▪ *IFIT5* **←**	▪ ***LIPA***	**Brænne** ***et al****.*^46^**, score range 1-11** ▪ *LIPA* (total score = 9) **←** **Lempiäinen** ***et al****.*^47^**, score range 2-54** ▪ *LIPA* (total score = 46) **←** **Svishcheva** ***et al****.*^49^ ▪ *LIPA* (one dataset)	*LIPA* is the causal gene.
33	rs11191447	10: 104 652 323	*BORCS7-ASMT* *AS3MT*	▪ *TMEM180 (MFSD13A)* **←** ▪ *ARL3* **←** ▪ *NT5C2* **←** ▪ *MARCKSL1P1 pseudogene* **←**	▪ *CYP17A1*	**Brænne** ***et al****.*^46^**, score range 1-11** ▪ *NT5C2* (total score = 5) **←** ▪ *CNNM2* (total score = 4) **←** **Lempiäinen** ***et al****.*^47^**, score range 2-54** ▪ *CYP17A1* (total score = 34) **←** **Svishcheva** ***et al****.*^49^ ▪ *CYP17A1* (one dataset) ▪ *CNNM2* (one dataset) ▪ *AS3MT* (one dataset)	*CYP17A1* is the most likely causal gene.
34	rs12801636	11: 65 391 317	*PCNX3*	▪ *SIPA1* **←** ▪ *MAP3K11* **←** ▪ *CTSW* **← ←** ▪ *FIBP* **← ←**	▪ *RELA*	**Brænne** ***et al****.*^46^**, score range 1-11** ▪ *RELA* (total score = 6) **←** ▪ *SIPA1* (total score = 4) **←** *OVOL1* (total score = 2) **←** ▪ *PCNXL3* (total score = 1) **←** **Lempiäinen** ***et al****.*^47^**, score range 2-54** ▪ *RELA* (total score = 40) **←** **van der Harst** ***et al****.*^48^ ▪ *EHBP1L1*^¶^ **←**	Evidence is inconsistent.
35	rs974819	11: 103 660 567	*MIR4693*	▪ *PDGFD* **←** ▪ *RP11-563P16.1* **←**	▪ ***PDGFD***	**van der Harst** ***et al****.*^48^ ▪ *PDGFD*^¶^ **←**	*PDGFD* is the causal gene.
36	rs3184504^§^	12: 111 884 608	*SH2B3 (LNK)*	▪ *SH2B3* **← ←** ▪ *TMEM116* **←** ▪ *ALDH2* **←** ▪ *MAPKAPK5* **←** ▪ *RP3-462E2.3* **←**	▪ *SH2B3 (LNK)* ▪ *ATXN2*	**Brænne** ***et al****.*^46^**, score range 1-11** ▪ *SH2B3* (total score = 5) **← ←** ▪ *ATXN2* (total score = 4) **← ←** ▪ *FLJ21127* (total score = 1) **← ←** **Lempiäinen** ***et al****.*^47^**, score range 2-54** ▪ *SH2B3* (total score = 14) **← ←** **Svishcheva** ***et al****.*^49^ ▪ *ATXN2* (two datasets) ▪ *SH2B3* (one dataset)	*SH2B3* is the most likely causal gene. *ATXN2* might also be involved.
37	rs441^§^	12: 112 228 849	*ALDH2*	▪ *TMEM116* **←** ▪ *ERP29* **←** ▪ *SH2B3* ▪ *ALDH2* **←** ▪ *MAPKAPK5* **←**	▪ *ATXN2* ▪ *ALDH2* ▪ *MAPKAPK5*	**Brænne** ***et al****.*^46^**, score range 1-11** ▪ *ALDH2* (total score = 6) **←** ▪ *SH2B3* (total score = 5) **←** ▪ *TMEM116* (total score = 4) **←** ▪ *BRAP* (total score = 4) **←** ▪ *MAPKAPK5* (total score = 4) **←** ▪ *HECTD4* (total score = 2) **←** ▪ *C12ORF30* (total score = 2) **←** **Svishcheva** ***et al****.*^49^ ▪ *ATXN2* (two datasets) ▪ *TMEM116* (one dataset) ▪ *NAA25* (one dataset)	Evidence is inconsistent.
38	rs2258287	12: 121 454 313	*C12ORF43*	▪ *OASL* **←** ▪ *C12ORF43* **←** ▪ *COQ5*	▪ *HNF1A*	**Brænne** ***et al****.*^46^**, score range 1-11** ▪ *HNF1A* (total score = 4) **← ←** **Lempiäinen** ***et al****.*^47^**, score range 2-54** ▪ *C12ORF43* (total score = 8) **← ←**	*HNF1A* is the most likely causal gene.
39	rs11057830	12: 125 307 053	*SCARB1*	—	▪ *SCARB1*	**Brænne** ***et al****.*^46^**, score range 1-11** ▪ *SCARB1* (total score = 6) **←** **Lempiäinen** ***et al****.*^47^**, score range 2-54** ▪ *SCARB1* (total score = 34) **←** ▪ *DHX37* (total score = 2) **Svishcheva** ***et al****.*^49^ ▪ *SCARB1* (one dataset)	*SCARB1* is the causal gene.
40	rs9319428	13: 28 973 621	*FLT1 (VEGFR1)*	—	▪ *FLT1 (VEGFR1)*	**Lempiäinen** ***et al****.*^47^**, score range 2-54** ▪ *FLT1* (total score = 34)	*FLT1* is the causal gene.
41	rs9515203	13: 111 049 623	*COL4A2*	—	▪ *COL4A2, COL4A1*	**Brænne** ***et al****.*^46^**, score range 1-11** ▪ *IRS2* (total score = 4) **Lempiäinen** ***et al****.*^47^**, score 2-54** ▪ *ANKRD10* (total score = 8) **←** ▪ *COL4A1* (total score = 2) **← ←** ▪ *COL4A2* (total score = 2) **←, ← ←** **Svishcheva** ***et al****.*^49^ ▪ *COL4A2* (two datasets) ▪ *COL4A1* (one dataset)	*COL4A2* and *COL4A1* are the causal genes.
42	rs2895811	14: 100 133 942	*HHIPL1*		▪ *HHIPL1*	**Brænne** ***et al****.*^46^**, score range 1-11** ▪ *YY1* (total score = 6) **←** ▪ *EML1* (total score = 2) **Lempiäinen** ***et al****.*^47^**, score 2-54** ▪ *HHIPL1* (total score = 6) **←**	*HHIPL1* is the most likely causal gene.
43	rs7178051	15: 79 118 296	*ADAMTS7*	▪ *ADAMTS7* **←** ▪ *CTSH* **←** ▪ *RP11-160C18.2 pseudogene* **←** ▪ *MORF4L1* **←**	▪ ***ADAMTS7***	**Brænne** ***et al****.*^46^**, score range 1-11** ▪ *ADAMTS7* (total score = 7) **← ←**, **← ← ←** ▪ *WDR61* (total score = 2) **← ←** **Lempiäinen** ***et al****.*^47^**, score range 2-54** ▪ *ADAMTS7* (total score = 38) **← ← ←** ▪ *CTSH* (total score = 8) **van der Harst** ***et al****.*^48^ ▪ *ADAMTS7* **← ← ← ←** ▪ *RASGRF1* **← ← ← ←** **Svishcheva** ***et al****.*^49^ ▪ *ADAMTS7* (two datasets)	*ADAMTS7* is the causal gene.
44	rs17514846	15: 91 416 550	*FURIN*	▪ *FURIN* **←** ▪ *FES* **←** ▪ *MAN2A2* **←**	▪ *FURIN*	**Brænne** ***et al****.*^46^**, score range 1-11** ▪ *FURIN* (total score = 8) **←** ▪ *FES* (total score = 7) **←** ▪ *MAN2A2* (total score = 3) **←** **Lempiäinen** ***et al****.*^47^**, score range 2-54** ▪ *FURIN* (total score = 10) **←** ▪ *FES* (total score = 10) **←** **Svishcheva** ***et al****.*^49^ ▪ *FURIN* (two datasets) ▪ *FES* (one dataset)	*FURIN* is the causal gene. *FES* might also be involved.
45	rs1050362	16: 72 130 815	*DHX38*	▪ *HP* **←** ▪ *DHX38* **←** ▪ *DHODH* **←** ▪ *PKD1L3* **←**	▪ *HP*	**Svishcheva** ***et al****.*^49^ ▪ *HPR* (one dataset)	*HP* is the most likely causal gene.
46	rs170041	17: 2 170 216	*SMG6*	—	▪ *SMG6* ▪ *SRR*	**Lempiäinen** ***et al****.*^47^**, score range 2-54** ▪ *SRR* (total score = 8) **Svishcheva** ***et al****.*^49^ ▪ *SMG6* (two datasets)	Evidence is inconsistent.
47	rs12936587	17: 17 543 722	*RAI1*	▪ *SREBF1 (SREBP1)* **←** ▪ *PEMT* **←**	▪ *PEMT* ▪ *SREBF1 (SREBP1)* ▪ *MIR33B (hsa-mir-33b)*	**Lempiäinen** ***et al****.*^47^**, score range 2-54** ▪ *SREBF1* (total score = 40) **←** ▪ *PEMT* (total score = 40) **←**	Evidence is inconsistent. *PEMT, SREBF1*, and *MIR33B* can be involved.
48	rs2070783	17: 62 406 971	*PECAM1*	▪ *PECAM1* **←**	▪ *PECAM1*	**Brænne** ***et al****.*^46^**, score range 1-11** ▪ *PECAM1* (total score = 4) **←** ▪ *POLG2* (total score = 3) **←**	*PECAM1* is the causal gene.
49	rs12052058	19: 11 159 525	*SMARCA4*	▪ *SMARCA4* **←** ▪ *CARM1* **←** ▪ *C19ORF52* **←** ▪ *KANK2*	▪ ***LDLR*** ▪ *SMARCA4* ▪ *CARM1*	**Brænne** ***et al****.*^46^**, score range 1-11** ▪ *KANK2* (total score = 5) **← ←** ▪ *SMARCA4* (total score = 4) **←** ▪ *ANKRD25* (total score = 2) **← ←** **Lempiäinen** ***et al****.*^47^**, score range 2-54** ▪ *LDLR* (total score = 35) **←** ▪ *CARM1* (total score = 40) **←, ← ← ←** ▪ *SMARCA4* (total score = 40) **←, ← ← ←** ▪ *C19ORF38* (total score = 10) **← ← ←** **Svishcheva** ***et al****.*^49^ ▪ *LDLR* (two datasets) ▪ *SMARCA4* (one dataset)	*LDLR* is the causal gene. *SMARCA4* and *CARM1* might also be involved.
50	rs867186	20: 33 764 554	*PROCR*, *MMP24-AS1-EDEM2*	▪ *TRPC4AP* **←** ▪ *EIF6* **←** ▪ *ITGB4BP* **←** ▪ *EDEM2* **← ←** ▪ *HS.443185* **← ←**	▪ *PROCR (EPCR)*	**Brænne** ***et al****.*^46^**, score range 1-11** ▪ *PROCR* (total score = 8) **←** ▪ *MYH7B* (total score = 5) **←** ▪ *TRPC4AP* (total score = 3) **←** ▪ *EIF6* (total score = 3) **←** ▪ *RBL1* (total score = 3) **←** ▪ *ROMO1* (total score = 2) **←** ▪ *ITGB4BP* (total score = 2) **←** ▪ *FLJ25841* (total score = 1) **←** ▪ *MT1P3* (total score = 1) **←** **van der Harst** ***et al****.*^48^ ▪ *PROCR*^¶^ **←** ▪ *TRPC4AP*^¶^ **←** ▪ *GGT7* **←** ▪ *EDEM2* **←** ▪ *NCOA6* **←** ▪ *HMGB3P1* **←**	*PROCR* is the most likely causal gene.
51	rs9982601	21: 35 599 128	*LINC00310*	▪ *MRPS6* ▪ *KCNE2*	▪ *KCNE2 (MIRP1)*	**Lempiäinen** ***et al****.*^47^**, score range 2-54** ▪ *SON* (total score = 8) **van der Harst** ***et al****.*^48^ ▪ *MRPS6*^¶^ **←** ▪ *SLC5A3*^¶^ **←**	*KCNE2* is the most likely causal gene.

Alternative gene names or non-coding RNA names are given in parenthesis after official gene symbols. **Literature overview for each candidate gene found in literature sources is provided in Supplementary Table** S4. In the studies^46–49^, possible candidate genes were linked to the prioritized CAD-associated SNPs (data on those SNPs located in the^51^ studied loci can be found in Supplementary Table S3b). Arrows near gene names indicate that these genes have been linked to the same prioritized SNP in the locus or to SNPs in high LD with each other (r^2^ ≥ 0.8; Supplementary Table S3c). If there are two or more groups of such genes in the locus, single arrow indicates the genes linked to one SNP; double, triple, and quadruple arrows – genes linked to other SNPs (e.g., in locus 43). We also marked with arrows the genes found in SMR/HEIDI analysis if the top SNP (instrumental variable used for investigating relationships between gene expression and CAD) was the same or in high LD (r^2^ ≥ 0.8; Supplementary Table S3d) with SNPs prioritized in other studies.

^¥^Loci for the analysis in our study were defined as regions within ±250 kb around these lead SNPs (see Supplementary Table S1c).

^*^Chromosome: position of the lead SNP on the chromosome according to GRCh37.p13

^†^Nearest gene according to the NCBI dbSNP database (https://www.ncbi.nlm.nih.gov/snp/)

^‡^Information on whether increased gene expression in CAD-relevant tissue is associated with the increased or decreased CAD risk is given in Supplementary Table S2a.

^**^Candidate genes with the most compelling evidence for their role in CAD according to literature data are shown in bold.

^¶^Converging evidence of a potential functional SNP-gene mechanism (demonstrated in the study by van der Harst *et al*.^48^).

^#, §^These pairs of loci are overlapping and contain partially the same genes. Since the distance between the lead SNPs rs3103349–rs10455872 and rs3184504–rs441 was > 250 kb (269,4 kb and 344,2 kb, respectively), SMR/HEIDI analysis was performed for each locus (±250 kb around the lead SNP) separately. The genes prioritized based on literature data and revealed in the gene-based association analysis^49^, if located in two loci in the pair, were attributed to both. Similarly, if the CAD-associated SNPs prioritized in the studies by Brænne *et al*.^46^, Lempiäinen *et al*.^47^, and van der Harst *et al*.^48^ were located in two loci in the pair, we attributed the genes linked with these SNPs to both loci.

For 8 loci (marked by rs4129267, rs10919065, rs12801636, rs3184504, rs441, rs7178051, rs12052058, and rs867186 and numbered as #3, #4, #34, #36, #37, #43, #49, and #50, respectively, in Supplementary Table S1c), the genes were revealed using two or three instrumental variables (“top SNPs”) that were in low or medium LD with each other (Supplementary Table S3a). One or two of the top SNPs in each group were the same as the lead SNP marking the locus or one tightly linked with it (r^2^ = 0.99 in European-ancestry populations according to LDlink). The remaining top SNP in each group was in weak LD with the lead SNP, and association of 5 of them with CAD did not reach a genome-wide level of statistical significance in the dataset used for our SMR/HEIDI analysis (European-ancestry meta-analysis from Howson *et al*. study^31^; locus #4, rs10800418: *P* = 2.42e-07; locus #34, rs644740: *P* = 7.44e-06; locus #37, rs653178: *P* = 1.21e-07; locus #49, rs17616661: *P* = 1.51e-05; locus #50, rs1415771: *P* = 4.70e-06; Supplementary Table S3a). We checked the association of these SNPs with CAD in the meta-analysis of CARDIoGRAMplusC4D and UK Biobank data^48^ (122,733 cases and 424,528 controls). All these SNPs were either genome-wide significant in this dataset or very close to a genome-wide significance level (rs10800418: *P* = 8.82e-11, rs644740: *P* = 1.12e-08, rs653178: *P* = 1.13e-23, rs17616661: *P* = 5.96e-08, rs1415771: *P* = 9.91e-11). Thus, we speculate that the genes *NME7*, *FIBP* and *CTSW*, *SH2B3*, *KANK2*, and *EDEM2* identified using these polymorphisms may not be false positive findings. Nevertheless, the gene *SH2B3* (locus #37, top SNP rs653178) likely came from the locus #36 partially overlapping with the locus #37. In the locus #36, SMR/HEIDI analysis suggested the gene *SH2B3* using the top SNP rs3184504, which is in high LD (r^2^ = 0.95) with rs653178. SNP rs3184504 is the lead SNP in the locus #36 and is associated with СAD with *P* = 3.71e-09 in the European-ancestry meta-analysis from Howson *et al*. study^31^ and with *P* = 1.03e-25 in the CARDIoGRAMplusC4D/UK Biobank meta-analysis^48^.

The gene *IL6R* (locus #3 marked by rs4129267) was indicated in analyses of both semi-independent top SNPs, rs4845625 and rs4129267 (r^2^ = 0.46 in European-ancestry populations according to LDlink). These polymorphisms represent lead SNPs in all-ancestry and European-ancestry meta-analyses reported by Howson *et al*.^31^, respectively, and are associated with CAD with *P*-value less than 5e-10 (Supplementary Table S1a). This suggests at least two independent association signals, both of which modulate *IL6R* expression.

In the locus #25 marked by rs11204085, the top SNP rs1569209 used to identify the gene *LPL* was in weak LD with the lead SNP (Supplementary Table S2a; rs11204085-rs1569209 r^2^ = 0.10 in European-ancestry populations). Rs1569209 was associated with CAD with *P* = 2.29e-06 in the European-ancestry meta-analysis from Howson *et al*. study^31^, however, it reached a genome-wide significant level in the meta-analysis of CARDIoGRAMplusC4D and UK Biobank data^48^ (*P* = 1.81e-09). We therefore do not consider the gene *LPL* found using this polymorphism as a false positive result. Moreover, the role *LPL* in CAD was supported by experimental and *in silico* studies (Supplementary Table S4).

### Cumulative evidence on CAD-associated genes from different studies

The list of genes proposed to be causal for CAD according to different lines of evidence is given in Table 1. Literature overview for each gene suggested by experimental studies is provided in the extended version of this table – Supplementary Table S4.

#### Well-known CAD genes

We analyzed published data and found 18 genes in 18 loci, whose role in CAD and CAD-related processes was strongly supported by experimental studies and/or has already been known before publication of GWAS for CAD. These genes are *PLPP3* (also known as *PAP2B* or *PPAP2B*, locus #1), *SORT1* (locus #2), *IL6R* (locus #3), *APOB* (locus #8), *ABCG8/ABCG5* (locus #9), *GUCY1A3* (locus #15), *PHACTR1* (locus #18), *TCF21* (locus #20), *LPA* (also known as *APOA*, overlapping loci #21 and #22), *LPL* (locus #25), *TRIB1* (locus #26), *CDKN2B-AS1* (*CDKN2B* antisense RNA also known as *ANRIL*, locus #27), *CXCL12* (locus #31), *LIPA* (locus #32), *PDGFD* (locus #35), *ADAMTS7* (locus #43), and *LDLR* (locus #49). The products of these genes are involved in lipid metabolism, inflammation, nitric oxide signaling, cell proliferation and apoptosis, vascular remodeling, and regulation of expression of other CAD-relevant genes.

For 9 out of 18 genes (*IL6R*, *GUCY1A3*, *PHACTR1*, *TCF21*, *LPA*, *LPL*, *LIPA*, *PDGFD*, *ADAMTS7*; 10 loci, *LPA* corresponds to the loci #21 and #22) we also obtained consistent evidence from SMR/HEIDI analysis, indicating that the effects of CAD-associated functional polymorphisms located in the loci containing these genes may be mediated by gene expression. However, data on the expression of *ABCG8* was available only for liver, and we therefore avoid making any conclusions on eQTL effects for this gene. For the remaining well-known CAD genes (*PLPP3*, *SORT1*, *APOB*, *ABCG5*, *TRIB1*, *CDKN2B-AS1*, *CXCL12*, and *LDLR*), our analysis did not support that their expression levels are affected by the same functional variants that are associated with CAD. Several hypotheses can be put forward to explain these results. First, mechanisms other than expression changes may underlie the association between these genes and CAD (i.e., the presence of missense polymorphisms altering the properties of the encoded proteins). Second, CAD-relevant expression changes can occur in tissues/cells, or developmental stages other than those included in our analysis. Third, the absence of statistically significant results in the colocalization analysis does not allow to rule out expression-mediated effects. Genes influencing the trait through expression could be missed due to statistical power limitations/strict statistical significance threshold set in the analyses or due to limitations specific to the input dataset (e.g., incomplete data or possible errors). Besides this, per-SNP sample sizes were not available in the Westra eQTL dataset^36^, and we estimated the eQTL effect sizes from Z-statistics without taking into account per-SNP sample size differences, which could lead to the additional variation in the effect size estimates^27^. Finally, in case of multiple association signals, the HEIDI test may erroneously reject the null hypothesis and disregard the results on the genes whose expression is actually related to the disease. In an extreme scenario where the two causal variants (e.g., affecting CAD and gene expression) are in perfect LD, pleiotropy and linkage disequilibrium are indistinguishable by any statistical test^27^. Thus, it is possible that our colocalization analysis could miss some CAD-relevant genes.

Fifteen out of 18 well-known CAD genes (all except *ABCG5*, *TRIB1* and *CXCL12*) were also prioritized in at least one of the four previously published *in silico* studies^46–49^. Thus, only for three genes evidence for their role in CAD came only from experimental works. It is noteworthy that among the remaining well-known CAD genes identified in both experimental and *in silico* (our and/or other) studies, only the genes *PLPP3*, *APOB*, *GUCY1A3*, and *LPL* were proposed as single candidates. For *ABCG8/ABCG5*, bioinformatic studies prioritized only *ABCG8*, while literature data support CAD-related effects of both (products of these genes have closely related function: they form heterodimer that limits intestinal absorption and facilitates biliary secretion of cholesterol)^50,51^. For other loci, bioinformatic studies prioritized from 2 to 7 genes (median = 5). We presented all these genes in Table 1 and Supplementary Table S4 regardless of scores given to them in studies^46,47^, LD between a lead SNP marking a locus and SNPs that were used to prioritize these genes in studies^46–48^ (data on LD are given in Supplementary Table S3b), and LD between lead SNPs and “top SNPs” from SMR/HEIDI analysis (data on LD are given in Supplementary Table S2a).

We suppose that many of the multiple genes that were simultaneously prioritized in the same loci are not specific for CAD. For instance, the genes *IFIT1* and *IFIT5* encoding interferon-induced antiviral RNA-binding proteins, which were revealed in SMR/HEIDI along with *LIPA* (locus #32), may be not causal for CAD. It is possible that the locus #32 contains a regulatory polymorphism (or polymorphisms in very strong LD), which alters the expression of both *LIPA* and *IFIT1/IFIT5*. Its causal effect on CAD can be explained by modulation of *LIPA* expression, while effects on *IFIT1/IFIT5* expression seem to be pleiotropy.

However, filtering out all of these “unspecific” genes may be too strict approach. It is not necessary that a single causal gene explains association between a locus and CAD. In fact, each locus can contain more than one independent association signal, and each association signal can realize its effect via more than one causal gene (as well as each causal gene can be affected by more than one functional CAD-associated polymorphism). In our opinion, loci for which multiple studies prioritized the same additional genes deserve special attention. The examples are locus #2, locus #49 and overlapping loci #21 and #22 (Table 1, Supplementary Table S4). We suppose that besides undoubtedly causal genes *LDLR* and *LPA*, relevance for CAD is likely for the genes *SLC22A3, SLC22A2, SLC22A1* (encoding organic cation transporters), *PLG* (encoding plasminogen involved in hemostasis), *SMARCA4* (encoding a protein involved in vascular calcification^52^), and *CARM1* (encoding methyltransferase involved in the control of stress-induced lipid metabolism^53^). In the locus #2, almost all *in silico* and gene expression studies prioritized *CELSR2* and *PSRC1* along with the *SORT1* gene. Moreover, *PSRC1* was shown to protect against atherosclerosis and enhance the stability of atherosclerotic plaques in *Apoe*^*-/-*^ mice by modulating cholesterol transportation and inflammation^54^. Thus, *CELSR2* and *PSRC1* in the locus #2 might be also involved in CAD development.

Other interesting examples of multiple candidate genes in a locus are the genes of long noncoding RNA (lncRNA) prioritized in experimental or *in silico* studies (loci #18, #20, #27, and #35). LncRNA CDKN2B-AS1 (ANRIL; locus #27) regulates the expression of *CDKN2A/B* and other genes and has well-known effects on atherosclerosis^55–57^. We suppose that lncRNA RP3-323P13.2 (also known as TARID; locus #20) indicated by our SMR/HEIDI analysis can in the same way be relevant for CAD via the regulation of expression of CAD-associated gene *TCF21*. In the study by Arab *et al*.^58^, TARID was shown to activate *TCF21* expression via interaction with *TCF21* promoter as well as with the regulator of DNA demethylation GADD45A. In the loci #18 and #35, SMR/HEIDI analysis suggested lncRNAs RP1-257A7.4 and RP1-257A7.5 (the first is antisense to *PHACTR1* and the gene encoding the second one is located near *PHACTR1*) and RP11-563P16.1 (its gene is located 12 kb from *PDGFD*). However, we did not find any evidence in published studies that these lncRNAs can regulate *PHACTR1* and *PDGFD* transcription and therefore do not consider them as a likely causal CAD genes.

#### Other causal/the most likely causal CAD genes

We found additional 37 genes in 27 loci, whose role in CAD and CAD-related processes can be proposed based on evidence from published “wet” experimental studies (Table 1, Supplementary Table S4). We considered this evidence not strong enough to prioritize any of these genes convincingly based on experimental data alone. However, adding data from *in silico* studies allowed us to pinpoint 9 causal and 10 most likely causal CAD genes in 8 and 10 loci, respectively.

The genes that we consider as definitely causal for CAD are *MRAS* (locus #13), *EDNRA* (also known as *ETA*, locus #14), *JCAD* (also known as *KIAA1462*, locus #29), *SCARB1* (locus #39), *FLT1* (also known as *VEGFR1*, locus #40), *COL4A2*/*COL4A1* (locus #41), *FURIN* (locus #44), and *PECAM1* (locus #48). The genes that we define as “the most likely causal” are *ATP1B1* (locus #4), *ZC3HC1* (also known as *NIPA*, locus #23), *TBXAS1* (locus #24), *CYP17A1* (locus #33), *SH2B3* (also known as *LNK*, locus #36), *HNF1A* (locus #38), *HHIPL1* (locus #42), *HP* (locus #45), *PROCR* (locus #50), and *KCNE2* (also known as *MIRP1*, locus #51). Of those, *MRAS*, *JCAD*, *FURIN*, *PECAM1*, *ATP1B1*, *SH2B3*, *HP*, and *KCNE2* were found in our SMR/HEIDI analysis, supporting expression-related effects on CAD.

Only for three loci (#14, #29 and #40) the genes *EDNRA*, *JCAD*, and *FLT1* were proposed as single possible candidates in all studies. For other loci, from 2 to 14 genes were proposed as potentially causal (median = 4). The largest number of genes was suggested for the locus #50 (n = 14), and almost all of these genes were prioritized based on the same putative functional SNP rs867186 as that prioritized with the most likely causal gene *PROCR* (Table 1, Supplementary Table S3a,b). Thus, we cannot explain such diversity by the presence of multiple association signals in this locus and consider additional genes as likely unspecific results.

Among the remaining loci with multiple proposed candidates, in our opinion, special attention should be paid to the loci #23, #36, and #44. In the locus #23, we found the strongest evidence for the gene *ZC3HC1* (Supplementary Table S4). *ZC3HC1* contains a functional missense polymorphism rs11556924^59^, which is the lead SNP tagging this locus. However, our SMR/HEIDI analysis revealed that either rs11556924 or other SNP in LD with rs11556924 is simultaneously associated with CAD and the *KLHDC10* gene expression in blood (Supplementary Table S2). The product of *KLHDC10* is involved in oxidative stress-induced cell death and inflammation^60,61^. Since all these processes are playing role in atherosclerosis^62–64^, we suppose that changes in *KLHDC10* expression can be an additional factor explaining association between locus #23 and CAD. In the locus #36, lead SNP rs3184504 is a missense polymorphism in the *SH2B3* gene. Interestingly, rs3184504 was also a “top SNP” for *SH2B3* in our SMR/HEIDI analysis that indicated this gene (Supplementary Table S2). This may mean that either effect of rs3184504 on CAD is realized not/not only via altering the SH2B3 protein properties (for example, it can influence *SH2B3* transcription or mediate RNA decay), or the locus #36 contains two functional CAD-associated SNPs in LD with each other – a missense SNP rs3184504 and another SNP affecting *SH2B3* expression. Besides *SH2B3* suggested by many studies, three lines of evidence support the role of *ATXN2* (Table 1, Supplementary Table S4), including the results of the study on ataxin-2 knock-out mice (such animals displayed different pathological changes such as obesity and increased serum cholesterol level^65^). Thus, we do not exclude causality for *ATXN2*. Finally, in the locus #44, all *in silico* studies prioritized both *FURIN* and *FES* genes. Our SMR/HEIDI analysis found association between CAD and *FURIN* expression changes in blood, and between CAD and *FES* expression changes in blood and CD14 + and CD19 + cells. Notably, Liu *et al*.^66^ have recently applied colocalization methods on the transcriptome dataset generated using human coronary artery smooth muscle cell lines collected from donor hearts. They observed colocalization between CAD and gene expression association signals in this locus only for *FES* (the genes found in that study for other loci were *TCF21*, *SIPA1*, *PDGFRA*, and *SMAD3*, with the first two also supported by our SMR/HEIDI results and the last two coming from loci not analyzed in this study). Nevertheless, in the present study, we prioritized *FURIN* since only for this gene experimental data support CAD-related role of its protein product (Supplementary Table S4).

#### Loci with inconclusive evidence

For the 15 remaining loci, we could not suggest any causal gene due to inconsistency in the results of different studies or insufficient data for gene prioritization.

For the loci #7 and #30, no candidate genes were found, and for the loci #16, #17, and #19, evidence was not enough to make any conclusion. In the loci #5, #6, #10-12, #28, #34, #37, #46, and #47, the studies suggested multiple genes (from 2 to 10, median = 4). We failed to prioritize any and presented all of them in Table 1 and Supplementary Table S4 without inferences of causality. It is worth pointing out that in the locus #47, we could not choose between three strong candidates *PEMT, SREBF1*, and *MIR33B*, all of which can be – based on experimental studies – judged as relevant for CAD. Besides this, we want to point out the locus #28, for which experimental studies (Supplementary Table S4) and the gene-based analysis^49^ proposed the candidate genes *ABO* and *ADAMTS13*. Our SMR/HEIDI analysis supported the role of *ABO*. For the locus #10, experimental evidence suggested the genes *GGCX* and *VAMP8*, which were prioritized in almost all *in silico* studies along with *VAMP5* and some other candidates. Whether one or more of these genes are causal for CAD remains in question.

## Discussion

**Figure 1:**
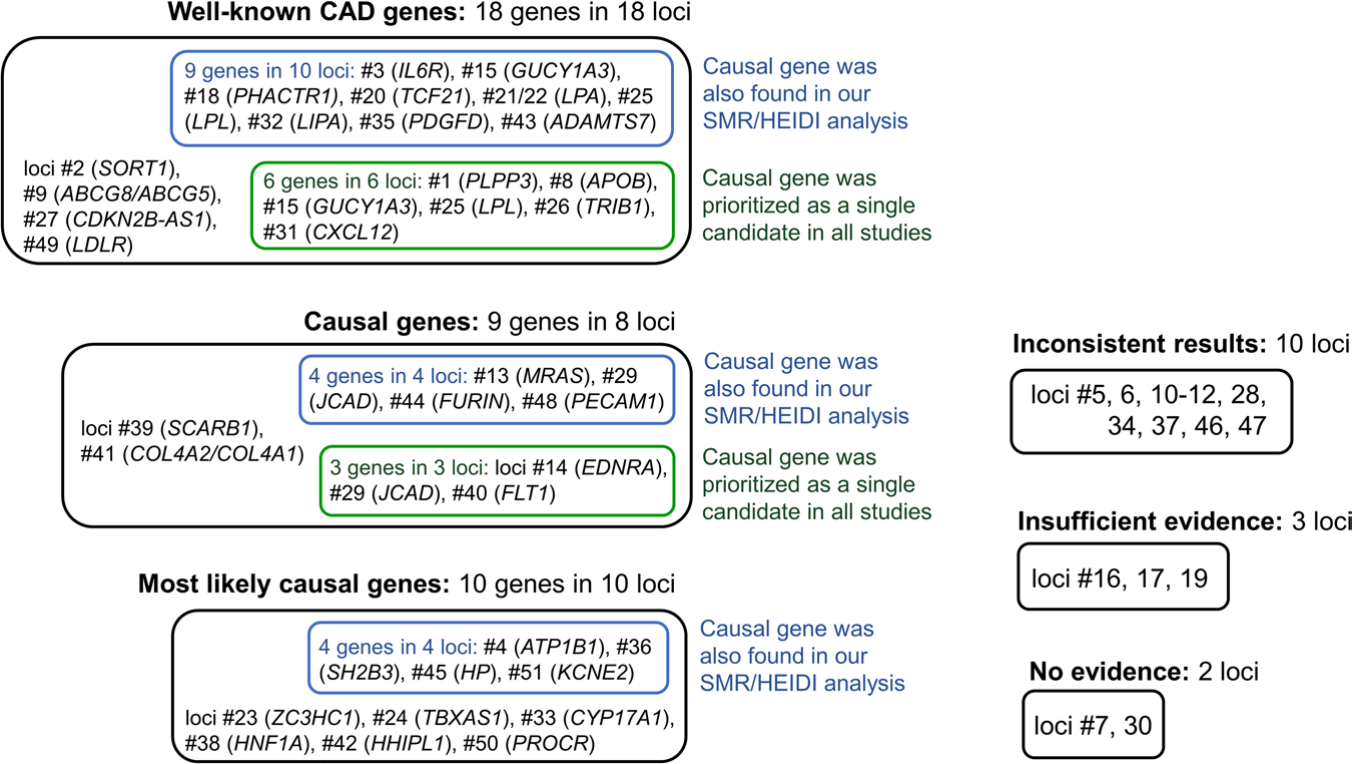
Summary of findings for 51 CAD-associated loci. Matching loci numbers with chromosomal positions and lead SNPs can be found in Table 1 and Supplementary Table S1c. Prioritized genes are listed in Table 1 and Supplementary Table S4.

Genome-wide association studies offer great opportunities for exploring genetic architecture of complex traits due to their whole-genome scale and hypothesis-free design. However, annotation of GWAS results is usually not straightforward and requires extensive *in silico* research and experimental follow-up. In the present study, we aimed to pinpoint the genes that account for associations between 51 genomic loci and CAD. We also aimed to reveal the loci for which evidence on CAD-associated genes remains insufficient or controversial. We collected and systematized data from published studies and complemented their results with the results of our bioinformatics analysis of colocalization between GWAS signals and eQTLs using SMR/HEIDI approach^27^. Our results, information from other works and overall conclusions are summarized in Table 1; even more detailed summary with a literature review of experimental findings is presented in Supplementary Table S4. Overview of all findings is provided in Fig. 1.

Using merely *in silico* techniques and previous literature, we conclude that for 36 out of 51 (71%) CAD-associated loci, the causal/most likely causal genes have been identified. For 18 genes in 18 loci, we found that very strong previous experimental evidence supports their relevance for CAD and defined them as “well-known CAD genes”. This role for 15 of them is also supported by bioinformatics studies^46–49^. Our SMR/HEIDI analysis confirmed the role of 9 of these 18 genes (*IL6R*, *GUCY1A3*, *PHACTR1*, *TCF21*, *LPA*, *LPL*, *LIPA*, *PDGFD*, *ADAMTS7*), indicating that the same causal SNPs are associated with CAD and gene expression changes in CAD-relevant tissues. Furthermore, we made causal inferences for 19 genes in 18 other loci based on cumulative evidence from *in silico* and experimental works. Eight of them (*JCAD*, *FURIN*, *PECAM1*, *ATP1B1*, *SH2B3*, *HHIPL1*, *HP*, and *KCNE2*) were found in our SMR/HEIDI analysis, supporting expression-mediated mechanisms underlying CAD-loci associations.

We could not make causal inference for 15 (29%) loci. We found out that for 5 loci, evidence for CAD-associated genes remains insufficient or absent. For the remaining 10 loci, we observed a considerable inconsistency in the results obtained using different approaches and/or could not choose from multiple genes for which strength of evidence supporting their role was similar. Thus, we conclude that for these 15 loci, it would be beneficial to conduct additional studies clarifying the causal gene.

It should be noted that our and other studies suggested more than one candidate gene per locus for 37 out of 51 (73%) analyzed loci (including 12 loci with well-known CAD genes). There may be several explanations for such multiplicity. First of all, *in silico* methods may produce unspecific results. For instance, colocalization between gene expression and CAD association signals does not prove causality – this method only provides possible candidate genes whose transcription is affected by the same SNP that influences the risk of CAD, and the results of different colocalization methods may have a low concordance with each other^67^. In bioinformatics studies of Brænne *et al*.^46^ and Lempiäinen *et al*.^47^ that used different prioritization algorithms and *in silico* methods, the “nonspecificity” issue was addressed by providing scores to the revealed genes. Nevertheless, as can be seen from Table 1, a high score in one study does not necessarily correlate with a high score in another. We estimated a correlation between the scores assigned to the genes prioritized in both Brænne *et al*.^46^ and Lempiäinen *et al*.^47^ studies (only the genes attributed to 51 loci studied in our work were included in the analysis). The Spearman’s correlation coefficient was *ρ* = 0.204. When we considered only the genes in these loci prioritized with the same SNP (or with SNPs in high LD with each other, r^2^ ≥ 0.8), the Spearman’s correlation coefficient was *ρ* = 0.290.

Second, in our study, “a CAD-associated locus” was defined as a physical distance of ±250 kb around the lead SNP (showing the strongest association in GWAS), and we did not focus on independent association signals. In the case of multiple neighboring SNPs independently associated with the disease, each one can realize its effect via its own causal gene. Besides this, theoretically, one functional SNP (e.g., regulatory) can affect more than one disease-relevant gene. Thus, it is not surprising that out colocalization analysis and analyses performed in other studies often suggested many genes per locus. Here we presented all information on CAD-associated genes suggested by our SMR/HEIDI tests and thoroughly extracted from different studies irrespective of scores given to these genes (if any) and LD between SNPs, through which the genes were prioritized, and the lead GWAS SNPs (data on LD can be found in Tables S2 and S3). Furthermore, our study emphasized the loci where multiple causal genes are likely (e.g. *TCF21* and *RP3-323P13.2* in locus #20; *ZC3HC* and *KLHDC10* in locus #23, *SH2B3* and *ATXN2* in locus #36 etc., see Table 1). In our opinion, such loci should receive special attention in subsequent research.

Our study has strengths and limitations. A principal strength of our study is a systematic and comprehensive approach to data extraction and reporting. Each locus was analyzed individually taking into accordance all available information. However, we acknowledge that manual annotation may lead to some degree of subjectivity in making decisions, and we therefore made as much data as possible available for independent scrutiny. Another limitation is that we analyzed only 51 CAD-associated loci discovered until 2017 and for which we were able to perform a SMR/HEIDI analysis, while more than 160 CAD loci are known to date^9,15^. Expanding our analyses to include all of them would be beneficial, although for some recently discovered loci there may still be too few literature data on candidate genes to draw a conclusion on their relevance for CAD in the context of our work. Next, our study had limitations related to the use of colocalization analysis, which, on the one hand, may miss some important CAD-associated genes due to limited power/incomplete data/multiple association signals in regions with complex LD structure (the last being an inherent problem of the HEIDI test), and, on the other hand, may suggest genes which are actually not related to CAD. In particular, it should be noted that for some loci the number of SNPs in the HEIDI test was quite small (Supplementary Table S2a), which could lead to limited power to detect heterogeneity and increase the probability that the expression of identified genes is associated with functional variants other than those affecting CAD. Finally, we did not provide deep insights into the mechanisms linking genomic variations in the studied loci with alterations in gene functions. Nevertheless, we showed that for 17 causal/most likely causal genes, this mechanism may be related to changes in gene expression in CAD-relevant tissues.

Considering issues related to the consistency between the results of colocalization methods^67^ and concerns that the HEIDI test might be too conservative^27^, we applied alternative colocalization methods using a theta metric-based approach suggested by Momozawa *et al*.^29^ and the LocusCompare^68^ web tool (http://locuscompare.com/). The theta metric-based analysis assesses the similarity between association patterns and provides an alternative to the HEIDI test. We used the same sources of GWAS summary statistics as in the SMR/HEIDI test and applied the threshold of |*θ* | > 0.7 and the number of SNPs >3. The theta-metric based analysis proposed 39 genes related to 19 loci (Supplementary Table S5), of which 32 genes were also identified in our SMR/HEIDI analysis, while 9 genes (*A4GNT*, *AS3MT*, *IREB2*, *MAT2A*, *SH3PXD2A*, *SLC3A1*, *SORT1*, *SRR*, *WDR12*) were not. Of the 9 genes listed above, *AS3MT* (locus #33), *MAT2A* (locus #10), *SORT1* (locus #2), *SRR* (locus #46), and *WDR12* (locus #12) were also proposed by some previous studies (Table 1, Supplementary Table S4), and the genes *A4GNT* (locus #13), *IREB2* (locus #43), *SH3PXD2A* (locus #33), and *SLC3A1* (locus #9), to the best of our knowledge, have never been suggested for CAD before. It is worth noting that for these novel genes, evidence for expression-mediated effects was found only for one tissue per each gene. Next, we used the LocusCompare web framework with the Howson *et al*.^31^ CAD GWAS and CAD-relevant tissues^37^ from the GTEx version 7 eQTL dataset. Using the recommended threshold for probability of > 0.01, we identified 24 genes related to 16 loci (Supplementary Table S6), including 23 genes overlapping with our SMR/HEIDI results, and the gene *HHIPL1* reported in other works (Supplementary Table S4). *HHIPL1* passed the FDR threshold in our SMR test for two tissues (Supplementary Table S2b) but was omitted from the HEIDI test due to the insufficient number of SNPs in the analysis. Overall, having compared the new results with the evidence summarized using SMR/HEIDI and published studies, we conclude that the results of theta metric-based and LocusCompare analyses do not change the decisions on the prioritized genes made in the present study.

Despite limitations, our study contributes to a better understanding of the genetic underpinnings of CAD by supporting the results of previous annotation efforts, resolving some uncertainty issues by consolidating data from different sources, and outlining new research directions by suggesting novel CAD candidate genes. In addition, our study pinpoints the loci for which causal genes remain unknown and evidence is still ambiguous or inconclusive, highlighting the need for further research to address these knowledge gaps.

## Conclusion

In the present study, we prioritized the genes responsible for the association of 51 loci with CAD based on cumulative evidence from experimental and *in silico* studies, including our SMR/HEIDI analysis of colocalization between eQTL and GWAS signals. We identified causal/most likely causal gene for 36 (71%) loci. For 10 loci, we concluded that evidence for gene prioritization is inconsistent. For 5 loci, data remain insufficient or absent. We envisage that data collected and summarized here will provide useful guidance for future studies.

## Supplementary information

Supplementary Figures.

Supplementary Methods.

Supplementary Table S1.

Supplementary Table S2.

Supplementary Table S3.

Supplementary Table S4.

Supplementary Table S5.

Supplementary Table S6.

Supplementary Data Legends.

## Data Availability

Data obtained in the analyses are provided in Supplementary Tables related to this article.
