# Supplementary material for: Prioritization of causal genes for coronary artery disease based on cumulative evidence from experimental and in silico studies
Source: Sci Rep. 2020 Jun 26;10:10486. doi: 10.1038/s41598-020-67001-w (PMC7320185; doi:10.1038/s41598-020-67001-w)

Prioritization of causal genes for coronary artery disease based on cumulative evidence from experimental and *in silico* studies

Alexandra S. Shadrina, Tatiana I. Shashkova, Anna A. Torgasheva, Sodbo Z. Sharapov, Lucija Klarić, Eugene D. Pakhomov, Dmitry G. Alexeev, James F. Wilson, Yakov A. Tsepilov, Peter K. Joshi, Yurii S. Aulchenko

Supplementary Figures

Supplementary Figure S1. A scheme depicting selection of CAD-associated loci for SMR/HEIDI analysis.
Supplementary Figure S2. A pipeline of extracting data from the previous studies on the genes potentially associated with CAD.

Supplementary Figure S1. A scheme depicting selection of CAD-associated loci for SMR/HEIDI analysis.

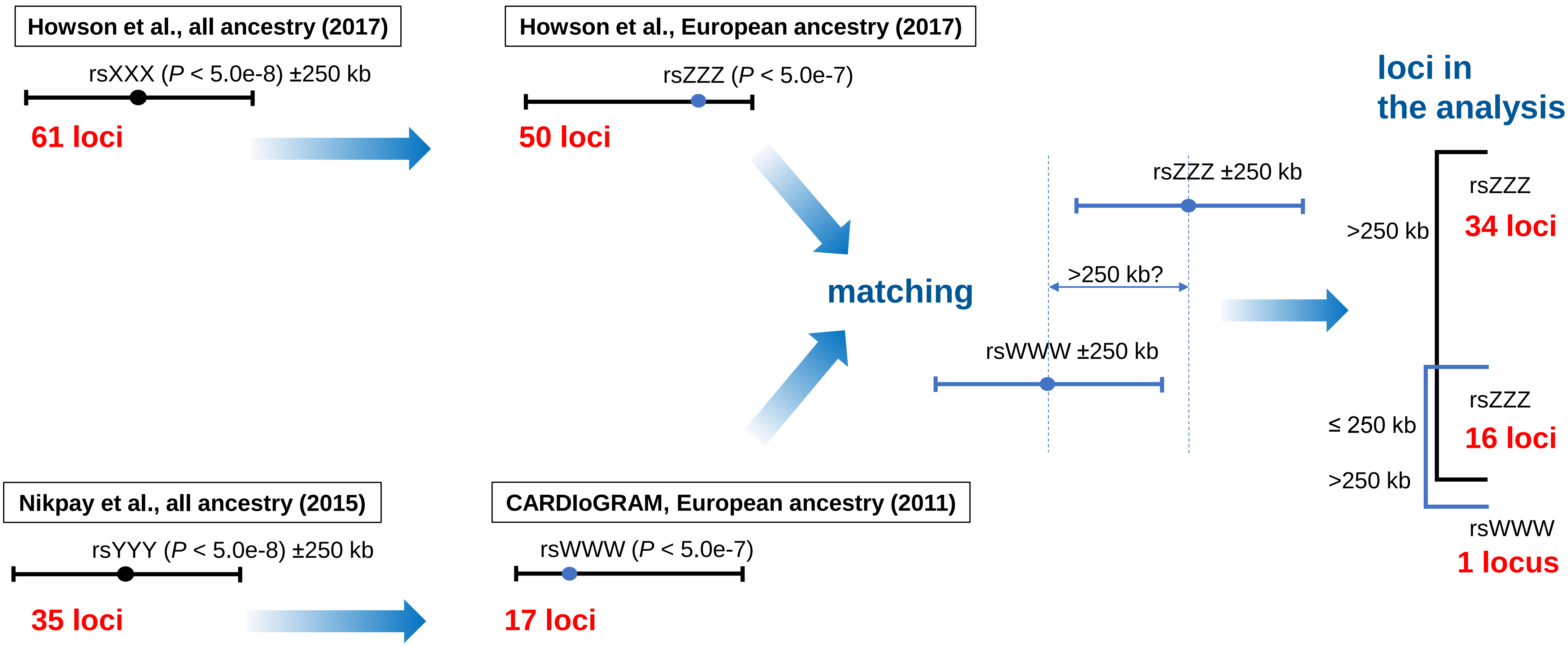

Supplementary Figure S2. A pipeline of extracting data from the previous studies on the genes potentially associated with CAD.

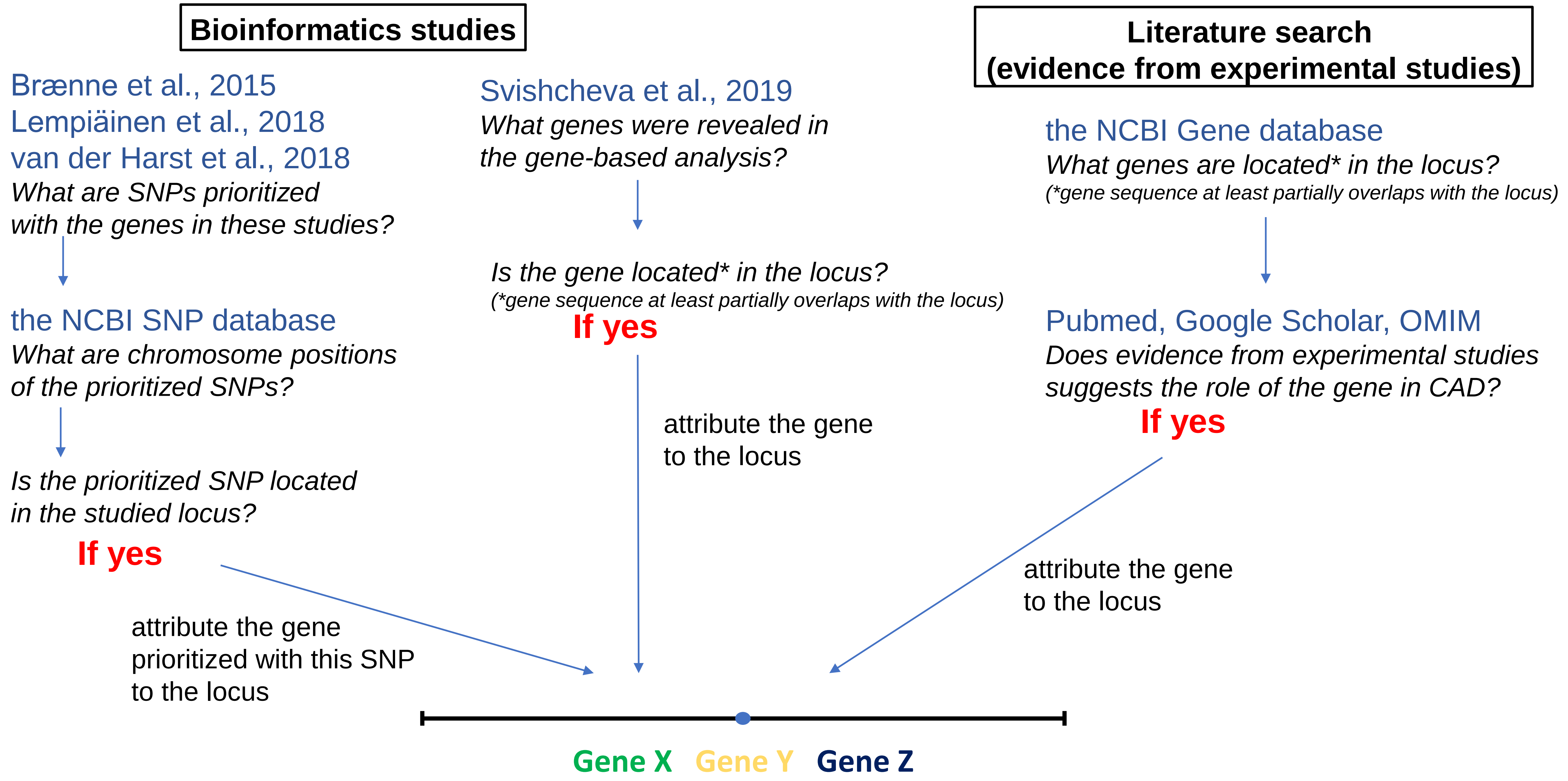

Supplement: Supplementary file 1 — Supplementary Figures. [file 41598_2020_67001_MOESM1_ESM.pdf]
