# Supplementary material for: Prioritization of causal genes for coronary artery disease based on cumulative evidence from experimental and in silico studies
Source: Sci Rep. 2020 Jun 26;10:10486. doi: 10.1038/s41598-020-67001-w (PMC7320185; doi:10.1038/s41598-020-67001-w)
Supplement: Supplementary file 2 — Supplementary Methods. [file 41598_2020_67001_MOESM2_ESM.pdf]

Supplementary Methods

SMR/HEIDI analysis

SMR/HEIDI analysis was conducted as described by Zhu et al.[1] HEIDI statistics was calculated as $T_{HEIDI} = \sum_i^m z_{d(i)}^2$, where m is the number of SNPs selected for analysis, $z_{d(i)} = {d_i}/{SE_{(d_i)}}$ and $d_i = \beta_{SMR_i} - \beta_{SMR\ (lead\ SNP)}$.

SNP selection was performed as follows:

1) We defined a set of eligible markers within ±250 kb from the lead SNP in GWAS for CAD, which (i) were present in both GWAS for CAD and in the eQTL data; (ii) had MAF ≥ 0.03 in both datasets; (iii) had squared Z-test value ≥ 10 in GWAS for CAD;
2) Made empty “target” and “rejected” SNP sets;
3) Selected SNP from the primary GWAS with the lowest *P* (“top SNP”) and added it into the “target” SNP set;
4) If the SNP had $r^2 > 0.9$ with any SNP in the “target” SNP set, we added it to the “rejected” set.
   LD matrix (r^2) was computed with PLINK 1.9 (https://www.cog-genomics.org/plink2) using 1000 Genomes data for 503 European ancestry individuals (http://www.internationalgenome.org/data/);
5) Otherwise, it was added to the “target” set;
6) Procedure was repeated from the step 3) until either eligible SNP set was exhausted, or the “target” set had 20 SNPs. If we could not select 3 or more SNPs, no test was performed (the default number of SNPs was used as indicated in the manual https://cnsgenomics.com/software/smr/#SMR&HEIDIanalysis, accessed on 8 May 2020).

When testing for pleiotropy with complex traits, we standardized all SNP effects (β) and standard errors (made the variances of the traits equal to 1): $\breve{\beta}_{Y_i} = \beta_{Y_i}/SD_{Y_i}$ and $\breve{SE}_{Y_i} = SE_{Y_i}/SD_{Y_i}$, where $\breve{\beta}_{Y_i}$ and $\breve{SE}_{Y_i}$ are standardized betas and standard errors for the trait Y_i; β_{Y_i} and SE_{Y_i} are original betas and standard errors for the trait Y_i; SD_{Y_i} is a square root of estimated variance of the trait Y_i. Analysis was conducted using Python 3.5 as the main programming language.

REFERENCES:

1. Zhu, Z. et al. Integration of summary data from GWAS and eQTL studies predicts complex trait gene targets. *Nat. Genet.* **48**, 481–487 (2016). doi:10.1038/ng.3538

GWAS-MAP platform

GWAS-MAP platform integrates a database of summary-level GWAS results for 2,586 complex traits, gene expression levels in different tissues and cell types (eQTL data), and an embedded software for LD Score regression[1], two-sample Mendelian randomization analysis (the MR-Base package)[2,3], and our implementation of SMR/HEIDI analysis[4].

SNPs from each GWAS were matched with polymorphisms from 1000 Genomes Project Phase 3 version 5 reference panel (http://www.internationalgenome.org/). SNPs with conflicting data on rsid, position, and alleles were excluded. For SNPs that have passed this filtering, alleles were harmonized across all GWAS and sorted in a lexicographic order.

GWAS summary statistics is stored using the ClickHouse database management system (https://clickhouse.yandex/). For each trait, we created an annotation file that contains information about the study design and key characteristics of association analysis (name of the cohort, sample size, model of inheritance, trait transformations, reference population, etc.). Metadata is organized with the PostgreSQL database system (https://www.postgresql.org/).

Data included in the GWAS-MAP database.

<table>
<tr><th>Dataset/source</th><th>Number of traits</th><th colspan="2">Description</th><th>Reference</th></tr>
<tr><td>The Neale Lab</td><td>2,419</td><td colspan="2">Complex traits from the UK Biobank*</td><td>http://www.nealelab.is/</td></tr>
<tr><td>The Gene ATLAS</td><td>34</td><td colspan="2">Complex traits from the UK Biobank**</td><td>http://geneatlas.roslin.ed.ac.uk/</td></tr>
<tr><td>“Metabolomics_NMR”</td><td>123</td><td colspan="2">Circulating metabolites quantified with the NMR metabolomics platform (University Hospitals of Strasbourg, France)</td><td>[5]</td></tr>
<tr><td rowspan="10">“CAD_traits”</td><td rowspan="10">10</td><td rowspan="10">Coronary artery disease-related traits</td><td>Coronary artery disease</td><td>[6]</td></tr>
<tr><td>Coronary artery disease</td><td>[7]</td></tr>
<tr><td>Cigarettes smoked per day</td><td>[8]</td></tr>
<tr><td>Body mass index</td><td>[9]</td></tr>
<tr><td>Waist-to-hip ratio</td><td>[10]</td></tr>
<tr><td>Educational attainment</td><td>[11]</td></tr>
<tr><td>Total cholesterol</td><td>[12]</td></tr>
<tr><td>Triglicerides</td><td>[12]</td></tr>
<tr><td>Low-density lipoproteins</td><td>[12]</td></tr>
<tr><td>High-density lipoproteins</td><td>[12]</td></tr>
<tr><td rowspan="3">“eQTL_data”</td><td rowspan="3">-</td><td rowspan="3">Gene expression levels in different tissues and cell types</td><td>Coronary and tibial artery, aorta, liver, skeletal muscle, whole blood (from GTEx version 6 database)</td><td>[13]</td></tr>
<tr><td>Circulating CD4+ T lymphocytes, CD8+ T lymphocytes, CD19+ B lymphocytes, CD14+ monocytes, CD15+ granulocytes, and platelets (from CEDAR project)</td><td>[14]</td></tr>
<tr><td>Peripheral blood (from Westra Blood eQTL study)</td><td>[15]</td></tr>
</table>

*Data from the Neale Lab database were downloaded on December 15, 2017.
**Data from the Gene ATLAS were downloaded on December 8, 2017.

REFERENCES:

1. Bulik-Sullivan, B. K. *et al.* LD Score regression distinguishes confounding from polygenicity in genome-wide association studies. *Nat. Genet.* **47,** 291–295 (2015). doi:10.1038/ng.3211
2. Hemani, G. *et al.* MR-Base: a platform for systematic causal inference across the phenome using billions of genetic associations. *bioRxiv* **078972** (2016). doi:10.1101/078972
3. Hemani, G. *et al.* The MR-Base platform supports systematic causal inference across the human phenome. *Elife* **7,** e34408 (2018). doi:10.7554/eLife.34408
4. Zhu, Z. et al. Integration of summary data from GWAS and eQTL studies predicts complex trait gene targets. *Nat. Genet.* **48**, 481–487 (2016). doi:10.1038/ng.3538
5. Kettunen, J. *et al.* Genome-wide study for circulating metabolites identifies 62 loci and reveals novel systemic effects of LPA. *Nat. Commun.* **7,** 11122 (2016). doi:10.1038/ncomms11122
6. Schunkert, H. et al. Large-scale association analysis identifies 13 new susceptibility loci for coronary artery disease. *Nat. Genet.* **43**, 333–338 (2011). doi:10.1038/ng.784
7. Howson, J. M. M. *et al.* Fifteen new risk loci for coronary artery disease highlight arterial-wall-specific mechanisms. *Nat. Genet.* **49,** 1113–1119 (2017). doi:10.1038/ng.3874
8. The Tobacco and Genetics Consortium. Genome-wide meta-analyses identify multiple loci associated with smoking behavior. *Nat. Genet.* **42,** 441–447 (2010). doi:10.1038/ng.571
9. Locke, A. E. *et al.* Genetic studies of body mass index yield new insights for obesity biology. *Nature* **518,** 197–206 (2015). doi:10.1038/nature1417
10. Shungin, D. *et al.* New genetic loci link adipose and insulin biology to body fat distribution. *Nature* **518,** 187–196 (2015). doi:10.1038/nature14132
11. Okbay, A. *et al.* Genome-wide association study identifies 74 loci associated with educational attainment. *Nature* **533,** 539–542 (2016). doi:10.1038/nature17671
12. Willer, C. J. *et al*. Discovery and refinement of loci associated with lipid levels. *Nat. Genet.* **45**, 1274–1283 (2013). doi:10.1038/ng.2797
13. GTEx Consortium. The Genotype-Tissue Expression (GTEx) project. *Nat. Genet.* **45**, 580–585 (2013). doi:10.1038/ng.2653
14. Momozawa, Y. et al. IBD risk loci are enriched in multigenic regulatory modules encompassing putative causative genes. *Nat. Commun.* **9**, 2427 (2018). doi:10.1038/s41467-018-04365-8
15. Westra, H.-J. et al. Systematic identification of trans eQTLs as putative drivers of known disease associations. *Nat. Genet.* **45**, 1238–1243 (2013). doi:10.1038/ng.2756

Approaches for gene prioritization used in previous studies (a brief overview)

Brænne, I. et al. Prediction of causal candidate genes in coronary artery disease loci. *Arterioscler. Thromb. Vasc. Biol.* 35, 2207–2217 (2015). doi:10.1161/ATVBAHA.115.306108

Potential causal CAD-associated SNPs (lead GWAS SNPs and SNPs in high LD with them) were selected based on any of the following criteria: (1) deleterious effects on amino acid sequence, (2) eQTL effect, (3) possible effects on transcription factor binding due to their presence in regulatory regions. Genes functionally related to these SNPs were scored and prioritized based on prior knowledge- or data-driven approaches.

1. Prior-knowledge-driven prioritization (score +1 per each point):
- PubMed article abstracts (GRAIL)
- "Guilt-by-association" (DEPICT)
- Mouse Phenotype (MGD)
- Disease Ontology (FunDO)
- Biochemical Pathways (ConsensusPathDB)
- Gene Ontology (AmiGO)

2. Data-driven prioritization (score +1 per each point):
- Risk SNP has an eQTL effect
- Risk SNP has a protein altering effect
- Risk SNP in promoter region
- Key driver (1st neighbour) (Bayesian networks)
- Clinical/intermediate human/mouse phenotype (SGR)

Lempiäinen, H. et al. Network analysis of coronary artery disease risk genes elucidates disease mechanisms and druggable targets. *Sci. Rep.* 8, 3434 (2018). doi:10.1038/s41598-018-20721-6

First, genes were linked to CAD-associated loci (genome-wide significant and suggestive CAD SNPs) based on any of the following evidence:
- Proximity to lead and high LD SNPs ascertained by searching the ENSEMBL database (GRCh38) for RefSeq annotated genes
- A long-range interaction between a chromosomal region containing a gene and the region containing the CAD-associated SNPs
- The eQTL most significantly associated with the CAD lead SNP or proxy.

Second, genes were scored and prioritized using six criteria for functional relations:
- The SNP is transcribed

- The SNP has a chromatin interaction with the gene
- The SNP causes a coding change
- The SNP is an eQTL
- The SNP is predicted to be deleterious
- The mouse knockout of the gene has an atherosclerosis phenotype

Each category was assigned a weight of 2^(*L*-1), where *L* is the rank of the category. The score for each gene was the sum of the weighted score across the six categories. The top-scoring gene was considered the most likely to be causal.

van der Harst, P. & Verweij, N. Identification of 64 Novel Genetic Loci Provides an Expanded View on the Genetic Architecture of Coronary Artery Disease. *Circ. Res.* 122, 433–443 (2018). doi:10.1161/CIRCRESAHA.117.312086

Causal CAD-associated variants were prioritized via the probabilistic framework of Probabilistic Annotation Integrator. Genes were linked to these variants based on the following lines of evidence:

- Gene with a missense causal variant
- Causal variant is also significantly associated with gene expression (eQTL analysis)
- Chromatin interaction between the causal variant and the promotor of the gene
- Gene of which the 3′ untranslated region overlaps with the causal variant

For some genes, converging evidence of a potential functional SNP-gene mechanism was demonstrated, providing the strongest basis for prioritization.

Svishcheva, G. R., Belonogova, N. M., Zorkoltseva, I. V, Kirichenko, A. V & Axenovich, T. I. Gene-based association tests using GWAS summary statistics. *Bioinformatics* btz172, (2019). doi:10.1093/bioinformatics/btz172

Gene-based association tests were performed using GWAS summary statistics obtained from two resources:

- Myocardial Infarction Genetics and CARDIoGRAM Exome meta-analysis (http://www.cardiogramplusc4d.org/)
- UK Biobank (summary-level data provided by the Neale Lab, http://www.nealelab.is/)

The following methods of gene-based association analysis were used: BT, SKAT, SKAT-O, MLR, FLM and PCA methods (implemented in sumFREGAT package), the method of minimum *P*-value (implemented in PASCAL and SimpleM of COMBAT), and the sum of χ^2-statistics method (implemented in PASCAL and fastBAT packages). All these methods were modified to utilize GWAS summary statistics as input.

Genes were selected demonstrating significant associations with CAD by at least one method.
