# Supplementary material for: Prioritization of causal genes for coronary artery disease based on cumulative evidence from experimental and in silico studies
Source: Sci Rep. 2020 Jun 26;10:10486. doi: 10.1038/s41598-020-67001-w (PMC7320185; doi:10.1038/s41598-020-67001-w)
Supplement: Supplementary file 6 — Supplementary Table S4. [file 41598_2020_67001_MOESM6_ESM.pdf]

Supplementary Table S4. Genes in 51 CAD-associated loci (±250 kb around the lead SNP) proposed to be causal according to different lines of evidence.
Alternative gene names or non-coding RNA names are given in parenthesis after official gene symbols. Candidate genes with the most compelling evidence for their role in CAD according to literature data are shown in bold. In the studies by Brænne et al. [1], Lempiäinen et al. [2], and van der Harst et al. [3], possible candidate genes were linked to the prioritized CAD-associated SNPs (data on those SNPs located in the 51 studied loci can be found in Supplementary Table S3b). Arrows near gene names indicate that these genes have been linked to the same prioritized SNP in the locus or to SNPs in high LD with each other ($r^2 \geq 0.8$; Supplementary Table S3c). If there are two or more groups of such genes in the locus, single arrow indicates the genes linked to one SNP, double, triple, and quadruple arrows – genes linked to other SNPs (e.g., in locus 43). We also marked with arrows the genes found in SMR/HEIDI analysis if the top SNP (instrumental variable used for investigating relationships between gene expression and CAD) was the same or in high LD ($r^2 \geq 0.8$; Supplementary Table S3d) with SNPs prioritized in other studies.

	**Lead SNP**[¥]	**Chr: position**[*]	**Nearest**[†] **known gene**	**Genes found in SMR/HEIDI**[‡]	**Candidate genes from literature**	**Genes prioritized previously based on bioinformatics approaches** [1-3] **and found in gene-based association analysis** [4]	**Conclusion**
1	rs17114036	1: 56 962 821	*PLPP3 (PAP2B, PPAP2B)*	-	***PLPP3 (PAP2B, PPAP2B)***<br>*phospholipid phosphatase 3 (phosphatidic acid phosphatase type 2B)*<br>PLPP3 is a membrane glycoprotein that hydrolyzes extracellular lysophosphatidic acid (reviewed in [5]). Lysophosphatidic acid (LPA) is a potent bioactive phospholipid that accumulates in human atherosclerotic plaques, promotes atherosclerosis and has thrombogenic action [6,7]. PLPP3 is mechanosensitive, expression of its gene is dynamically modulated by atherorelevant hemodynamics. PLPP3 plays a critical role in promoting anti-inflammatory phenotype and maintaining vascular integrity of endothelial monolayer under athero-protective flow [5]. Hepatic Ppab2b (Plpp3) deletion alters plasma composition of several low-abundant pro-atherogenic lipids and worsens atherosclerosis in *Apoe*$^{-/-}$ mice [8]. *Ppab2b* was found to be differentially expressed in cells isolated from the “diseased” and “healthy” mouse aortas (up-regulated in pre-lesion endothelial cells and heterogeneous cells from atherosclerotic lesions and down-regulated in macrophages) taken from *Apoe*$^{-/-}$ /wild type mice or *Ldlr*$^{-/-}$ mice fed a Western/a chow diet, respectively [9].	**Lempiäinen et al.** [2]**, score range 2-54**<br>*PLPP3 (PAP2B)* (total score = 10)<br><br>**Svishcheva et al.** [4]<br>*PLPP3 (PAP2B)* (two datasets)	*PLPP3 (PAP2B)* is the causal gene.
2	rs602633	1: 109 821 511	*PSRC1*	▪ *PSRC1* ←<br>▪ *CELSR2* ←<br>▪ *PSMA5* ←	***SORT1***<br>*sortilin 1*<br>Sortilin-1 alters plasma LDL-C and VLDL particle levels by modulating hepatic VLDL secretion [10]. Increased hepatic sortilin-1 reduces hepatic apolipoprotein B secretion and increases LDL catabolism [11]. SORT1 was shown to be a high-affinity sorting receptor for PCSK9 (a protein that destines LDLR for degradation in lysosomes)	**Brænne et al.** [1]**, score range 1-11**<br>*CELSR2* (total score = 5) ←<br>*SORT1* (total score = 4) ←<br>*PSRC1* (total score = 4) ←<br>*MYBPHL* (total score = 2)<br><br>**Lempiäinen et al.** [2]**, score range 2-54**<br>*CELSR2* (total score = 10) ←	*SORT1* is the causal gene.<br><br>*PSRC1* and *CELSR2* might also be involved.

					modulating its secretion [12]. The role of SORT1 in atherosclerosis development was also supposed to be linked to attenuated secretion of proinflammatory cytokines (reviewed in [13]). *Sort1* expression was found to be down-regulated in macrophages isolated from aortas of *Ldlr*$^{-/-}$ mice fed a Western diet (“diseased” aortas) as compared to *Ldlr*$^{-/-}$ mice fed a chow diet (“healthy” aortas) [9].<br>The directionality of the effect of sortilin-1 on plasma cholesterol and its role in the secretion of hepatic lipoproteins remains controversial (reviewed in [14]).	**van der Harst et al.** [3]<br>*SORT1*[¶] ←<br>*CELSR2* ←<br>*PSRC1* ←<br>*SARS* ←<br>*ATXN7L2* ←<br><br>**Svishcheva et al.** [4]<br>*CELSR2* (two datasets)	
					CELSR2<br>*cadherin EGF LAG seven-pass G-type receptor 2*<br>*Celsr2* was found to be differentially expressed in cells isolated from the “diseased” and “healthy” mouse aortas: 1) up-regulated in pre-lesion endothelial cells from aortas of *Apoe*$^{-/-}$ mice as compared to wild type mice, 2) up-regulated in macrophages from aortas of *Ldlr*$^{-/-}$ mice fed a Western diet (“diseased” aortas) as compared to *Ldlr*$^{-/-}$ mice fed a chow diet (“healthy” aortas) [9].		
					PSRC1<br>*proline and serine rich coiled-coil 1*<br>*Psrc1* was found to be differentially expressed in cells isolated from the “diseased” and “healthy” mouse aortas: 1) up-regulated in pre-lesion endothelial cells and medial SMCs from *Apoe*$^{-/-}$ mice as compared to wild type mice, 2) down-regulated in macrophages from aortas of *Ldlr*$^{-/-}$ mice fed a Western diet (“diseased” aortas) as compared to *Ldlr*$^{-/-}$ mice fed a chow diet (“healthy” aortas) [9].<br>Transfection of RAW264.7 cells with adenovirus expressing Psrc1 reduced the cellular cholesterol content, increased the cholesterol efflux capacity and inhibited foam cell formation. Infecting *Apoe*$^{-/-}$ mice with this Psrc1-expressing adenovirus inhibited the development of atherosclerotic lesions and enhanced atherosclerotic plaque stability by modulating cholesterol transportation and inflammation in macrophages and the liver [15].		
3	rs4129267	1: 154 426 264	*IL6R*	▪ *IL6R* ←, ← ←	***IL6R***<br>*interleukin 6 receptor*<br>Interleukin-6 (IL-6) is a multifunctional pro-	**Brænne et al.** [1]**, score range 1-11**<br>*IL6R* (total score = 5) ←<br>*UBAP2L* (total score = 2) ←	*IL6R* is the causal gene.

					inflammatory cytokine. IL-6 signalling plays an important role in cardiovascular biology. IL-6 initiates the cascade of events leading to atherosclerosis and is implicated in the regulation of arterial blood pressure (reviewed in [16]).<br>Allele 358Ala of the common missense SNP rs2228145 (Asp358Ala) in the *IL6R* gene is associated with reduced CAD risk, increased IL-6R and IL-6 concentration, and reduced C-reactive protein and fibrinogen levels in circulation [17].	*ATP8B2* (total score = 2) ←<br>*CHTOP* (total score = 1) ←<br><br>**Lempiäinen et al.** [2]**, score range 2-54**<br>*IL6R* (total score = 10) ←, ← ←	
4	rs10919065	1: 169 093 557	*ATP1B1*	▪ *ATP1B1* ← ←<br>▪ *NME7* ←, ← ←	*ATP1B1*<br>*ATPase Na+/K+ transporting subunit beta 1*<br>Atp1b1 gene expression is downregulated during the regression phase of vascular calcification in rats [18]. Calcification of the coronary arteries plays a key role in the pathophysiology of atherosclerosis. The presence of coronary calcification is a surrogate marker of the overall plaque burden [19].<br>Atp1b1 was shown to be up-regulated in aortic SMCs from atherosclerosis-prone regions as compared to atherosclerosis-resistant regions in both $Apoe^{-/-}$ mice before plaque development and wild-type mice [20]. ATP1B1 is possibly involved in the regulation of blood pressure [21,22].	**Brænne et al.** [1]**, score range 1-11**<br>*ATP1B1* (total score = 4) ←<br>*NME7* (total score = 2) ←<br>*CCDC181* (total score = 1) ←	*ATP1B1* is the most likely causal gene.
5	rs6700559	1: 200 646 073	*DDX59-AS1*	▪ *DDX59-AS1 (RP11-92G12.3)* ←<br>▪ *DDX59* ←<br>▪ *CAMSAP2 (CAMSAP1L1)* ←	-	**Brænne et al.** [1]**, score range 1-11**<br>*KIF14* (total score = 4) ←<br>*CAMSAP2* (total score = 2) ←<br>*DDX59* (total score = 2) ←<br><br>**van der Harst et al.** [3]<br>*CAMSAP2*¶ ←<br>*DDX59* ←	Evidence is inconsistent.
6	rs2820315	1: 201 872 264	*LMOD1*	▪ *IPO9* ←<br>▪ *LMOD1* ←	*LMOD1*<br>*leiomodin 1*<br>*LMOD1* was found to be one of the genes which expression level reflects the altered phenotype of SMCs in atherosclerosis and could be early sensitive markers of SMC dedifferentiation. Downregulation of *LMOD1* in SMCs was functionally related to clinical symptoms of plaque instability, vascular injury, and to key molecular processes in atherosclerosis, such as apoptosis, shear stress, inflammatory stimuli, and lipid uptake [23].	**Brænne et al.** [1]**, score range 1-11**<br>*IPO9* (total score = 4) ←<br>*LMOD1* (total score = 2) ←<br>*SHISA4* (total score = 1) ←<br><br>**Lempiäinen et al.** [2]**, score range 2-54**<br>*IPO9* (total score = 10) ←	Evidence is inconsistent. *LMOD1* and *IPO9* can be involved.
7	rs16986953	2: 19 942 473	*LINC00954*	-	-	-	No evidence.

8	rs515135	2: 21 286 057	*APOB*	-	***APOB***<br>*apolipoprotein B*<br>Apolipoprotein B is a key component in lipid metabolism [24]. Apolipoprotein B is the major structural protein component of VLDLs secreted from the liver and the chylomicrons secreted from the intestine. High levels of plasma apo B-containing lipoproteins are a major risk for the development of atherosclerotic diseases [25]. Apolipoprotein B predicts ischemic cardiovascular events in both genders [24].	**Lempiäinen et al.** [2]**, score range 2-54**<br>*APOB* (total score = 32)<br>**Svishcheva et al.** [4]<br>*APOB* (one dataset)	*APOB* is the causal gene.
9	rs6544713	2: 44 073 881	*ABCG8*	-	***ABCG8***<br>*ATP binding cassette subfamily G member 8*<br>***ABCG5***<br>*ATP binding cassette subfamily G member 5*<br>ABCG5 and ABCG8 form an obligate heterodimer that limits intestinal absorption and facilitates biliary secretion of cholesterol and phytosterols. Upregulation of ABCG5 and ABCG8 levels can promote reverse cholesterol transport and reduce atherosclerosis (reviewed in [26]). Rare coding variants of *ABCG5* and *ABCG8* affect circulating cholesterol and phytosterols and influence the risk of CAD [27].	**Lempiäinen et al.** [2]**, score range 2-54**<br>*ABCG8* (total score = 34)<br>**Svishcheva et al.** [4]<br>*ABCG8* (two datasets)	*ABCG8/ABCG5* are the causal genes.
10	rs1561198	2: 85 809 989	*VAMP8*	▪ *GGCX* ←<br>▪ *VAMP5* ←<br>▪ *VAMP8* ←<br>▪ *USP39* ←<br>▪ *GNLY* ←	*GGCX*<br>*gamma-glutamyl carboxylase*<br>GGCX is an enzyme that carboxylates glutamate residues of vitamin K-dependent proteins. This modification makes them biologically active. Vitamin K-dependent proteins are implicated in blood coagulation, inflammation, and protection against vascular calcification [28]. Calcification of the coronary arteries plays a key role in the pathophysiology of atherosclerosis. The presence of coronary calcification is a surrogate marker of the overall plaque burden [19].<br>*VAMP8*<br>*vesicle associated membrane protein 8*<br>VAMP8 is involved in the fusion of synaptic vesicles with the presynaptic membrane. VAMP8 was shown to be required for platelet secretion [29]. Platelets participate in the pathogenesis of arteriosclerosis and in its progression by adhering to the damaged arteries and subsequently forming mural thrombi which are either swept away and	**Brænne et al.** [1]**, score range 1-11**<br>*GGCX* (total score = 5) ←<br>*VAMP5* (total score = 5) ←<br>*VAMP8* (total score = 5) ←<br>**Lempiäinen et al.** [2]**, score range 2-54**<br>*VAMP8* (total score = 42) ←<br>**Svishcheva et al.** [4]<br>*MAT2A* (one dataset)<br>*GGCX* (one dataset)<br>*VAMP5* (one dataset)	Evidence is inconsistent.

					embolize or are endothelialized and thus become part of the vessel wall. In addition, activated platelets release growth factors mediating SMC proliferation and migration as well as vasoconstriction [30].		
11	rs2252641	2: 145 801 461	*TEX41*	-	*ZEB2*<br>*zinc finger E-box binding homeobox 2*<br>ZEB2 transcription factor regulates fibroblast-to-myofibroblast phenoconversion (Ski–Zeb2–Meox2 pathway). Fibroblast-to-myofibroblast phenoconversoin in the heart is a crucial event in the onset of many cardiovascular diseases [31].	**Lempiäinen et al.** [2]**, score range 2-54**<br>*TEX41* (total score = 2)	Evidence is inconsistent.
12	rs2351524	2: 203 880 992	*NBEAL1*	▪ *ICA1L* ←<br>▪ *CARF* ←<br>▪ *NBEAL1* ←<br>▪ *CARF* ←<br>▪ *FAM117B* ←	*WDR12*<br>*WD repeat domain 12*<br>WDR12 expression was elevated in patients with cardiomyopathy, in rats post-infarction and in rats in Ang II-mediated hypertension. WDR12 triggers distinct deterioration of cardiac function in adult rat heart [32].	**Brænne et al.** [1]**, score range 1-11**<br>*NBEAL1* (total score = 4) ←<br>*WDR12* (total score = 4) ←<br>*CARF* (total score = 3) ←<br>*ALS2CR8* (total score = 2) ←<br>*ICA1L* (total score = 1) ←<br>**Lempiäinen et al.** [2]**, score range 2-54**<br>*ICA1L* (total score = 10) ←<br>**Svishcheva et al.** [4]<br>*NBEAL1* (two datasets)<br>*WDR12* (two datasets)	Evidence is inconsistent.
13	rs2306374	3: 138 119 952	*MRAS*	▪ *MRAS* ←<br>▪ *NME9* ←<br>▪ *ESYT3* ←	*MRAS*<br>*muscle RAS oncogene homolog*<br>M-ras is a member of the Ras family of small GTPases that function as signal transducers in multiple processes including cell growth and differentiation. M-ras is widely expressed in all tissues, with a very high expression in the cardiovascular system, especially in the heart. M-ras regulates cell polarity and migration through coordination of multiple signaling pathways [33]. M-ras was shown to play a crucial role in adhesion signaling [34], which is important for the atherosclerotic process [35].	**Brænne et al.** [1]**, score range 1-11**<br>*MRAS* (total score = 5) ←<br>*CEP70* (total score = 2) ←<br>**Lempiäinen et al.** [2]**, score range 2-54**<br>*MRAS* (total score = 34) ←<br>**Svishcheva et al.** [4]<br>*MRAS* (one dataset)	*MRAS* is the causal gene.
14	rs1429141	4: 148 288 067	*MIR548G*	-	*EDNRA (ETA)*<br>*endothelin receptor type A*<br>ETA receptors play a role in potent and long-lasting vasoconstriction. Increased level of *EDNRA* mRNA in the internal mammary artery of CAD patients was associated with systemic hypertension [36]. ETA receptors are present in human aortic vascular endothelial cells and modulate intracellular calcium [37]. Endothelin-1 activates ETA receptors on human vascular smooth muscle	**Lempiäinen et al.** [2]**, score range 2-54**<br>*EDNRA (ETA)* (total score = 34)<br>**Svishcheva et al.** [4]<br>*EDNRA (ETA)* (one dataset)	*EDNRA* is the causal gene.

					cells and stimulates changes in glycosaminoglycan chain structure that increase binding to LDL [38].		
15	rs7692387	4: 156 635 309	*GUCY1A1*	▪ *GUCY1A3* ←	***GUCY1A3***<br>*guanylate cyclase 1 soluble subunit alpha (α1-sGC)*<br>α1-sGC is a key enzyme in the nitric oxide/cGMP signaling pathway. cGMP induces vasodilation and inhibits platelet activation. Impairment of the nitric oxide/cGMP signaling influences myocardial infarction risk, possibly through accelerated thrombus formation [39]. Linkage was demonstrated between GUCY1A3 rare loss-of-function mutations and familial CAD [39].<br>Rs7692387 is located in an intronic site that modulates *GUCY1A3* promoter activity. The transcription factor ZEB1 binds preferentially to the non-risk allele, leading to an increase in *GUCY1A3* expression, higher sGC levels, and higher sGC activity after stimulation. Augmented sGC expression is linked to lower risk of atherosclerosis [40].	**Lempiäinen et al.** [2]**, score range 2-54**<br>*GUCY1A3* (total score = 42) ←	*GUCY1A3* is the causal gene.
16	rs273909	5: 131 667 353	*SLC22A4*<br>*MIR3936HG*	-	-	**Lempiäinen et al.** [2]**, score range 2-54**<br>*SLC22A5* (total score = 10)	Insufficient evidence.
17	rs246600	5: 142 516 897	*ARHGAP26*	-	-	**van der Harst et al.** [3]<br>*HMHB1*	Insufficient evidence.
18	rs7751826	6: 12 900 977	*PHACTR1*	▪ *RP1-257A7.5* ←<br>▪ *RP1-257A7.4* ←<br>▪ *PHACTR1* ←	***PHACTR1***<br>*phosphatase and actin regulator 1*<br>PHACTR1 plays an important role in cell migration and survival. Upregulation of PHACTR1 was associated with the progression of calcification. PHACTR1 is an inhibitory mediator of protein phosphatase 1 (PP1). PP1 regulates the synthesis of nitric oxide in endothelial cells, which is essential for endothelial function (reviewed in [41,42]). PHACTR1 is highly expressed in human atherosclerotic plaque macrophages, lipid-laden foam cells, adventitial lymphocytes and endothelial cells, and its expression is regulated by atherogenic and inflammatory stimuli [43]. *Phactr1* expression was found to be up-regulated in pre-lesion endothelial cells and heterogeneous cells from atherosclerotic lesions isolated from aortas of *Apoe*$^{-/-}$ mice ("diseased" aortas) as compared to wild type mice ("healthy" aortas) [9].	**Lempiäinen et al.** [2]**, score range 2-54**<br>*PHACTR1* (total score = 2) ←, ← ←<br><br>**van der Harst et al.** [3]<br>*EDN1*[¶] ← ←<br>*TBC1D7* ← ←<br>*PHACTR1* ← ←<br>*GFOD1* ← ←<br><br>**Svishcheva et al.** [4]<br>*PHACTR1* (two datasets)	*PHACTR1* is the causal gene.

19	rs10947789	6: 39 174 922	*KCNK5*	-	-	**Lempiäinen et al.** [2]**, score range 2-54**<br>*KCNK5* (total score = 2)	Insufficient evidence.
20	rs2327429	6: 134 209 837	*TARID*	▪ *TCF21* ←<br>▪ *RP3-323P13.2* ←<br>▪ *RP1-283K11.3* ←	***TCF21***<br>*transcription factor 21*<br>*TCF21* is expressed in mesodermal cells in the proepicardial organ that give rise to coronary artery SMC. *Tcf21* knockout animals exhibit a dramatic failure of cardiac fibroblast. Early expression of *Tcf21* is supposed to be important for expansion of the SMC compartment of the coronary circulation (reviewed in [44]). TCF21 target regions were shown to be over-represented among CAD associated loci found in GWAS, and TCF21 target CAD loci were enriched for the genes involved in vascular wall biological processes [44]. *Tcf21* expression was found to be up-regulated in pre-lesion endothelial cells isolated from aortas of *Apoe*$^{-/-}$ mice ("diseased" aortas) as compared to wild type mice ("healthy" aortas) [9].	**Lempiäinen et al.** [2]**, score range 2-54**<br>*TCF21* (total score = 10) ←	*TCF21* is the causal gene.<br><br>*RP3-323P13.2* might also be involved.
21	rs3103349$^{\#}$	6: 160 740 721	*SLC22A3*	▪ *LPA*	***LPA (APOA)***<br>*lipoprotein(a)*<br>The *LPA* gene encodes apolipoprotein(a), which constitutes a substantial portion of lipoprotein(a) – a cholesterol-rich LDL-like particle. Concentration of circulating lipoprotein(a) is associated with coronary heart disease and stroke [45,46]. Genetically lowered lipoprotein(a) is associated with a lower risk of peripheral vascular disease, stroke, heart failure, and aortic stenosis [47].	**Brænne et al.** [1]**, score range 1-11**<br>*SLC22A3* (total score = 5) ← ←<br>*AL591069.5* (total score = 1) ← ←<br><br>**Lempiäinen et al.** [2]**, score range 2-54**<br>*LPA* (total score = 54)<br>*LPAL2 pseudogene* (total score = 10) ← ←<br>*IGF2R* (total score = 2)<br>*SLC22A2* (total score = 2)<br>*SLC22A3* (total score = 2)<br><br>**Svishcheva et al.** [4]<br>*LPA* (two datasets)<br>*SLC22A1* (two datasets)<br>*SLC22A2* (two datasets)<br>*SLC22A3* (two datasets)<br>*IGF2R* (one dataset)	*LPA* is the causal gene.<br><br>*SLC22A3*, *SLC22A2*, *SLC22A1* might also be involved.
22	rs10455872$^{\#}$	6: 161 010 118	*LPA*	▪ *LPA* ← ←	***LPA (APOA)***<br>*lipoprotein(a)*<br>The *LPA* gene encodes apolipoprotein(a), which constitutes a substantial portion of lipoprotein(a) – a cholesterol-rich LDL-like particle. Concentration of circulating lipoprotein(a) is associated with coronary heart disease and stroke [45,46]. Genetically lowered lipoprotein(a) is associated with a lower risk of peripheral vascular disease, stroke, heart failure, and aortic stenosis [47].	**Brænne et al.** [1]**, score range 1-11**<br>*PLG* (total score = 6) ←<br>*SLC22A3* (total score = 5) ← ←<br>*LPAL2 pseudogene* (total score = 4) ←<br>*AL591069.5* (total score = 1) ← ←<br><br>**Lempiäinen et al.** [2]**, score range 2-54**<br>*LPA* (total score = 54)<br>*PLG* (total score = 46) ←<br>*LPAL2 pseudogene* (total score = 10) ← ←<br>*SLC22A3* (total score = 2)<br><br>**Svishcheva et al.** [4]	*LPA* is the causal gene.<br><br>*PLG* and *SLC22A3* might also be involved.

						SLC22A3 (two datasets)<br>*LPA* (two datasets)<br>*PLG* (two datasets)	
23	rs11556924	7: 129 663 496	*ZC3HC1 (NIPA)*	▪ *KLHDC10* ←	*KLHDC10*<br>*kelch domain containing 10*<br>KLHDC10 participates in the oxidative stress-induced cell death. KLHDC10 is required for H_2O_2-induced sustained activation of ASK1 and apoptosis [48]. In mice, KLHDC10 deficiency significantly reduced the induction of IL-6 mRNA expression in the spleen and IL-6 release into the serum and protected against TNFα-induced systemic inflammation [49].<br><br>*ZC3HC1 (NIPA)*<br>*zinc finger C3HC-type containing 1*<br>ZC3HC1 is a component of an SCF-type E3 ubiquitin ligase complex that regulates the onset of cell division. Functional missense SNP rs11556924 in the *ZC3HC1* gene alters the dynamics of cell cycle regulation (impacts on the progression of the cell through mitosis) [50]. Cell cycle regulation is important for determination of vascular wall structure, proliferative response to vascular injury, regulation of plaque growth and the risk of its rupture. Dysregulation of cell cycle can be therefore involved in CAD development [50,51].<br>Rare mutation in *ZC3HC1* was found to be linked to CAD in families suffering from autosomal dominantly inherited CAD [52].	**Brænne et al.** [1]**, score range 1-11**<br>*ZC3HC1 (NIPA)* (total score = 4) ←<br><br>**Lempiäinen et al.** [2]**, score range 2-54**<br>*ZC3HC1 (NIPA)* (total score = 22) ←<br><br>**van der Harst et al.** [3]<br>*ZC3HC1*¶ ←<br>*NRF1* ←<br>*KLF14* ←<br><br>**Svishcheva et al.** [4]<br>*ZC3HC1* (one dataset)	*ZC3HC1 (NIPA)* is the most likely causal gene.<br><br>*KLHDC10* might also be involved.
24	rs10237377	7: 139 757 136	*PARP12*	-	*TBXAS1*<br>*thromboxane A synthase 1*<br>TBXAS1 catalyzes the conversion of prostglandin H2 to thromboxane A2, a potent vasoconstrictor and inducer of platelet aggregation. Thromboxane A2 release is enhanced in a variety of cardiovascular diseases, such as acute myocardial infarction (reviewed in [53]). *TBXAS1* gene expression were shown to be associated with the risk of large artery-atherosclerosis stroke [54].	**Brænne et al.** [1]**, score range 1-11**<br>*TBXAS1* (total score = 5)<br><br>**Lempiäinen et al.** [2]**, score range 2-54**<br>*PARP12* (total score = 10) ←<br>*TBXAS1* (total score = 10) ←	*TBXAS1* is the most likely causal gene.
25	rs11204085	8: 19 940 796	*SLC18A1*	▪ *LPL*	***LPL***<br>*lipoprotein lipase*<br>LPL is an enzyme anchored to the endothelial cells lining blood capillaries. LPL activity is the rate-determining step in the clearance of dietary fat from the circulation. LPL hydrolyzes 1) chylomicrons to free fatty acids and chylomicron remnant particles, 2) VLDL particles to IDL, which	**Brænne et al.** [1]**, score range 1-11**<br>*LPL* (total score = 8) ←<br><br>**Lempiäinen et al.** [2]**, score range 2-54**<br>*LPL* (total score = 42) ←<br><br>**Svishcheva et al.** [4]<br>*LPL* (one dataset)	*LPL* is the causal gene.

					are subsequently degraded into LDL particles by hepatic lipase. Both chylomicron remnants and LDL can penetrate the vessel wall and propagate atherosclerotic plaque. Individuals harboring a heterozygous damaging mutation in the *LPL* gene have increased levels of circulating triglycerides and increased risk of CAD (reviewed in [55]).		
26	rs2954032	8: 126 493 392	*TRIB1*	-	***TRIB1***<br>*tribbles pseudokinase 1*<br>In mice, Trib1 deficiency increases plasma cholesterol and triglycerides, and overexpression of Trib1 in mouse liver reduces these parameters. TRIB1 induces the reduction in plasma VLDL and LDL fraction. Increased expression of Trib1 reduces lipogenesis and VLDL secretion (reviewed in [56]). *Trib1* was found to be differentially expressed in cells isolated from the “diseased” and “healthy” mouse aortas: 1) up-regulated in heterogeneous cells from atherosclerotic lesions of *Apoe*$^{-/-}$ mice as compared to wild type mice, 2) up-regulated in macrophages from aortas of *Ldlr*$^{-/-}$ mice fed a Western diet (“diseased” aortas) as compared to *Ldlr*$^{-/-}$ mice fed a chow diet (“healthy” aortas) [9].	-	*TRIB1* is the causal gene.
27	rs3218020	9: 21 997 872	*CDKN2B-AS1 (ANRIL)*	-	***CDKN2B-AS1 (ANRIL)***<br>*CDKN2B antisense RNA 1 (antisense RNA in the INK4 locus)*<br>*ANRIL* is expressed in tissues and cell types that are affected by atherosclerosis [57]. Different splicing variants of ANRIL are suggested to coordinate tissue remodeling by modulating the expression of genes involved in cell proliferation, apoptosis, extra-cellular matrix remodeling and inflammatory response [58]. In particular, expression of the long and short *ANRIL* variants was correlated with expression of genes controlling cellular proliferation pathways (*CDKN2B*, *TDGF1*) [59]. Risk alleles for atherosclerosis-related phenotypes (in 9p21 locus) were associated with lower expression of *ANRIL* in blood [60] and primary cultures of vSMCs [61] as well as with increased vSMC proliferation [61]. Common carotid artery stenosis was associated with lower expression of *ANRIL* (exons 1-2), and *ANRIL* knock-down in vSMC caused significant variation in expression of *CDKN2A/B* and reduction of cell growth in vitro [60]. In another study, expression	**Brænne et al.** [1]**, score range 1-11**<br>*CDKN2B* (total score = 8) ←**,** ← ←<br>*CDKN2A* (total score = 5) ←<br><br>**Lempiäinen et al.** [2]**, score range 2-54**<br>*CDKN2B* (total score = 9) ←<br>*CDKN2B-AS1* (total score = 2) ← ←<br><br>**van der Harst et al.** [3]<br>*CDKN2B*[¶] ← ← ←<br>*MTAP*← ← ←<br><br>**Svishcheva et al.** [4]<br>*CDKN2B* (two datasets)<br>*CDKN2A* (two datasets)<br>*MTAP* (one dataset)	*CDKN2B-AS1* is the causal gene, which regulates *CDKN2B* and *CDKN2A* expression.

					of several *ANRIL* transcripts in peripheral blood mononuclear cells was significantly increased in carriers of the CAD risk haplotype and was directly correlated with severity of atherosclerosis [62].<br>Linear form of ANRIL was shown to confer increased risk of atherosclerosis, whereas circular form of ANRIL was found to be atheroprotective due to induction of apoptosis and inhibition of proliferation [63].<br>In mice, deletion of *Cdnk2b* (ANRIL target gene) promoted advanced development of atherosclerotic plaques composed of large necrotic cores [64]. Targeted deletion of the orthologous 9p21 region in these animals markedly decreased expression of *Cdnk2a*/b and increased the proliferation of aortic SMCs and diminished their senescence (a cellular phenotype consistent with accelerated CAD pathogenesis) [65].		
28	rs579459	9: 136 154 168	*ABO*	▪ *SURF1* ←<br>▪ *ABO* ←	*ABO*<br>*alpha 1-3-N-acetylgalactosaminyltransferase and alpha 1-3-galactosyltransferase*<br>*ABO* gene encodes glycosyltransferase responsible for ABO blood group determination. ABO blood group system is linked with thrombotic vascular diseases. Candidate mechanism for this link is modulation of circulating levels of vWF, which regulates platelet adhesion and stabilizes FVIII coagulation factor (O-allele carriers have markedly decreased level of both factors as compared to non-O individuals) [66]. Besides coagulation, vWF has many other functions, including direct and indirect activation of inflammatory response [67].<br>Systematic review and meta-analysis showed that the risk of CAD was significantly higher in blood group A and lower in blood group O [68]. Subjects of non-O type were demonstrated to have higher levels of total cholesterol, low-density lipoprotein cholesterol, and non–high-density lipoprotein cholesterol than O type subjects [69]. Besides this, longitudinal study in a Japanese cohort found elevated total cholesterol levels in A-type individuals compared to non-A subjects [70].	**Lempiäinen et al.** [2]**, score range 2-54**<br>*DDX31* (total score = 8) ←<br>*SURF1* (total score = 8) ←<br>*SURF6* (total score = 8) ←<br><br>**Svishcheva et al.** [4]<br>*ABO* (one dataset)<br>*ADAMTS13* (one dataset)	Evidence is inconsistent.
					ADAMTS13<br>*ADAM metallopeptidase with thrombospondin type 1 motif 13*<br>ADAMTS13 cleaves highly adhesive ultra large vWF and has a powerful antithrombotic activity		

					[71]. ADAMTS13 reduces vascular inflammation and the development of early atherosclerosis in mice [72], exerts protective anti-inflammatory effect on myocardial ischemia/reperfusion injury [73], and controls vascular remodeling by modifying vWF reactivity during stroke recovery [74]. Increase in the ratio of VWF/ADAMTS13 was shown to be associated with the occurrence of cardiovascular events after acute myocardial infarction [75,76].		
29	rs2505083	10: 30 335 122	*JCAD (KIAA1462)*	▪ *JCAD (KIAA1462)* ←	*JCAD (KIAA1462)*<br>*junctional cadherin 5 associated*<br>JCAD is expressed at cell-cell junctions in endothelial cells. *In vitro* and *in vivo* (mice) studies suggest that JCAD has a redundant functional role in physiological angiogenesis but has a pivotal role in pathological angiogenic process after birth [77]. *JCAD* knockdown in endothelial cells was shown to affect key phenotypes related to atherosclerosis including proliferation, migration, apoptosis, tube formation, and monocyte binding. JCAD negatively regulates Hippo signaling in endothelial cells and may contribute to atherosclerosis by mediating YAP activity and endothelial dysfunction [78].<br>*Kiaa1462* expression was found to be up-regulated in pre-lesion endothelial cells and heterogeneous cells from atherosclerotic lesions isolated from aortas of *Apoe*$^{-/-}$ mice (“diseased” aortas) as compared to wild type mice (“healthy” aortas) [9].	**Brænne et al.** [1]**, score range 1-11**<br>*JCAD (KIAA1462)* (total score = 6) ←<br>**Lempiäinen et al.** [2]**, score range 2-54**<br>*JCAD (KIAA1462)* (total score = 6) ←<br>**Svishcheva et al.** [4]<br>*JCAD (KIAA1462)* (one dataset)	*JCAD* is the causal gene.
30	rs10793513	10: 44 494 546	*LINC00841*	-	-	-	No evidence.
31	rs523297	10: 44 756 557	*CXCL12*	-	***CXCL12***<br>*C-X-C motif chemokine ligand 12*<br>CXCL12 (SDF-1) is a chemokine involved in inflammatory signaling pathways and playing important roles in hematopoiesis, stem cell mobilization, angiogenesis, etc. CXCL12 is of fundamental importance in vascular repair and remodeling. CXCL12/CXCR4 axis was clearly shown to be atheroprotective (reviewed in [79]). Plasma levels of CXCL12 were significantly decreased in patients with CAD compared with healthy control subjects [80]. Atheroprotective effect of CXCL12 was suggested to realize through the recruitment of progenitor cells and preventing endothelial apoptosis [81].<br>*Cxcl12* was found to be differentially expressed in	-	*CXCL12* is the causal gene.

					cells isolated from the "diseased" and "healthy" mouse aortas: 1) up-regulated in pre-lesion endothelial cells and medial SMCs of *Apoe*$^{-/-}$ mice as compared to wild type mice, 2) down-regulated in macrophages from aortas of *Ldlr*$^{-/-}$ mice fed a Western diet ("diseased" aortas) as compared to *Ldlr*$^{-/-}$ mice fed a chow diet ("healthy" aortas) [9].<br>*CXCL12* was found to be one of the top key regulators of CAD in the study based on integrative genomics approach [82].		
32	rs2246833	10: 91 005 854	*LIPA*	▪ *LIPA* ←<br>▪ *IFIT1* ←<br>▪ *IFIT5* ←	***LIPA***<br>*lipase A, lysosomal acid type*<br>Lysosomal acid lipase (LAL) hydrolyzes cholesteryl esters and triglycerides into free cholesterol, glycerol, and free fatty acids and prevents lipid accumulation in various tissues and cell types, including macrophages. Loss-of-function mutations in *LIPA* result in accelerated atherosclerosis in humans, and overexpression of *Lipa* reduces atherosclerosis in mice. CAD-associated coding variant rs1051338 (which is in LD with rs2246833, r^2 = 0.87 in Europeans according to LDlink, https://ldlink.nci.nih.gov/) causes reduced lysosomal LAL protein level and activity because of increased LAL degradation [83].<br>*Lipa* expression was found to be up-regulated in pre-lesion endothelial cells and heterogeneous cells from atherosclerotic lesions isolated from aortas of *Apoe*$^{-/-}$ mice ("diseased" aortas) as compared to wild type mice ("healthy" aortas) [9].	**Brænne et al.** [1]**, score range 1-11**<br>*LIPA* (total score = 9) ←<br>**Lempiäinen et al.** [2]**, score range 2-54**<br>*LIPA* (total score = 46) ←<br>**Svishcheva et al.** [4]<br>*LIPA* (one dataset)	*LIPA* is the causal gene.
33	rs11191447	10: 104 652 323	*BORCS7-ASMT*<br>*AS3MT*	▪ *TMEM180 (MFSD13A)* ←<br>▪ *ARL3* ←<br>▪ *NT5C2* ←<br>▪ *MARCKSL1P1 pseudogene* ←	*CYP17A1*<br>*cytochrome P450 family 17 subfamily A member 1*<br>CYP17A1 is a key enzyme in the steroidogenic pathway that produces progestins, mineralocorticoids, glucocorticoids, androgens, and estrogens.<br>Polymorphism rs138009835 (which is in LD with rs11191447, r^2 = 0.95 in Europeans according to LDlink, https://ldlink.nci.nih.gov/) is associated with changes in aldosterone level and has functional effects on the *CYP17A1* gene transcription *in vitro* [84]. Aldosterone plays a fundamental role in salt and water homeostasis, blood pressure regulation, and cardiovascular remodeling [85]. High blood pressure is a major modifiable risk factor for all clinical manifestations	**Brænne et al.** [1]**, score range 1-11**<br>*NT5C2* (total score = 5) ←<br>*CNNM2* (total score = 4) ←<br>**Lempiäinen et al.** [2]**, score range 2-54**<br>*CYP17A1* (total score = 34) ←<br>**Svishcheva et al.** [4]<br>*CYP17A1* (one dataset)<br>*CNNM2* (one dataset)<br>*AS3MT* (one dataset)	*CYP17A1* is the most likely causal gene.

					of CAD [86].<br>*Cyp17a1* expression was found to be down-regulated in pre-lesion endothelial cells isolated from aortas of *Apoe*$^{-/-}$ mice ("diseased" aortas) as compared to those of wild type mice ("healthy" aortas) [9].		
34	rs12801636	11: 65 391 317	*PCNX3*	▪ *SIPA1* ←<br>▪ *MAP3K11* ←<br>▪ *CTSW* ← ←<br>▪ *FIBP* ← ←	*RELA*<br>*RELA proto-oncogene, NF-kB subunit*<br>RELA (p65) is a subunit of NF-kB protein complex. NF-kB is a pleiotropic transcription factor activated by various stimuli including ROS and cytokines (such as TNF-α and IL-1). NF-kB controls the expression of many pro-inflammatory genes coding cytokines, chemokines, adhesion molecules, and plays a central role in inflammation [87].	**Brænne et al.** [1]**, score range 1-11**<br>*RELA* (total score = 6) ←<br>*SIPA1* (total score = 4) ←<br>*OVOL1* (total score = 2) ←<br>*PCNXL3* (total score = 1) ←<br><br>**Lempiäinen et al.** [2]**, score range 2-54**<br>*RELA* (total score = 40) ←<br><br>**van der Harst et al.** [3]<br>*EHBP1L1*¶ ←	Evidence is inconsistent.
35	rs974819	11: 103 660 567	*MIR4693*	▪ *PDGFD* ←<br>▪ *RP11-563P16.1* ←	***PDGFD***<br>*platelet derived growth factor D*<br>PDGF-D plays critical roles in the cardiovascular system as angiogenic and survival factor [88]. *PDGFD* is expressed in macrophages, SMCs, and endothelial cells in human atherosclerotic plaques and is suggested to stimulate atherosclerosis by promoting MMP activity and monocyte migration [89]. Overexpression of the core domain of PDGF-D in mouse hearts caused vascular remodeling, including dilation of vessels, increased density of SMC-coated vessels, and proliferation of vSMCs, and thickening of arterial walls [90]. *Pdgfd* expression was found to be up-regulated in heterogeneous cells from atherosclerotic lesions isolated from aortas of *Apoe*$^{-/-}$ mice ("diseased" aortas) as compared to wild type mice ("healthy" aortas) [9].	**van der Harst et al.** [3]<br>*PDGFD*¶ ←	*PDGFD* is the causal gene.
36	rs3184504§	12: 111 884 608	*SH2B3 (LNK)*	▪ *SH2B3* ← ←<br>▪ *ALDH2* ←<br>▪ *TMEM116* ←<br>▪ *MAPKAPK5* ←<br>▪ *RP3-462E2.3* ←	*SH2B3 (LNK)*<br>*SH2B adaptor protein 3*<br>SH2B3 moderates growth factor and cytokine receptor-mediated signaling. In endothelial cells, it is a negative regulator of TNF signaling that reduces proinflammatory phenotype and prevents these cells from apoptosis. In both platelets and endothelial cells, SH2B3 modulates integrin signaling and actin cytoskeleton organization with an impact on cell adhesion, migration and thrombosis [91]. A loss-of-function variant in *SH2B3* promotes platelet and leukocyte production. In mice, hematopoietic Lnk (Sh2b3) deficiency	**Brænne et al.** [1]**, score range 1-11**<br>*SH2B3* (total score = 5) ← ←<br>*ATXN2* (total score = 4) ← ←<br>*FLJ21127* (total score = 1) ← ←<br><br>**Lempiäinen et al.** [2]**, score range 2-54**<br>*SH2B3* (total score = 14) ← ←<br><br>**Svishcheva et al.** [4]<br>*ATXN2* (two datasets)<br>*SH2B3* (one dataset)	*SH2B3* is the most likely causal gene.<br><br>*ATXN2* might also be involved.

					leads to accelerated arterial thrombosis and atherosclerosis, but only in the setting of hypercholesterolemia [92]. Rs3184504 is a missense SNP located in the *SH2B3* gene (leads to R262W amino acid substitution).<br>*Sh2b3* was found to be differentially expressed in cells isolated from the “diseased” and “healthy” mouse aortas: 1) up-regulated in pre-lesion endothelial cells of *Apoe*$^{-/-}$ mice as compared to wild type mice, 2) down-regulated in macrophages from aortas of *Ldlr*$^{-/-}$ mice fed a Western diet (“diseased” aortas) as compared to *Ldlr*$^{-/-}$ mice fed a chow diet (“healthy” aortas) [9].		
					ATXN2<br>*ataxin-2*<br>Ataxin-2 knock-out mice display abdominal obesity, hepatosteatosis, and increased serum levels of cholesterol, suggesting an elevated diabetic and vascular risk in these animals [93].		
37	rs441[§]	12: 112 228 849	*ALDH2*	▪ *TMEM116* ←<br>▪ *ERP29* ←<br>▪ *SH2B3*<br>▪ *ALDH2* ←<br>▪ *MAPKAPK5* ←	*ATXN2*<br>*ataxin-2*<br>Ataxin-2 knock-out mice display abdominal obesity, hepatosteatosis, and increased serum levels of cholesterol, suggesting an elevated diabetic and vascular risk in these animals [93].	**Brænne et al.** [1]**, score range 1-11**<br>*ALDH2* (total score = 6) ←<br>*SH2B3* (total score = 5) ←<br>*TMEM116* (total score = 4) ←<br>*BRAP* (total score = 4) ←<br>*MAPKAPK5* (total score = 4) ←<br>*HECTD4* (total score = 2) ←<br>*C12ORF30* (total score = 2) ←<br><br>**Svishcheva et al.** [4]<br>*ATXN2* (two datasets)<br>*TMEM116* (one dataset)<br>*NAA25* (one dataset)	Evidence is inconsistent.
					ALDH2<br>*aldehyde dehydrogenase 2 family member*<br>ALDH2 protects against oxidative stress [94,95] and prevents ROS-induced vascular contraction in angiotensin-II hypertensive mice [96]. ROS appear to be important modulators of atherovascular disease in all stages of its development (reviewed in [97]).		
					MAPKAPK5<br>*MAPK activated protein kinase 5*<br>*Mapkapk5* expression was found to be up-regulated in pre-lesion endothelial cells and heterogeneous cells from atherosclerotic lesions isolated from aortas of *Apoe*$^{-/-}$ mice (“diseased” aortas) as compared to wild type mice (“healthy” aortas) [9].		
38	rs2258287	12: 121 454 313	*C12ORF43*	▪ *OASL* ←<br>▪ *C12ORF43* ←<br>▪ *COQ5*	*HNF1A*<br>*HNF1 homeobox A*<br>HNF1α is a transcription factor highly expressed in the liver and required for the expression of several liver-specific genes. It was shown to bind at least 222 target genes in hepatocytes, including the genes which products are involved in central rate-	**Brænne et al.** [1]**, score range 1-11**<br>*HNF1A* (total score = 4) ← ←<br><br>**Lempiäinen et al.** [2]**, score range 2-54**<br>*C12ORF43* (total score = 8) ← ←	*HNF1A* is the most likely causal gene.

					limiting steps in gluconeogenesis and associated pathways as well as the genes which products participate in lipid metabolism (synthesis of cholesterol and apolipoproteins), detoxification, and synthesis of serum proteins (albumin, complements, and coagulation factors) [98]. Defects in the *HNF1A* gene cause maturity onset diabetes of the young type 3 (MODY3) [99]. HNF1α was shown to regulate cytokine-driven expression of C-reactive protein, that is a biomarker of inflammation associated with the increased risk of cardiovascular events. HNF1α was supposed to play a key role in linking metabolic and inflammatory pathways underlying the pathogenesis of coronary heart disease (reviewed in [100]).		
39	rs11057830	12: 125 307 053	*SCARB1*	-	*SCARB1*<br>*scavenger receptor class B member 1*<br>SCARB1 is a plasma membrane receptor for HDL-cholesterol that mediates cholesterol transfer to and from HDL. HDL cholesterol levels are inversely associated with the risk of CAD, but a Mendelian randomization study provided evidence that this association is not reflective of a causal relationship [101].	**Brænne et al.** [1]**, score range 1-11**<br>*SCARB1* (total score = 6) ←<br>**Lempiäinen et al.** [2]**, score range 2-54**<br>*SCARB1* (total score = 34) ←<br>*DHX37* (total score = 2)<br>**Svishcheva et al.** [4]<br>*SCARB1* (one dataset)	*SCARB1* is the causal gene.
40	rs9319428	13: 28 973 621	*FLT1 (VEGFR1)*	-	*FLT1 (VEGFR1)*<br>*fms related tyrosine kinase 1 (vascular endothelial growth factor receptor 1)*<br>VEGFR1 binds to vascular endothelial growth factor (VEGF-A and VEGF-B) (may act as a decoy receptor [102,103]) and placental growth factor (PlGF) [103] and plays an important role in angiogenesis and vasculogenesis. Expression of *FLT1* is found in vascular endothelial cells, placental trophoblast cells and peripheral blood monocytes.<br>VEGF was reported to accelerate atherosclerotic progression [105-107].<br>PlGF is a pleiotropic cytokine with a pro-inflammatory activity. Loss of PlGF was found to delay atherosclerotic lesion development and inhibit macrophage infiltration [108]. PlGF-expression within human atherosclerot7ic lesions was associated with plaque inflammation and microvascular density, suggesting a role for PlGF in plaque destabilization [109].	**Lempiäinen et al.** [2]**, score range 2-54**<br>*FLT1* (total score = 34)	*FLT1* is the causal gene.

41	rs9515203	13: 111 049 623	*COL4A2*	-	*COL4A2, COL4A1*<br>*collagen type IV alpha 2 chain,*<br>*collagen type IV alpha 1 chain*<br>COL4A1 and COL4A2 are subunits of the type IV collagen, a main collagen component of the basement membrane. In the normal arterial wall, type IV collagen inhibits SMCs proliferation [110].<br>Atherosclerotic plaques with the rs4773144 G/G genotype had lower collagen IV abundance and thinner fibrous cap, a hallmark of unstable, rupture-prone plaques. CAD-associated rs4773144 G allele (not in LD with rs9515203, r^2 = 0.0005 in Europeans according to LDlink, https://ldlink.nci.nih.gov/) was associated with lower *COL4A2* and *COL4A1* expression levels, higher SMC apoptosis rates, and higher rates of myocardial infarction [111].<br>*Col4a1* and *Col4a2* was found to be differentially expressed in cells isolated from the “diseased” and “healthy” mouse aortas (*Col4a1*: up-regulated in pre-lesion endothelial cells; *Col4a2*: up-regulated in pre-lesion endothelial cells, heterogeneous cells from atherosclerotic lesions, and macrophages) taken from *Apoe*$^{-/-}$ /wild type mice or *Ldlr*$^{-/-}$ mice fed a Western/a chow diet, respectively [9].<br>*COL4A2* was found to be one of the top key regulators of CAD in the study based on integrative genomics approach [82].	**Brænne et al.** [1]**, score range 1-11**<br>*IRS2* (total score = 4)<br><br>**Lempiäinen et al.** [2]**, score 2-54**<br>*ANKRD10* (total score = 8) ←<br>*COL4A1* (total score = 2) ← ←<br>*COL4A2* (total score = 2) ←, ← ←<br><br>**Svishcheva et al.** [4]<br>*COL4A2* (two datasets)<br>*COL4A1* (one dataset)	*COL4A2* and *COL4A1* are the causal genes.
42	rs2895811	14: 100 133 942	*HHIPL1*		*HHIPL1*<br>*HHIP like 1*<br>*HHIPL1* belongs to the glucose/sorbosone dehydrogenase family. In atherosclerotic mouse aortas, *Hhipl1* expression increased with disease progression. *Hhipl1*-/- mice displayed a reduction of 57% (+/-28%) in lesion area compared with controls on *Ldlr*$^{-/-}$ background and 49% (+/-28%) reduction on *Apoe*$^{-/-}$ background [112].	**Brænne et al.** [1]**, score range 1-11**<br>*YY1* (total score = 6) ←<br>*EML1* (total score = 2)<br><br>**Lempiäinen et al.** [2]**, score 2-54**<br>*HHIPL1* (total score = 6) ←	*HHIPL1* is the most likely causal gene.
43	rs7178051	15: 79 118 296	*ADAMTS7*	▪ *ADAMTS7* ←<br>▪ *CTSH* ←<br>▪ *RP11-160C18.2 (pseudogene)* ←<br>▪ *MORF4L1* ←	***ADAMTS7***<br>*ADAM metallopeptidase with thrombospondin type 1 motif 7*<br>ADAMTS7 belongs to a family of proteins involved in proteolysis and blood vessel wall remodeling. ADAMTS7 accumulates in SMCs in coronary and carotid atherosclerotic plaques [113]. *Adamts7* knockout hyperlipidemic mice	**Brænne et al.** [1]**, score range 1-11**<br>*ADAMTS7* (total score = 7) ← ←, ← ← ←<br>*WDR61* (total score = 2) ← ←<br><br>**Lempiäinen et al.** [2]**, score range 2-54**<br>*ADAMTS7* (total score = 38) ← ← ←<br>*CTSH* (total score = 8)<br><br>**van der Harst et al.** [3]	*ADAMTS7* is the causal gene.

					Adamts7$^{-/-}$/*Ldlr*$^{-/-}$ and *Adamts7*$^{-/-}$/*Apoe*$^{-/-}$ displayed significant reductions in lesion formation in aortas and aortic roots compared with control *Adamts7*$^{+/+}$ animals. Primary *Adamts7* knockout vSMCs showed reduced migration in the setting of TNF-α stimulation [114]. *Adamts7* expression was found to be down-regulated in macrophages isolated from aortas of *Ldlr*$^{-/-}$ mice fed a Western diet ("diseased" aortas) as compared to *Ldlr*$^{-/-}$ mice fed a chow diet ("healthy" aortas) [9]. Adamts7 deficiency substantially ameliorated neointima formation in mice, and in vitro studies indicated that ADAMTS7 inhibited both endothelial cell proliferation and migration [115].<br>Polymorphism rs3825807 (not in high LD with rs7178051, r^2 = 0.53 in Europeans according to LDlink, https://ldlink.nci.nih.gov/) influences ADAMTS7 maturation, thrombospondin-5 cleavage, and vSMCs migration; the variant associated with protection from atherosclerosis and CAD reduces ADAMTS7 function [113].	*ADAMTS7* ← ← ← ←<br>*RASGRF1* ← ← ← ←<br>**Svishcheva et al.** [4]<br>*ADAMTS7* (two datasets)	
44	rs17514846	15: 91 416 550	*FURIN*	▪ *FURIN* ←<br>▪ *FES* ←<br>▪ *MAN2A2* ←	*FURIN*<br>*furin, paired basic amino acid cleaving enzyme*<br>Furin is a protease that cleaves PCSK9. PCSK9 promotes atherosclerosis by increasing LDL-C levels through degradation of LDLR [116,117]. Circulating furin-cleaved PCSK9 showed an ability to regulate LDLR and serum cholesterol levels, although less efficiently than intact PCSK9 [118].<br>Besides this, Furin substrates include proparathyroid hormone, transforming growth factor beta 1 precursor (TGFB1), proalbumin, membrane type-1 matrix metalloproteinase (MT1-MMP), vWF, and the (pro)renin receptor. Soluble form of the (pro)renin receptor generated by intracellular cleavage by furin is secreted in plasma and is able to bind renin [119]. (Pro)renin receptor was suggested to have a pathological role in raising blood pressure (reviewed in [120]).	**Brænne et al.** [1]**, score range 1-11**<br>*FURIN* (total score = 8) ←<br>*FES* (total score = 7) ←<br>*MAN2A2* (total score = 3) ←<br>**Lempiäinen et al.** [2]**, score range 2-54**<br>*FURIN* (total score = 10) ←<br>*FES* (total score = 10) ←<br>**Svishcheva et al.** [4]<br>*FURIN* (two datasets)<br>*FES* (one dataset)	*FURIN* is the causal gene.<br>*FES* might also be involved.
45	rs1050362	16: 72 130 815	*DHX38*	▪ *HP* ←<br>▪ *DHX38* ←<br>▪ *DHODH* ←<br>▪ *PKD1L3* ←	*HP*<br>*haptoglobin*<br>*HP* encodes a preproprotein, which is processed to yield both alpha and beta chains that combine as a tetramer to produce haptoglobin. Haptoglobin is a moderate acute phase protein [121]. Haptoglobin binds free plasma hemoglobin (Hb) and protects tissues from oxidative damage. Plasma haptoglobin	**Svishcheva et al.** [4]<br>*HPR* (one dataset)	*HP* is the most likely causal gene.

					concentrations are elevated in patients with CAD and are significantly correlated with the severity of luminal stenosis [122].<br>The release of Hb from extravasated erythrocytes at the site of hemorrhage leads to iron deposition, which may increase oxidation and inflammation in the atherosclerotic plaque. Iron level, lipid peroxidation and macrophage accumulation was shown to be increased in atherosclerotic plaques from *Apoe*$^{-/-}$ mice bearing CAD-associated Hp alleles as compared to plaques from *Apoe*$^{-/-}$ mice with normal Hp [123].		
46	rs170041	17: 2 170 216	*SMG6*	-	*SMG6*<br>*SMG6 nonsense mediated mRNA decay factor*<br>*Smg6* expression was found to be up-regulated in pre-lesion endothelial cells and heterogeneous cells from atherosclerotic lesions isolated from aortas of *Apoe*$^{-/-}$ mice ("diseased" aortas) as compared to wild type mice ("healthy" aortas) [9].<br><br>*SRR*<br>*serine racemase*<br>*Srr* expression was found to be up-regulated in pre-lesion endothelial cells isolated from aortas of *Apoe*$^{-/-}$ mice ("diseased" aortas) as compared to wild type mice ("healthy" aortas) [9].	**Lempiäinen et al.** [2]**, score range 2-54**<br>*SRR* (total score = 8)<br><br>**Svishcheva et al.** [4]<br>*SMG6* (two datasets)	Evidence is inconsistent.
47	rs12936587	17: 17 543 722	*RAI1*	▪ *SREBF1 (SREBP1)* ←<br>▪ *PEMT* ←	*PEMT*<br>*phosphatidylethanolamine N-methyltransferase*<br>PEMT converts phosphatidylethanolamine to phosphatidylcholine by sequential methylation in the liver. Phosphatidylcholine is a major and essential component of VLDL. In *Apoe*$^{-/-}$ mice which have been fed a chow diet for 1 year, Pemt deficiency (*Pemt*$^{-/-}$) significantly improved the atherogenic lipoprotein profile of plasma (with lower levels of triacylglycerol and cholesterol in the VLDL and LDL/IDL fractions, respectively), reduced atherosclerotic lesion area, aortic cholesteryl ester and cholesterol content, and improved systolic function as compared to *Pemt*$^{+/+}$/*Apoe*$^{-/-}$ mice [124].<br><br>*SREBF1 (SREBP1)*<br>*sterol regulatory element binding transcription factor 1*<br>Transcription factor SREBF-1 regulates the expression of different genes, including the genes involved in the synthesis of fatty acids, triglycerides, and cholesterol (in particular, *LDLR*)	**Lempiäinen et al.** [2]**, score range 2-54**<br>*SREBF1* (total score = 40) ←<br>*PEMT* (total score = 40) ←	Evidence is inconsistent. *PEMT*, *SREBF1*, and *MIR33B* can be involved.

					[125,126]. SREBF-1 has two isoforms, SREBP-1a and SREBP-1c, which arise due to transcription from two alternative start sites. Hepatic activation of AMP-activated protein kinase (Ampk) was shown to protect against hepatic steatosis, hyperlipidemia, and accelerated atherosclerosis in diet-induced insulin-resistant *Ldlr*$^{-/-}$ mice in part through phosphorylation of Srebp-1c and suppression of Srebp-1c-dependent lipogenesis [127]. Inhibition of SREBP by betulin was found to improve hyperlipidemia and insulin resistance and reduce atherosclerotic plaques [128].<br>SREBP-1 was shown to suppress VEGF expression in human vSMCs [129]. VEGF was reported to accelerate atherosclerotic progression [105-107].		
					MIR33B (hsa-mir-33b)<br>*microRNA 33b*<br>*MIR33B* is located in the intron 17 of the *SREBF1* gene. MicroRNA-33 (miR-33a/b) has been discovered as a key regulator in the initiation and progression of atherosclerosis (reviewed in [130]). MicroRNA-33 regulates cholesterol/lipid homeostasis in cooperation with the SREBP host genes, participates in inflammatory response and influences insulin signaling and glucose/energy homeostasis, cell cycle progression and proliferation, and myeloid cell differentiation [130]. MiR-33b is possibly utilized for a feedback mechanism to regulate its host gene *SREBF1* [131]. Experiments in miR-33b knock-in humanized mice showed that *SREBF1*-miR-33b axis has a critical role in both lipid profiles and macrophage phenotype remodeling, and miR-33b was suggested as a promising target for treating atherosclerosis [132].		
48	rs2070783	17: 62 406 971	*PECAM1*	▪ *PECAM1* ←	*PECAM1*<br>*platelet and endothelial cell adhesion molecule 1*<br>PECAM-1 is found on the surface of platelets, monocytes, neutrophils, and some types of T-cells, and makes up a large portion of endothelial cell intercellular junctions. PECAM-1 has been implicated in the maintenance of vascular barrier integrity, a breach of which is a sign of inflammatory response. Failure to restore barrier function in a timely manner can contribute to the development of chronic inflammatory diseases such as atherosclerosis (reviewed in [133]). A study in	**Brænne et al.** [1]**, score range 1-11**<br>*PECAM1* (total score = 4) ←<br>*POLG2* (total score = 3) ←	*PECAM1* is the causal gene.

					Pecam-1 knockout mice proposed PECAM-1 to play an important role in the ability of the endothelial cells to sense and couple high temporal gradients of wall shear stress to NO-mediated arteriolar dilation during sudden changes in blood flow [134].		
49	rs12052058	19: 11 159 525	*SMARCA4*	▪ *SMARCA4* ←<br>▪ *CARM1* ←<br>▪ *C19ORF52* ←<br>▪ *KANK2*	***LDLR***<br>*low density lipoprotein receptor*<br>LDLR is a key receptor for maintaining cholesterol homeostasis. LDLR is a cell membrane glycoprotein that binds and internalizes circulating cholesterol-containing lipoprotein particles. LDLR-mediated endocytosis is essential for lipoprotein and lipid metabolism. Defects in LDLR function or expression trigger elevated LDL cholesterol and results in major atherosclerotic diseases (reviewed in [135]). In particular, mutations in the coding sequence of the *LDLR* gene was shown to co-segregate within families with premature myocardial infarction/family history for CAD [136]. Exome sequencing identified rare *LDLR* alleles conferring risk for myocardial infarction [137].<br>*LDLR* was found to be one of the top key regulators of CAD in the study based on integrative genomics approach [82].	**Brænne et al.** [1]**, score range 1-11**<br>*KANK2* (total score = 5) ← ←<br>*SMARCA4* (total score = 4) ←<br>*ANKRD25* (total score = 2) ← ←<br><br>**Lempiäinen et al.** [2]**, score range 2-54**<br>*LDLR* (total score = 35) ←<br>*CARM1* (total score = 40) ←**,** ← ← ←<br>*SMARCA4* (total score = 40) ←**,** ← ← ←<br>*C19ORF38* (total score = 10) ← ← ←<br><br>**Svishcheva et al.** [4]<br>*LDLR* (two datasets)<br>*SMARCA4* (one dataset)	*LDLR* is the causal gene.<br><br>*SMARCA4* and *CARM1* might also be involved.
					SMARCA4<br>*SWI/SNF related, matrix associated, actin dependent regulator of chromatin, subfamily a, member 4*<br>SMARCA4 was shown to be involved in vascular calcification [138]. Calcification of the coronary arteries plays a key role in the pathophysiology of atherosclerosis. The presence of coronary calcification is a surrogate marker of the overall plaque burden [19].		
					CARM1<br>*coactivator associated arginine methyltransferase 1*<br>C9orf72–CARM1 axis was shown to regulate the lipid metabolism in the cell, particularly under conditions of glucose starvation stress. C9orf72 promotes the lysosomal degradation of its interactor CARM1, and CARM1 regulates autophagy–lysosomal functions and lipid metabolism (at a transcriptional level) [139].		

50	rs867186	20: 33 764 554	*PROCR, MMP24-AS1-EDEM2*	▪ *TRPC4AP* ←<br>▪ *EIF6* ←<br>▪ *ITGB4BP* ←<br>▪ *EDEM2* ← ←<br>▪ *HS.443185* ← ←	*PROCR (EPCR)*<br>*protein C receptor*<br>PROCR is a serine protease activated by and involved in the blood coagulation pathway. PROCR enhances the activation of protein C. The protein C system is now emerging as a novel participant in the pathogenesis of acute and chronic inflammatory diseases, such as sepsis, asthma, inflammatory bowel disease, atherosclerosis, and lung and heart inflammation (reviewed in [140]).	**Brænne et al.** [1]**, score range 1-11**<br>*PROCR* (total score = 8) ←<br>*MYH7B* (total score = 5) ←<br>*TRPC4AP* (total score = 3) ←<br>*EIF6* (total score = 3) ←<br>*RBL1* (total score = 3) ←<br>*ROMO1* (total score = 2) ←<br>*ITGB4BP* (total score = 2) ←<br>*FLJ25841* (total score = 1) ←<br>*MT1P3* (total score = 1) ←<br><br>**van der Harst et al.** [3]<br>*PROCR*¶ ←<br>*TRPC4AP*¶ ←<br>*GGT7* ←<br>*EDEM2* ←<br>*NCOA6* ←<br>*HMGB3P1* ←	*PROCR* is the most likely causal gene.
51	rs9982601	21: 35 599 128	*LINC00310*	▪ *MRPS6*<br>▪ *KCNE2*	*KCNE2 (MIRP1)*<br>*potassium voltage-gated channel subfamily E regulatory subunit 2*<br>KCNE2 is a small integral membrane subunit that assembles with the KCNH2 gene product, a pore-forming protein, to alter its function. *KCNE2* (MiRP1) is expressed in heart and muscle. Mutations in *KCNE2* cause inherited cardiac arrhythmias. Germline *Kcne2* deletion promotes atherosclerosis in mice. In female western diet-fed mice, *Kcne2* deletion increased plaque deposition > 6-fold and also caused premature ventricular complexes and sudden death [141].	**Lempiäinen et al.** [2]**, score range 2-54**<br>*SON* (total score = 8)<br><br>**van der Harst et al.** [3]<br>*MRPS6*¶ ←<br>*SLC5A3*¶ ←	*KCNE2* is the most likely causal gene.

¥ Loci for the analysis in our study were defined as regions within ±250 kb around these lead SNPs (see Supplementary Table S1c)

* Chromosome: position of the lead SNP on the chromosome according to GRCh37.p13

† Nearest gene according to the NCBI dbSNP database (https://www.ncbi.nlm.nih.gov/snp/)

‡ Information on whether increased gene expression in CAD-relevant tissue is associated with the increased or decreased CAD risk is given in Supplementary Table S2a

¶ Converging evidence of a potential functional SNP-gene mechanism (demonstrated in the study by van der Harst et al. [3]).

#, § These pairs of loci are overlapping and contain partially the same genes. Since the distance between the lead SNPs rs3103349–rs10455872 and rs3184504–rs441 was > 250 kb (269,4 kb and 344,2 kb, respectively), SMR/HEIDI analysis was performed for each locus (±250 kb around the lead SNP) separately. The genes prioritized based on literature data and revealed in the gene-based association analysis [4], if located in two loci in the pair, were attributed to both. Similarly, if the CAD-associated SNPs prioritized in the studies by Brænne et al. [1], Lempiäinen et al. [2], and van der Harst et al. [3] were located in two loci in the pair, we attributed the genes linked with these SNPs to both loci.

CAD, coronary artery disease; GWAS, genome-wide association study; IDL, intermediate-density lipoprotein; LD, linkage disequilibrium; LDL, low-density lipoprotein; LDLR, LDL receptor; LDL-C, low-density lipoprotein cholesterol; MMP, matrix metalloproteinase; ROS, reactive oxygen species; SMC, smooth muscle cell; VLDL, very-low-density lipoprotein; vWF, von Willebrand factor; vSMC, vascular smooth muscle cell.

REFERENCES

1. Brænne I, Civelek M, Vilne B, Di Narzo A, Johnson AD, Zhao Y, et al. Prediction of causal candidate genes in coronary artery disease loci. Arterioscler Thromb Vasc Biol. 2015;35: 2207–2217. doi:10.1161/ATVBAHA.115.306108

2. Lempiäinen H, Brænne I, Michoel T, Tragante V, Vilne B, Webb TR, et al. Network analysis of coronary artery disease risk genes elucidates disease mechanisms and druggable targets. Sci Rep. 2018;8: 3434. doi:10.1038/s41598-018-20721-6

3. van der Harst P, Verweij N. Identification of 64 novel genetic loci provides an expanded view on the genetic architecture of coronary artery disease. Circ Res. 2018;122: 433–443. doi:10.1161/CIRCRESAHA.117.312086

4. Svishcheva GR, Belonogova NM, Zorkoltseva I V, Kirichenko A V, Axenovich TI. Gene-based association tests using GWAS summary statistics. Bioinformatics. 2019;btz172. doi:10.1093/bioinformatics/btz172

5. Wu C, Huang R-T, Kuo C-H, Kumar S, Kim CW, Lin Y-C, et al. Mechanosensitive PPAP2B regulates endothelial responses to atherorelevant hemodynamic forces. Circ Res. 2015;117. doi:10.1161/CIRCRESAHA.117.306457

6. Schober A, Siess W. Lysophosphatidic acid in atherosclerotic diseases. Br J Pharmacol. 2012;167: 465–482. doi:10.1111/j.1476-5381.2012.02021.x

7. Zhou Z, Subramanian P, Sevilmis G, Globke B, Soehnlein O, Karshovska E, et al. Lipoprotein-derived lysophosphatidic acid promotes atherosclerosis by releasing CXCL1 from the endothelium. Cell Metab. 2011;13: 592–600. doi:10.1016/J.CMET.2011.02.016

8. Busnelli M, Manzini S, Hilvo M, Parolini C, Ganzetti GS, Dellera F, et al. Liver-specific deletion of the Plpp3 gene alters plasma lipid composition and worsens atherosclerosis in apoE−/− mice. Sci Rep. 2017;7: 44503. doi:10.1038/srep44503

9. Erbilgin A, Civelek M, Romanoski CE, Pan C, Hagopian R, Berliner JA, et al. Identification of CAD candidate genes in GWAS loci and their expression in vascular cells. J Lipid Res. 2013;54: 1894–1905. doi:10.1194/jlr.M037085

10. Musunuru K, Strong A, Frank-Kamenetsky M, Lee NE, Ahfeldt T, Sachs K V., et al. From noncoding variant to phenotype via SORT1 at the 1p13 cholesterol locus. Nature. 2010;466: 714–719. doi:10.1038/nature09266

11. Strong A, Ding Q, Edmondson AC, Millar JS, Sachs K V., Li X, et al. Hepatic sortilin regulates both apolipoprotein B secretion and LDL catabolism. J Clin Invest. 2012;122: 2807–2816. doi:10.1172/JCI63563

12. Gustafsen C, Kjolby M, Nyegaard M, Mattheisen M, Lundhede J, Buttenschøn H, et al. The hypercholesterolemia-risk gene SORT1 facilitates PCSK9 secretion. Cell Metab. 2014;19: 310–318. doi:10.1016/j.cmet.2013.12.006

13. Kjolby M, Nielsen MS, Petersen CM. Sortilin, Encoded by the cardiovascular risk gene SORT1, and its suggested functions in cardiovascular disease. Curr Atheroscler Rep. 2015;17: 18. doi:10.1007/s11883-015-0496-7

14. Strong A, Patel K, Rader DJ. Sortilin and lipoprotein metabolism. Curr Opin Lipidol. 2014;25: 350–357. doi:10.1097/MOL.0000000000000110

15. Guo K, Hu L, Xi D, Zhao J, Liu J, Luo T, et al. PSRC1 overexpression attenuates atherosclerosis progression in apoE-/- mice by modulating cholesterol transportation and inflammation. J Mol Cell Cardiol. 2018;116: 69–80. doi:10.1016/j.yjmcc.2018.01.013

16. Abeywardena MY, Leifert WR, Warnes KE, Varghese JN, Head RJ. Cardiovascular biology of interleukin-6. Curr Pharm Des. 2009;15: 1809–1821. doi:10.2174/138161209788186290

17. IL6R Genetics Consortium Emerging Risk Factors Collaboration, Sarwar N, Butterworth AS, Freitag DF, Gregson J, Willeit P, et al. Interleukin-6 receptor pathways in coronary heart disease: a collaborative meta-analysis of 82 studies. Lancet. 2012;379: 1205–1213. doi:10.1016/S0140-6736(11)61931-4

18. Jiang ZM, Wu XJ, Liu Y, Du XH, Shen SJ, Xu LY, et al. Changes of gene expression profiles across different phases of vascular calcification in rats. Genet Mol Res. 2013;12: 5945–5957. doi:10.4238/2013.November.26.4

19. Dharampal AS, de Feyter PJ. Coronary artery calcification: does it predict obstructive coronary artery disease? Neth Heart J. 2013;21: 344–346. doi:10.1007/s12471-013-0436-5

20. Van Assche T, Hendrickx J, Crauwels HM, Guns P-J, Martinet W, Fransen P, et al. Transcription profiles of aortic smooth muscle cells from atherosclerosis-prone and -resistant regions in young apolipoprotein E-deficient mice before plaque development. J Vasc Res. 2011;48: 31–42. doi:10.1159/000317398

21. Carmosino M, Torretta S, Procino G, Timperio A, Zolla L, Svelto M. Na+/K+-ATPase β1-subunit is recruited in Na-K-2Cl co-transporter isoform 2 multiprotein complexes in rat kidneys. J Hypertens. 2014;32: 1842–1853. doi:10.1097/HJH.0000000000000258

22. Ferrandi M, Tripodi G, Salardi S, Florio M, Modica R, Barassi P, et al. Renal Na,K-ATPase in genetic hypertension. Hypertension. 1996;28: 1018–1025. doi:10.1161/01.hyp.28.6.1018

23. Perisic Matic L, Rykaczewska U, Razuvaev A, Sabater-Lleal M, Lengquist M, Miller CL, et al. Phenotypic modulation of smooth muscle cells in atherosclerosis is associated with downregulation of *LMOD1*, *SYNPO2*, *PDLIM7*, *PLN* , and *SYNM*. Arterioscler Thromb Vasc Biol. 2016;36: 1947–1961. doi:10.1161/ATVBAHA.116.307893

24. Benn M. Apolipoprotein B levels, APOB alleles, and risk of ischemic cardiovascular disease in the general population, a review. Atherosclerosis. 2009;206: 17–30. doi:10.1016/J.ATHEROSCLEROSIS.2009.01.004

25. Chen Z, Fitzgerald RL, Averna MR, Schonfeld G. A targeted apolipoprotein B-38.9-producing mutation causes fatty livers in mice due to the reduced ability of apolipoprotein B-38.9 to transport triglycerides. J Biol Chem. 2000;275: 32807–32815. doi:10.1074/jbc.M004913200

26. Yu X-H, Qian K, Jiang N, Zheng X-L, Cayabyab FS, Tang C-K. ABCG5/ABCG8 in cholesterol excretion and atherosclerosis. Clin Chim Acta. 2014;428: 82–88. doi:10.1016/j.cca.2013.11.010

27. Helgadottir A, Alexandersson KF, Thorgeirsson G, Olafsson I, Sigurdardottir O, Eyjolfsson G, et al. Rare missense mutations of ABCG5/ABCG8 raise cholesterol and phytosterol levels and increase the risk of coronary artery disease. Circulation. 2016;134: Suppl1, A19235.

28. Danziger J. Vitamin K-dependent proteins, warfarin, and vascular calcification. Clin J Am Soc Nephrol. 2008;3: 1504–1510. doi:10.2215/CJN.00770208

29. Ren Q, Barber HK, Crawford GL, Karim ZA, Zhao C, Choi W, et al. Endobrevin/VAMP-8 Is the primary v-SNARE for the platelet release reaction. Mol Biol Cell. 2007;18: 24–33. doi:10.1091/mbc.e06-09-0785

30. Baumgartner HR, Hosang M. Platelets, platelet-derived growth factor and arteriosclerosis. Experientia. 1988;44: 109–112. doi:10.1007/bf01952191

31. Cunnington RH, Northcott JM, Ghavami S, Filomeno KL, Jahan F, Kavosh MS, et al. The Ski-Zeb2-Meox2 pathway provides a novel mechanism for regulation of the cardiac myofibroblast phenotype. J Cell Sci. 2014;127: 40–49. doi:10.1242/jcs.126722

32. Moilanen A-M, Rysä J, Kaikkonen L, Karvonen T, Mustonen E, Serpi R, et al. WDR12, a member of nucleolar PeBoW-complex, is up-regulated in failing hearts and causes deterioration of cardiac function. PLoS One. 2015;10: e0124907. doi:10.1371/journal.pone.0124907

33. Erdmann J, Großhennig A, Braund PS, König IR, Hengstenberg C, Hall AS, et al. New susceptibility locus for coronary artery disease on chromosome 3q22.3. Nat Genet. 2009;41: 280–282. doi:10.1038/ng.307

34. Yoshikawa Y, Satoh T, Tamura T, Wei P, Bilasy SE, Edamatsu H, et al. The M-Ras-RA-GEF-2-Rap1 pathway mediates tumor necrosis factor-α–dependent regulation of integrin activation in splenocytes. Mol Biol Cell. 2007;18: 2949–2959. doi:10.1091/mbc.e07-03-0250

35. Galkina E, Ley K. Vascular adhesion molecules in atherosclerosis. Arterioscler Thromb Vasc Biol. 2007;27: 2292–2301. doi:10.1161/ATVBAHA.107.149179

36. Zygalaki E, Kaklamanis L, Lolaka M, Nikolaou N, Koutouzis M, Lianidou ES, et al. Systemic hypertension augments, whereas insulin-dependent diabetes down-regulates, endothelin A receptor expression in the mammary artery in coronary artery disease patients. Cardiol J. 2009;16: 348–354.

37. Avedanian L, Riopel J, Bkaily G, Nader M, D'Orleans-Juste P, Jacques D. ETA receptors are present in human aortic vascular endothelial cells and modulate intracellular calcium. J Physiol Pharmacol. 2010;88: 817–829. doi:10.1139/Y10-057

38. Ballinger ML, Ivey ME, Osman N, Thomas WG, Little PJ. Endothelin-1 activates ETA receptors on human vascular smooth muscle cells to yield proteoglycans with increased binding to LDL. Atherosclerosis. 2009;205: 451–457. doi:10.1016/j.atherosclerosis.2009.01.024

39. Erdmann J, Stark K, Esslinger UB, Rumpf PM, Koesling D, de Wit C, et al. Dysfunctional nitric oxide signalling increases risk of myocardial infarction. Nature. 2013;504: 432–436. doi:10.1038/nature12722

40. Kessler T, Wobst J, Wolf B, Eckhold J, Vilne B, Hollstein R, et al. Functional characterization of the *GUCY1A3* coronary artery disease risk locus. Circulation. 2017;136: 476–489. doi:10.1161/CIRCULATIONAHA.116.024152

41. Chen L, Qian H, Luo Z, Li D, Xu H, Chen J, et al. PHACTR1 gene polymorphism with the risk of coronary artery disease in Chinese Han population. Postgrad Med J. 2019;95: 67–71. doi:10.1136/postgradmedj-2018-136298

42. Paquette M, Dufour R, Baass A. PHACTR1 genotype predicts coronary artery disease in patients with familial hypercholesterolemia. J Clin Lipidol. 2018;12: 966–971. doi:10.1016/j.jacl.2018.04.012

43. Reschen ME, Lin D, Chalisey A, Soilleux EJ, O'Callaghan CA. Genetic and environmental risk factors for atherosclerosis regulate transcription of phosphatase and actin regulating gene PHACTR1. Atherosclerosis. Elsevier; 2016;250: 95–105. doi:10.1016/j.atherosclerosis.2016.04.025

44. Sazonova O, Zhao Y, Nürnberg S, Miller C, Pjanic M, Castano VG, et al. Characterization of TCF21 downstream target regions identifies a transcriptional network linking multiple independent coronary artery disease loci. PLOS Genet. 2015;11: e1005202. doi:10.1371/journal.pgen.1005202

45. Emerging Risk Factors Collaboration, Erqou S, Kaptoge S, Perry PL, Di Angelantonio E, Thompson A, et al. Lipoprotein(a) concentration and the risk of coronary heart disease, stroke, and nonvascular mortality. JAMA. 2009;302: 412. doi:10.1001/jama.2009.1063

46. Nordestgaard BG, Chapman MJ, Ray K, Borén J, Andreotti F, Watts GF, et al. Lipoprotein(a) as a cardiovascular risk factor: current status. Eur Heart J. 2010;31: 2844–2853. doi:10.1093/eurheartj/ehq386

47. Emdin CA, Khera A V, Natarajan P, Klarin D, Won H-H, Peloso GM, et al. Phenotypic characterization of genetically lowered human lipoprotein(a) levels. J Am Coll Cardiol. 2016;68: 2761–2772. doi:10.1016/j.jacc.2016.10.033

48. Sekine Y, Hatanaka R, Watanabe T, Sono N, Iemura S, Natsume T, et al. The Kelch repeat protein KLHDC10 regulates oxidative stress-induced ASK1 activation by suppressing PP5. Mol Cell. 2012;48: 692–704. doi:10.1016/j.molcel.2012.09.018

49. Yamaguchi N, Sekine S, Naguro I, Sekine Y, Ichijo H. KLHDC10 deficiency protects mice against TNFα-induced systemic inflammation. PLoS One. 2016;11: e0163118. doi:10.1371/journal.pone.0163118

50. Jones PD, Kaiser MA, Ghaderi Najafabadi M, McVey DG, Beveridge AJ, Schofield CL, et al. The coronary artery disease-associated coding variant in zinc finger C3HC-type containing 1 (ZC3HC1) affects cell cycle regulation. J Biol Chem. 2016;291: 16318–16327. doi:10.1074/jbc.M116.734020

51. Boehm M, Nabel EG. The cell cycle and cardiovascular diseases. Prog Cell Cycle Res. 2003;5: 19–30.

52. Sivapalaratnam S, Maiwald S, Motazacker M, Albers C, Sambrook J, Levi M, et al. Rare mutations in MCF2L and ZC3HC1 are associated with premature atherosclerosis. The molecular basis of early onset cardiovascular disease. PhD thesis. Chapter 11. 2012. Available: https://pure.uva.nl/ws/files/1645445/109898_15b.pdf

53. Sellers MM, Stallone JN. Sympathy for the devil: the role of thromboxane in the regulation of vascular tone and blood pressure. Am J Physiol Circ Physiol. 2008;294: H1978–H1986. doi:10.1152/ajpheart.01318.2007

54. Ramazi S, Heydari-Zarnagh H, Goudarzian M, Khalaj-Kondori M, Bonyadi M. Thromboxane A synthase 1 gene expression and promotor haplotypes are associated with risk of large artery-atherosclerosis stroke in Iranian population. J Cell Biochem. 2019; jcb.28787. doi:10.1002/jcb.28787

55. Khera A V., Kathiresan S. Genetics of coronary artery disease: discovery, biology and clinical translation. Nat Rev Genet. 2017;18: 331–344. doi:10.1038/nrg.2016.160

56. Iwamoto S, Boonvisut S, Makishima S, Ishizuka Y, Watanabe K, Nakayama K. The role of TRIB1 in lipid metabolism; from genetics to pathways. Biochem Soc Trans. 2015;43: 1063–1068. doi:10.1042/BST20150094

57. Broadbent HM, Peden JF, Lorkowski S, Goel A, Ongen H, Green F, et al. Susceptibility to coronary artery disease and diabetes is encoded by distinct, tightly linked SNPs in the ANRIL locus on

chromosome 9p. Hum Mol Genet. 2008;17: 806–814. doi:10.1093/hmg/ddm352

58. Congrains A, Kamide K, Katsuya T, Yasuda O, Oguro R, Yamamoto K, et al. CVD-associated non-coding RNA, ANRIL, modulates expression of atherogenic pathways in VSMC. Biochem Biophys Res Commun. 2012;419: 612–616. doi:10.1016/j.bbrc.2012.02.050

59. Jarinova O, Stewart AFR, Roberts R, Wells G, Lau P, Naing T, et al. Functional analysis of the chromosome 9p21.3 coronary artery disease risk locus. Arterioscler Thromb Vasc Biol. 2009;29: 1671–1677. doi:10.1161/ATVBAHA.109.189522

60. Congrains A, Kamide K, Oguro R, Yasuda O, Miyata K, Yamamoto E, et al. Genetic variants at the 9p21 locus contribute to atherosclerosis through modulation of ANRIL and CDKN2A/B. Atherosclerosis. 2012;220: 449–455. doi:10.1016/j.atherosclerosis.2011.11.017

61. Motterle A, Pu X, Wood H, Xiao Q, Gor S, Liang Ng F, et al. Functional analyses of coronary artery disease associated variation on chromosome 9p21 in vascular smooth muscle cells. Hum Mol Genet. 2012;21: 4021–4029. doi:10.1093/hmg/dds224

62. Holdt LM, Beutner F, Scholz M, Gielen S, Gäbel G, Bergert H, et al. *ANRIL* expression is associated with atherosclerosis risk at chromosome 9p21. Arterioscler Thromb Vasc Biol. 2010;30: 620–627. doi:10.1161/ATVBAHA.109.196832

63. Holdt LM, Stahringer A, Sass K, Pichler G, Kulak NA, Wilfert W, et al. Circular non-coding RNA ANRIL modulates ribosomal RNA maturation and atherosclerosis in humans. Nat Commun. 2016;7. doi:10.1038/ncomms12429

64. Kojima Y, Downing K, Kundu R, Miller C, Dewey F, Lancero H, et al. Cyclin-dependent kinase inhibitor 2B regulates efferocytosis and atherosclerosis. J Clin Invest. 2019;124: 1083–1097. doi:10.1172/JCI70391

65. Visel A, Zhu Y, May D, Afzal V, Gong E, Attanasio C, et al. Targeted deletion of the 9p21 non-coding coronary artery disease risk interval in mice. Nature. 2010;464: 409–412. doi:10.1038/nature08801

66. Zhang H, Mooney CJ, Reilly MP. ABO Blood groups and cardiovascular diseases. Int J Vasc Med. 2012;2012: 1–11. doi:10.1155/2012/641917

67. Kawecki C, Lenting PJ, Denis C V. von Willebrand factor and inflammation. J Thromb Haemost. 2017;15: 1285–1294. doi:10.1111/jth.13696

68. Chen Z, Yang S-H, Xu H, Li J-J. ABO blood group system and the coronary artery disease: an updated systematic review and meta-analysis. Sci Rep. 2016;6: 23250. doi:10.1038/srep23250

69. Chen Y, Chen C, Ke X, Xiong L, Shi Y, Li J, et al. Analysis of circulating cholesterol levels as a mediator of an association between ABO blood group and coronary heart disease. Circ Cardiovasc Genet. 2014;7: 43–48. doi:10.1161/CIRCGENETICS.113.000299

70. Lennie Wong F, Kodama K, Sasaki H, Yamada M, Hamilton HB. Longitudinal study of the association between ABO phenotype and total serum cholesterol level in a Japanese cohort. Genet Epidemiol. 1992;9: 405–418. doi:10.1002/gepi.1370090604

71. Chauhan AK, Motto DG, Lamb CB, Bergmeier W, Dockal M, Plaimauer B, et al. Systemic antithrombotic effects of ADAMTS13. J Exp Med. 2006;203: 767–76. doi:10.1084/jem.20051732

72. Gandhi C, Khan MM, Lentz SR, Chauhan AK. ADAMTS13 reduces vascular inflammation and the development of early atherosclerosis in mice. Blood. 2012;119: 2385–23891. doi:10.1182/blood-2011-09-376202

73. De Meyer SF, Savchenko AS, Haas MS, Schatzberg D, Carroll MC, Schiviz A, et al. Protective anti-inflammatory effect of ADAMTS13 on myocardial ischemia/reperfusion injury in mice. Blood. 2012;120: 5217–5123. doi:10.1182/blood-2012-06-439935

74. Xu H, Cao Y, Yang X, Cai P, Kang L, Zhu X, et al. ADAMTS13 controls vascular remodeling by modifying VWF reactivity during stroke recovery. Blood. 2017;130: 11–22. doi:10.1182/blood-2016-10-747089

75. Kaikita K, Soejima K, Matsukawa M, Nakagaki T, Ogawa H. Reduced von Willebrand factor-cleaving protease (ADAMTS13) activity in acute myocardial infarction. J Thromb Haemost. 2006;4: 2490–2493. doi:10.1111/j.1538-7836.2006.02161.x

76. Matsukawa M, Kaikita K, Soejima K, Fuchigami S, Nakamura Y, Honda T, et al. Serial changes in von Willebrand factor-cleaving protease (ADAMTS13) and prognosis after acute myocardial

infarction. Am J Cardiol. 2007;100: 758–763. doi:10.1016/J.AMJCARD.2007.03.095

77. Hara T, Monguchi T, Iwamoto N, Akashi M, Mori K, Oshita T, et al. Targeted disruption of JCAD (Junctional Protein Associated With Coronary Artery Disease)/KIAA1462, a coronary artery disease–associated gene product, inhibits angiogenic processes in vitro and in vivo. Arterioscler Thromb Vasc Biol. 2017;37: 1667–1673. doi:10.1161/ATVBAHA.117.309721

78. Jones PD, Kaiser MA, Ghaderi Najafabadi M, Koplev S, Zhao Y, Douglas G, et al. *JCAD*, a gene at the 10p11 coronary artery disease locus, regulates Hippo signaling in endothelial cells. Arterioscler Thromb Vasc Biol. 2018;38: 1711–1722. doi:10.1161/ATVBAHA.118.310976

79. Zernecke A, Shagdarsuren E, Weber C. Chemokines in atherosclerosis. Arterioscler Thromb Vasc Biol. 2008;28: 1897–1908. doi:10.1161/ATVBAHA.107.161174

80. Damås JK, Waehre T, Yndestad A, Ueland T, Müller F, Eiken HG, et al. Stromal cell-derived factor-1alpha in unstable angina: potential antiinflammatory and matrix-stabilizing effects. Circulation. 2002;106: 36–42. doi:10.1161/01.cir.0000020001.09990.90

81. Zernecke A, Bidzhekov K, Noels H, Shagdarsuren E, Gan L, Denecke B, et al. Delivery of microRNA-126 by apoptotic bodies induces CXCL12-dependent vascular protection. Sci Signal. 2009;2: ra81–ra81. doi:10.1126/scisignal.2000610

82. Zhao Y, Chen J, Freudenberg JM, Meng Q, Rajpal DK, Yang X. Network-based identification and prioritization of key regulators of coronary artery disease loci. Arterioscler Thromb Vasc Biol. 2016;36: 928–941. doi:10.1161/ATVBAHA.115.306725

83. Morris GE, Braund PS, Moore JS, Samani NJ, Codd V, Webb TR. Coronary artery disease–associated *LIPA* coding variant rs1051338 reduces lysosomal acid lipase levels and activity in lysosomes. Arterioscler Thromb Vasc Biol. 2017;37: 1050–1057. doi:10.1161/ATVBAHA.116.308734

84. Diver LA, MacKenzie SM, Fraser R, McManus F, Freel EM, Alvarez-Madrazo S, et al. Common polymorphisms at the *CYP17A1* locus associate with steroid phenotype. Hypertension. 2016;67: 724–732. doi:10.1161/HYPERTENSIONAHA.115.06925

85. Xanthakis V, Vasan RS. Aldosterone and the risk of hypertension. Curr Hypertens Rep. 2013;15: 102–7. doi:10.1007/s11906-013-0330-y

86. Weber T, Lang I, Zweiker R, Horn S, Wenzel RR, Watschinger B, et al. Hypertension and coronary artery disease: epidemiology, physiology, effects of treatment, and recommendations. Wien Klin Wochenschr. 2016;128: 467–479. doi:10.1007/s00508-016-0998-5

87. Lawrence T. The nuclear factor NF-kappaB pathway in inflammation. Cold Spring Harb Perspect Biol. 2009;1: a001651. doi:10.1101/cshperspect.a001651

88. Lee C. Platelet-derived growth factor-C and -D in the cardiovascular system and diseases. Mol Aspects Med. 2018;62: 12–21. doi:10.1016/J.MAM.2017.09.005

89. Wågsäter D, Zhu C, Björck HM, Eriksson P. Effects of PDGF-C and PDGF-D on monocyte migration and MMP-2 and MMP-9 expression. Atherosclerosis. 2009;202: 415–423. doi:10.1016/j.atherosclerosis.2008.04.050

90. Pontén A, Bergsten Folestad E, Pietras K, Eriksson U. Platelet-derived growth factor D induces cardiac fibrosis and proliferation of vascular smooth muscle cells in heart-specific transgenic Mice. Circ Res. 2005;97: 1036–1045. doi:10.1161/01.RES.0000190590.31545.d4

91. Devallière J, Charreau B. The adaptor Lnk (SH2B3): An emerging regulator in vascular cells and a link between immune and inflammatory signaling. Biochem Pharmacol. 2011;82: 1391–1402. doi:10.1016/j.bcp.2011.06.023

92. Wang W, Tang Y, Wang Y, Tascau L, Balcerek J, Tong W, et al. LNK/SH2B3 loss of function promotes atherosclerosis and thrombosis. Circ Res. 2016;119: e91–e103. doi:10.1161/CIRCRESAHA.116.308955

93. Lastres-Becker I, Brodesser S, Lütjohann D, Azizov M, Buchmann J, Hintermann E, et al. Insulin receptor and lipid metabolism pathology in ataxin-2 knock-out mice. Hum Mol Genet. 2008;17: 1465–1481. doi:10.1093/hmg/ddn035

94. Wenzel P, Müller J, Zurmeyer S, Schuhmacher S, Schulz E, Oelze M, et al. ALDH-2 deficiency increases cardiovascular oxidative stress---Evidence for indirect antioxidative properties. Biochem Biophys Res Commun. 2008;367: 137–143. doi:10.1016/j.bbrc.2007.12.089

95. Szöcs K, Lassègue B, Wenzel P, Wendt M, Daiber A, Oelze M, et al. Increased superoxide production in nitrate tolerance is associated with NAD(P)H oxidase and aldehyde dehydrogenase 2

downregulation. J Mol Cell Cardiol. 2007;42: 1111–1118. doi:10.1016/j.yjmcc.2007.03.904

96. Choi H, Tostes RC, Webb RC. Mitochondrial aldehyde dehydrogenase prevents ROS-induced vascular contraction in angiotensin-II hypertensive mice. J Am Soc Hypertens. 2011;5: 154–160. doi:10.1016/J.JASH.2011.02.005

97. Vichova T, Motovska Z. Oxidative stress: Predictive marker for coronary artery disease. Exp Clin Cardiol. 2013;18: e88-91.

98. Odom DT, Zizlsperger N, Gordon DB, Bell GW, Rinaldi NJ, Murray HL, et al. Control of pancreas and liver gene expression by HNF transcription factors. Science. 2004;303: 1378–1381. doi:10.1126/science.1089769

99. Fajans SS, Bell GI, Polonsky KS. Molecular mechanisms and clinical pathophysiology of maturity-onset diabetes of the young. N Engl J Med. 2001;345: 971–980. doi:10.1056/NEJMra002168

100. Armendariz AD, Krauss RM. Hepatic nuclear factor 1-alpha: inflammation, genetics, and atherosclerosis. Curr Opin Lipidol. 2009;20: 106–111. doi: 10.1097/MOL.0b013e3283295ee9

101. Voight BF, Peloso GM, Orho-Melander M, Frikke-Schmidt R, Barbalic M, Jensen MK, et al. Plasma HDL cholesterol and risk of myocardial infarction: a mendelian randomisation study. Lancet. 2012;380: 572–580. doi:10.1016/S0140-6736(12)60312-2

102. Ferrara N. Molecular and biological properties of vascular endothelial growth factor. J Mol Med (Berl). 1999;77: 527–543.

103. Ng Y-S, Krilleke D, Shima DT. VEGF function in vascular pathogenesis. Exp Cell Res. 2006;312: 527–537. doi:10.1016/j.yexcr.2005.11.008

104. Lacal PM, Graziani G. Therapeutic implication of vascular endothelial growth factor receptor-1 (VEGFR-1) targeting in cancer cells and tumor microenvironment by competitive and non-competitive inhibitors. Pharmacol Res. 2018;136: 97–107. doi:10.1016/j.phrs.2018.08.023

105. Celletti FL, Waugh JM, Amabile PG, Brendolan A, Hilfiker PR, Dake MD. Vascular endothelial growth factor enhances atherosclerotic plaque progression. Nat Med. 2001;7: 425–429. doi:10.1038/86490

106. Celletti FL, Hilfiker PR, Ghafouri P, Dake MD. Effect of human recombinant vascular endothelial growth factor165on progression of atherosclerotic plaque. J Am Coll Cardiol. 2001;37: 2126–2130. doi:10.1016/S0735-1097(01)01301-8

107. Zhao Q, Egashira K, Inoue S, Usui M, Kitamoto S, Ni W, et al. Vascular endothelial growth factor is necessary in the development of arteriosclerosis by recruiting/activating monocytes in a rat model of long-term inhibition of nitric oxide synthesis. Circulation. 2002;105: 1110–1115. doi:10.1161/hc0902.104718

108. Roncal C, Buysschaert I, Gerdes N, Georgiadou M, Ovchinnikova O, Fischer C, et al. Short-term delivery of anti-PlGF antibody delays progression of atherosclerotic plaques to vulnerable lesions. Cardiovasc Res. 2010;86: 29–36. doi:10.1093/cvr/cvp380

109. Pilarczyk K, Sattler KJE, Galili O, Versari D, Olson ML, Meyer FB, et al. Placenta growth factor expression in human atherosclerotic carotid plaques is related to plaque destabilization. Atherosclerosis. 2008;196: 333–340. doi:10.1016/j.atherosclerosis.2006.10.038

110. Turner AW, Nikpay M, Silva A, Lau P, Martinuk A, Linseman TA, et al. Functional interaction between COL4A1/COL4A2 and SMAD3 risk loci for coronary artery disease. Atherosclerosis. 2015;242: 543–552. doi:10.1016/j.atherosclerosis.2015.08.008

111. Yang W, Ng FL, Chan K, Pu X, Poston RN, Ren M, et al. Coronary-Heart-Disease-Associated Genetic Variant at the COL4A1/COL4A2 locus affects COL4A1/COL4A2 expression, vascular cell survival, atherosclerotic plaque stability and risk of myocardial infarction. PLOS Genet. 2016;12: e1006127. doi:10.1371/journal.pgen.1006127

112. Aravani D, Karamanavi E, Andrews SL, Samani NJ, Stringer EJ, Webb TR. A The coronary artery disease associated gene HHIPL1 promotes atherosclerosis. Heart. 2016;102: A145–A146. doi:10.1136/heartjnl-2016-309890.220

113. Pu X, Xiao Q, Kiechl S, Chan K, Ng FL, Gor S, et al. ADAMTS7 cleavage and vascular smooth muscle cell migration is affected by a coronary-artery-disease-associated variant. Am J Hum Genet. 2013;92: 366–374. doi:10.1016/j.ajhg.2013.01.012

114. Bauer RC, Tohyama J, Cui J, Cheng L, Yang J, Zhang X, et al. Knockout of *Adamts7*, a novel coronary artery disease locus in humans, reduces atherosclerosis in mice. Circulation. 2015;131: 1202–1213. doi:10.1161/CIRCULATIONAHA.114.012669

115. Kessler T, Zhang L, Liu Z, Yin X, Huang Y, Wang Y, et al. ADAMTS-7 Inhibits re-endothelialization of injured arteries and promotes vascular remodeling through cleavage of tThrombospondin-1. Circulation. 2015;131: 1191–1201. doi:10.1161/CIRCULATIONAHA.114.014072

116. Cariou B, Le May C, Costet P. Clinical aspects of PCSK9. Atherosclerosis. 2011;216: 258–265. doi:10.1016/J.ATHEROSCLEROSIS.2011.04.018

117. Giunzioni I, Tavori H, Covarrubias R, Major AS, Ding L, Zhang Y, et al. Local effects of human PCSK9 on the atherosclerotic lesion. J Pathol. 2016;238: 52–62. doi:10.1002/path.4630

118. Lipari MT, Li W, Moran P, Kong-Beltran M, Sai T, Lai J, et al. Furin-cleaved proprotein convertase subtilisin/kexin type 9 (PCSK9) is active and modulates low density lipoprotein receptor and serum cholesterol levels. J Biol Chem. 2012;287: 43482–43491. doi:10.1074/jbc.M112.380618

119. Cousin C, Bracquart D, Contrepas A, Corvol P, Muller L, Nguyen G. Soluble form of the (pro)renin receptor generated by intracellular cleavage by furin is secreted in plasma. Hypertension. 2009;53: 1077–1082. doi:10.1161/HYPERTENSIONAHA.108.127258

120. Nguyen G, Danser AHJ. Prorenin and (pro)renin receptor: a review of available data from *in vitro* studies and experimental models in rodents. Exp Physiol. 2008;93: 557–563. doi:10.1113/expphysiol.2007.040030

121. Morimatsu M, Syuto B, Shimada N, Fujinaga T, Yamamoto S, Saito M, et al. Isolation and characterization of bovine haptoglobin from acute phase sera. J Biol Chem. 1991;266: 11833–7. Available: http://www.ncbi.nlm.nih.gov/pubmed/1904872

122. Lee C-W, Cheng T-M, Lin C-P, Pan J-P, Pan J-P. Plasma haptoglobin concentrations are elevated in patients with coronary artery disease. PLoS One. 2013;8: e76817. doi:10.1371/journal.pone.0076817

123. Levy AP, Levy JE, Kalet-Litman S, Miller-Lotan R, Levy NS, Asaf R, et al. Haptoglobin genotype is a determinant of iron, lipid peroxidation, and macrophage accumulation in the atherosclerotic plaque. Arterioscler Thromb Vasc Biol. 2007;27: 134–140. doi:10.1161/01.ATV.0000251020.24399.a2

124. Wang F, Yang Y, Li P. [A case-control study on the risk factors of neural tube defects in Shanxi province]. Zhonghua Liu Xing Bing Xue Za Zhi. 2008;29: 771–774.

125. Horton JD, Goldstein JL, Brown MS. SREBPs: activators of the complete program of cholesterol and fatty acid synthesis in the liver. J Clin Invest. 2002;109: 1125–1131. doi:10.1172/JCI15593

126. Streicher R, Kotzka J, Müller-Wieland D, Siemeister G, Munck M, Avci H, et al. SREBP-1 mediates activation of the low density lipoprotein receptor promoter by insulin and insulin-like growth factor-I. J Biol Chem. 1996;271: 7128–7133. doi:10.1074/jbc.271.12.7128

127. Li Y, Xu S, Mihaylova MM, Zheng B, Hou X, Jiang B, et al. AMPK phosphorylates and inhibits SREBP activity to attenuate hepatic steatosis and atherosclerosis in diet-induced insulin-resistant mice. Cell Metab. 2011;13: 376–388. doi:10.1016/J.CMET.2011.03.009

128. Tang J-J, Li J-G, Qi W, Qiu W-W, Li P-S, Li B-L, et al. Inhibition of SREBP by a small molecule, betulin, improves hyperlipidemia and insulin resistance and reduces atherosclerotic plaques. Cell Metab. 2011;13: 44–56. doi:10.1016/J.CMET.2010.12.004

129. Motoyama K, Fukumoto S, Koyama H, Emoto M, Shimano H, Maemura K, et al. SREBP inhibits VEGF expression in human smooth muscle cells. Biochem Biophys Res Commun. 2006;342: 354–360. doi:10.1016/j.bbrc.2006.01.139

130. Chen W-J, Zhang M, Zhao G-J, Fu Y, Zhang D-W, Zhu H-B, et al. MicroRNA-33 in atherosclerosis etiology and pathophysiology. Atherosclerosis. 2013;227: 201–208. doi:10.1016/j.atherosclerosis.2012.11.025

131. Horie T, Nishino T, Baba O, Kuwabara Y, Nakao T, Nishiga M, et al. MicroRNA-33b knock-in mice for an intron of sterol regulatory element-binding factor 1 (Srebf1) exhibit reduced HDL-C in vivo. Sci Rep. 2015;4: 5312. doi:10.1038/srep05312

132. Nishino T, Horie T, Baba O, Sowa N, Hanada R, Kuwabara Y, et al. SREBF1/microRNA-33b axis exhibits potent effect on unstable atherosclerotic plaque formation in vivo. Arterioscler Thromb Vasc Biol. 2018;38: 2460–2473. doi:10.1161/ATVBAHA.118.311409

133. Privratsky JR, Paddock CM, Florey O, Newman DK, Muller WA, Newman PJ. Relative contribution of PECAM-1 adhesion and signaling to the maintenance of vascular integrity. J Cell Sci. 2011;124: 1477. doi:10.1242/JCS.082271

134. Bagi Z, Frangos JA, Yeh J-C, White CR, Kaley G, Koller A. PECAM-1 mediates NO-dependent dilation of arterioles to high temporal gradients of shear stress. Arterioscler Thromb Vasc Biol. 2005;25: 1590–1595. doi:10.1161/01.ATV.0000170136.71970.5f

135. Go G-W, Mani A. Low-density lipoprotein receptor (LDLR) family orchestrates cholesterol homeostasis. Yale J Biol Med. 2012;85: 19–28.

136. Brænne I, Kleinecke M, Reiz B, Graf E, Strom T, Wieland T, et al. Systematic analysis of variants related to familial hypercholesterolemia in families with premature myocardial infarction. Eur J Hum Genet. 2016;24: 191–197. doi:10.1038/ejhg.2015.100

137. Do R, Stitziel NO, Won H-H, Jørgensen AB, Duga S, Angelica Merlini P, et al. Exome sequencing identifies rare LDLR and APOA5 alleles conferring risk for myocardial infarction. Nature. 2015;518: 102–106. doi:10.1038/nature13917

138. Wang C, Tang Y, Wang Y, Li G, Wang L, Li Y. Label-free quantitative proteomics identifies Smarca4 is involved in vascular calcification. Ren Fail. 2019;41: 220–228. doi:10.1080/0886022X.2019.1591997

139. Liu Y, Wang T, Ji YJ, Johnson K, Liu H, Johnson K, et al. A C9orf72-CARM1 axis regulates lipid metabolism under glucose starvation-induced nutrient stress. Genes Dev. 2018;32: 1380–1397. doi:10.1101/gad.315564.118

140. Danese S, Vetrano S, Zhang L, Poplis VA, Castellino FJ. The protein C pathway in tissue inflammation and injury: pathogenic role and therapeutic implications. Blood. 2010;115: 1121–1130. doi:10.1182/blood-2009-09-201616

141. Lee SM, Nguyen D, Hu Z, Abbott GW. Kcne2 deletion promotes atherosclerosis and diet-dependent sudden death. J Mol Cell Cardiol. 2015;87: 148–151. doi:10.1016/j.yjmcc.2015.08.013
