# Supplementary material for: Prioritization of causal genes for coronary artery disease based on cumulative evidence from experimental and in silico studies
Source: Sci Rep. 2020 Jun 26;10:10486. doi: 10.1038/s41598-020-67001-w (PMC7320185; doi:10.1038/s41598-020-67001-w)
Supplement: Supplementary file 9 — Supplementary Data Legends. [file 41598_2020_67001_MOESM9_ESM.docx]

# ****Supplementary data legends****

## ****Supplementary Methods****

## ****Supplementary figure legends****

**Supplementary Figure S1.** A scheme depicting selection of CAD-associated loci for SMR/HEIDI analysis.

**Supplementary Figure S2.** A pipeline of extracting data from the previous studies on the genes potentially associated with CAD.

## ****Supplementary table legends****

**Supplementary Table S1.** CAD-associated loci selected for SMR/HEIDI analysis.

**Supplementary Table S1a.** Fifty loci selected from Howson et al. study (all ancestry).

**Supplementary Table S1b.** Seventeen loci selected from Nikpay et al. study.

**Supplementary Table S1c.** Final set of CAD-associated loci selected for SMR/HEIDI analysis.

**Supplementary Table S2.** Results of SMR/HEIDI analysis. Searching for pleiotropic effects of loci on CAD and gene expression.

**Supplementary Table S2a.** Results of SMR/HEIDI analysis. Searching for pleiotropic effects of the loci on CAD and gene expression. Associations that passed both SMR and HEIDI analyses (FDR_SMR_ < 0.05 and *P*_HEIDI_ ≥ 0.001).

**Supplementary Table S2b.** Results of SMR/HEIDI analysis. Searching for pleiotropic effects of the loci on CAD and gene expression. All associations.

**Supplementary Table S3.** Data on SNPs (located in 51 CAD-associated loci) which were linked to the prioritized genes.

**Supplementary Table S3a.** Linkage disequilibrium between top SNPs in SMR/HEIDI analysis (in loci where more than one top SNP were analyzed).

**Supplementary Table S3b.** Data on SNPs (located in the 51 studied loci) and the genes prioritized in the studies by Brænne et al., Lempiäinen et al., and van der Harst et al.

**Supplementary Table S3c.** Linkage disequilibrium between SNPs prioritized in the studies by Brænne et al., Lempiäinen et al., and van der Harst et al.

**Supplementary Table S3d.** Linkage disequilibrium between top SNPs in our SMR/HEIDI analysis and SNPs prioritized in the studies by Brænne et al., Lempiäinen et al., and van der Harst et al.

**Supplementary Table S4.** Genes in 51 CAD-associated loci (±250 kb around the lead SNP) proposed to be causal according to different lines of evidence.

**Supplementary Table S5.** Results of SMR & theta metric-based analysis. Searching for pleiotropic effects of the loci on CAD and gene expression.

**Supplementary Table S5a.** Results of SMR & theta metric-based analysis. Associations that passed both SMR and theta metric-based analyses (FDR_SMR_ < 0.05 and |theta| ≥ 0.7; number of SNPs in the theta metric-based analysis > 3).

**Supplementary Table S5b.** Results of SMR & theta metric-based analysis. Searching for pleiotropic effects of the loci on CAD and gene expression. All associations.

**Supplementary Table S6.** Results of colocalization analysis using the LocusCompare web tool. Searching for pleiotropic effects of the loci on CAD and gene expression.

**Supplementary Table S6a.** Results of colocalization analysis using the LocusCompare web tool. Loci with CAD GWAS lead SNP *P*-value < 5e-8 and eQTL lead SNP *P*-value < 1e-6. Associations with colocalization probability > 0.01.

**Supplementary Table S6b.** Results of colocalization analysis using the LocusCompare web tool. Searching for pleiotropic effects of the loci on CAD and gene expression. Loci with CAD GWAS lead SNP *P*-value < 5e-8 and eQTL lead SNP *P*-value < 1e-6. All associations.
